# Evidence for a High-Valent Iron-Fluoride That Mediates Oxidative C(sp^3^)-H Fluorination

**DOI:** 10.1021/jacsau.3c00021

**Published:** 2023-03-03

**Authors:** Chakadola Panda, Onyinyechukwuka Anny-Nzekwue, Lorna M. Doyle, Robert Gericke, Aidan R. McDonald

**Affiliations:** School of Chemistry and CRANN/AMBER Nanoscience Institute, Trinity College Dublin, The University of Dublin, College Green, Dublin 2, Ireland

**Keywords:** high-valent iron, fluorination, hydrogen atom transfer, proton coupled electron transfer, fluorine atom transfer

## Abstract

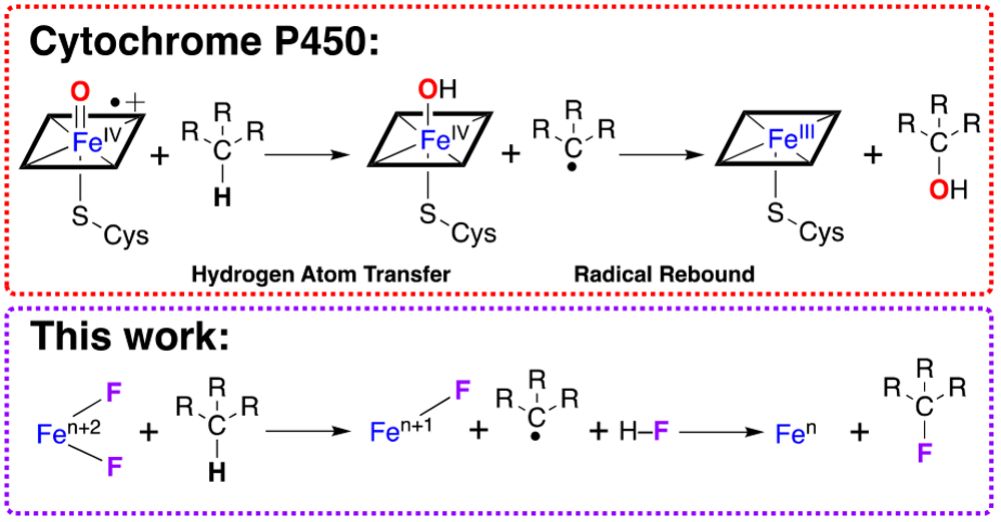

[Fe^II^(NCCH_3_)(NTB)](OTf)_2_ (NTB = tris(2-benzimidazoylmethyl)amine, OTf = trifluoromethanesulfonate) was reacted with difluoro(phenyl)-λ^3^-iodane (PhIF_2_) in the presence of a variety of saturated hydrocarbons, resulting in the oxidative fluorination of the hydrocarbons in moderate-to-good yields. Kinetic and product analysis point towards a hydrogen atom transfer oxidation prior to fluorine radical rebound to form the fluorinated product. The combined evidence supports the formation of a formally Fe^IV^(F)_2_ oxidant that performs hydrogen atom transfer followed by the formation of a dimeric μ-F–(Fe^III^)_2_ product that is a plausible fluorine atom transfer rebound reagent. This approach mimics the heme paradigm for hydrocarbon hydroxylation, opening up avenues for oxidative hydrocarbon halogenation.

## Introduction

The heme enzyme cytochrome P450 oxygenase selectively hydroxylates hydrocarbons under ambient conditions.^1−5^ Via the so-called heme paradigm, P450s activate O_2_ leading to a high-valent O=Fe^IV^-porphyrin-π-cation radical entity that cleaves strong C–H bonds through a hydrogen atom transfer (HAT) mechanism, which is a concerted form of proton coupled electron transfer (PCET).^2^ This results in the formation of HO–Fe^IV^-porphyrin and carbon-based radical products, which together undergo radical rebound (RR) to yield Fe^III^-porphyrin and hydroxylated product. Nonheme iron enzymes follow similar mechanistic pathways for the hydroxylation and halogenation of saturated alkanes.^6−10^ In the case of halogenation, an Fe–X (X = Cl, Br) undergoes RR with the formed carbon-based radical to yield the halogenated product.^8−11^ Synthetic X–Fe^IV^=O (X = F, Cl, Br) model complexes act as reliable mimics of nonheme iron halogenases, but suffer from competitive hydroxide rebound producing hydroxylated byproducts.^10,12,13^ Indeed theoretical calculations suggest that the hydroxide rebound is thermodynamically preferred over halide rebound.^14,15^ Recent breakthroughs have gone some way to addressing this issue.^16−18^ Ideally, an oxygen-free oxidant would be developed to ensure efficient halogenation.

Fluorine-containing molecules have attracted much attention because they display enhanced lipophilicity and metabolic stability.^19,20^ Furthermore, ^18^F radioisotope-labeled drug molecules are promising candidates for high resolution positron emission tomography (PET) imaging.^21,22^ Late-stage selective fluorination of saturated alkanes (thus C(sp^3^)–H fluorination) is extremely challenging owing to their relatively strong C–H bonds.^23−29^ C(sp^3^)–H fluorination strategies have been developed,^30−33^ however, the majority of the substrates used either contain activated carbon sites or need a directing group for facile fluorination.^31,34−36^ One-pot fluorination via formation of a carbon centered radical is a promising strategy for the preparation of aliphatic fluorides but has been limited to fluorine gas, XeF_2_, and hypofluorites, despite their high toxicity and cost.^37,38^ The free F^•^ radical generated from these oxidants is postulated to activate and fluorinate substrates with poor selectivity, which can be correlated to its very high redox potential (F^•^ → F^–^; *E*^0^ = 2.87 V).^39^ We believe that this can be modulated through bonding to a metal ion (M^*n*+^–F).

We have developed high-valent metal–halide (metal = Ni, Fe; halide = Cl, F) oxidants for PCET oxidation of inert hydrocarbons, demonstrating all such species to react via a HAT or concerted proton and electron (CPET) mechanism.^40−42^ We postulate that the thermodynamic driving force for HAT is the magnitude of the bond dissociation energy in the hydrohalic acid product (BDE_F–H_ = 135 kcal/mol, BDE_Cl–H_ = 103 kcal/mol).^43^ Herein, we postulate that metal-dihalides could demonstrate dual functionality: the first halide would facilitate PCET oxidation of hydrocarbons (yielding a reduced metal, carbon-centered radical, and hydrohalic acid) and the second halide would provide halogen atoms for RR. We present evidence for a putative high-valent Fe(F)_2_ that facilitates oxidative fluorination of saturated hydrocarbons via the postulated mechanism.

## Results and Discussion

### Oxidative Fluorination of Hydrocarbons

**Scheme 1:**
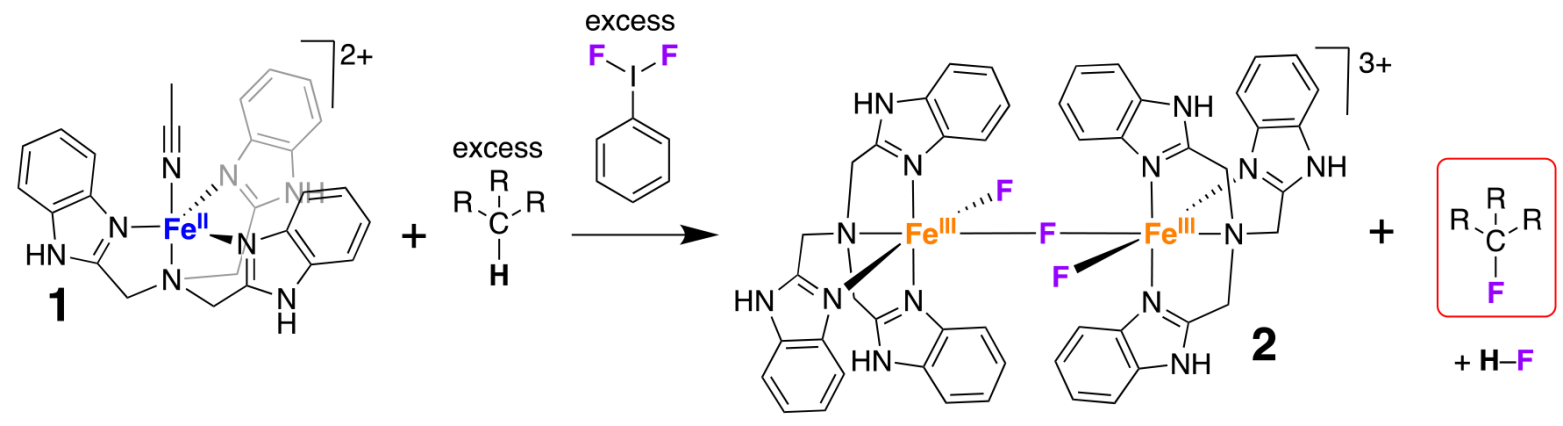
Complex **1**, Hydrocarbon Substrate, PhIF_2_, and Their Conversion to **2**, Fluorinated Product, and H–F

[Fe^II^(NCCH_3_)(NTB)](OTf)_2_ (**1**, NTB = tris(2-benzimidazolylmethyl)amine, OTf = trifluoromethanesulfonate) was prepared according to literature methods.^44,45^ The NTB ligand was selected because it had previously been demonstrated to yield highly reactive high-valent oxidants.^44,45^**1** (5 mM) and triphenylmethane (TPM, 50 mM) were combined in CH_3_CN under inert conditions. To this mixture was added difluoro(phenyl)-λ^3^-iodane (PhIF_2_, 10 mM, 2 equiv with respect to [**1**]). An immediate colorless to orange color change was observed. After stirring for 1 h at room temperature, the reaction mixture was analyzed by ^19^F NMR spectroscopy (Figure S1), which displayed three major peaks: δ = −74 ppm (assigned to free ^–^OTf counterion), δ = −182 ppm (assigned to HF in CH_3_CN),^46^ and δ = −126 ppm (assigned to trityl fluoride). The yield of trityl fluoride was determined to be 48% ([trityl fluoride]/[**1**]) according to ^19^F NMR. At higher PhIF_2_ equivalents (10 equiv), the fluorination yield was not improved, and indeed decreased. Furthermore, lower equivalents (≤1 equiv) showed lower fluorination yields (≤1%; Figures S2–S4). Analysis of a mixture of TPM and PhIF_2_ under the same conditions, but with no added Fe, demonstrated no formation of fluorinated products (Figure S5). **1** thus mediated the fluorination of an aliphatic C–H bond under inert conditions at room temperature in relatively high yields (Scheme 1).

We extended the substrate scope to hydrocarbons with varying C–H bond dissociation enthalpies (BDE_C–H_, from 77 to 96 kcal/mol).^47,48,49^ The reaction of **1** with PhIF_2_ in the presence of diphenylmethane, cumene, ethylbenzene, toluene, and adamantane all resulted in C–H bond fluorination in the hydrocarbons to varying degrees (Table 1, Figures S6–S12). Control reactions in the absence of **1** were carried out by exposing PhIF_2_ (10 mM) to excess substrates in CH_3_CN, under the same reaction conditions. In no case did we observe fluorinated product formation (Figures S13–S19). A comparison of the fluorination yields suggested that substrates with weak C–H bonds produced higher yields of fluorinated product than those with strong C–H bonds. For example, TPM and diphenylmethane were converted to the fluorinated products trityl fluoride and diphenyl-fluoro-methane, respectively, in >30% yield. In contrast, the yield of conversion of adamantane to 1-fluoro-admantane was just ∼2%. The oxidative fluorination of hydrocarbons was thus achieved for a variety of substrates using a combination of Fe^II^ complex **1** and PhIF_2_.

**Table 1:**
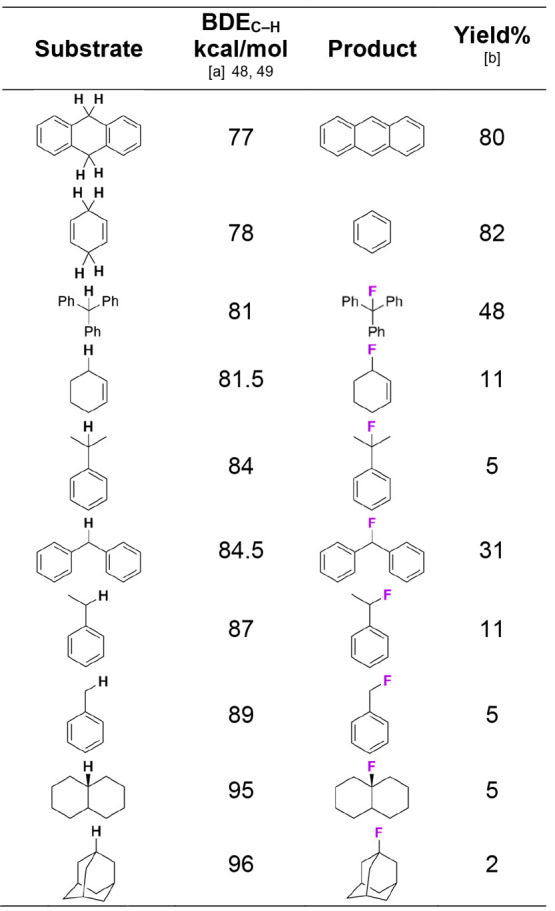
Hydrocarbon Oxidation/Oxidative Fluorination Using **1** and PhIF_2_

a The uncertainty in BDE_C–H_ value is ±1.

b The % yield of oxidations/fluorinations are with respect to [**1**] and were determined by ^19^F NMR (for fluorinated products) and GC-FID/^1^H NMR (for desaturated products).

When we explored the reaction between **1** and PhIF_2_ in the presence of 9,10-dihydroanthracene (DHA) or 1,4-cyclohexadiene (CHD), we observed no fluorinated products (Figures S20 and S21). We attribute this to the preference for aromatization (formation of anthracene from DHA and benzene from CHD) upon oxidation of these substrates rather than radical rebound with an F-donor. The major product obtained for DHA was anthracene (80% yield) as analyzed by gas chromatography (GC, Figure S22). The major product for CHD oxidation was benzene (82%) as analyzed by ^1^H NMR spectroscopy (Figures S23–S25). Analyses of the post-reaction mixtures for other substrates showed no oxygenated (alcohols, aldehydes, ketones, acids) or desaturated (olefins) products. This ruled out any competitive oxygenation/desaturation pathways occurring in the course of fluorination.

**Figure 1:**
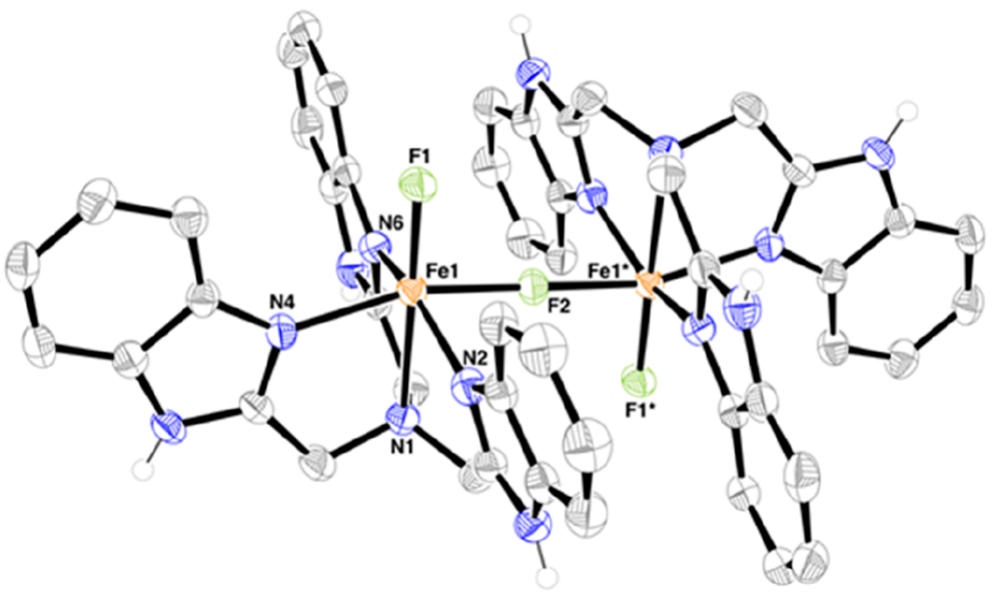
ORTEP of the tricationic core of **2**. Carbon bound hydrogen atoms, solvent molecules, and ^–^OTf anions were omitted for clarity. Thermal ellipsoids given at 50% probability level.

For all substrates, an orange coloration was observed at the end of the reactions. Even without any substrate, we observed the same orange solution after the reaction between **1** and PhIF_2_. Crystals suitable for single crystal X-ray diffraction analysis were acquired from a post-reaction mixture of **1** and PhIF_2_ that was layered with toluene. X-ray crystallography showed the product (defined as **2**) to be an (F)Fe^III^–F–Fe^III^(F) complex (Figure 1). **2** crystallized as [(μ–F){Fe(F)(NTB)}_2_](OTf)_3_·1.9NCCH_3_ in the monoclinic crystal system. **2** displayed terminal and bridging fluoride ligands. The terminal Fe1–F1 bond length was 1.816(2) Å, whereas the bridging Fe1–F2 bond length was 1.983(1) Å. Similar bond lengths have been previously assigned to terminal and bridging Fe–F bonds, respectively.^50−53^ The F2 atom sits on a center of inversion leading to an Fe1–F2–Fe1* angle of 180°. We recently reported a structurally very similar (F)Fe^III^–F–Fe^III^(F) complex supported by tris(2-pyridylmethyl)amine (TPA).^42^ In that case, the terminal Fe–F bonds were 1.826(2) Å, and the bridging Fe–F bonds were 1.957(1) Å. Fe^III^–O–Fe^III^ dimers supported by the same NTB ligand and containing two terminal chloride ligands have also been isolated and crystallographically characterized where the bridging oxo ligand serves as the center of inversion.^54,55^ However, the bridging Fe–O bond lengths (1.8050(9) Å) were significantly shorter than the bridging Fe–F bonds in **2** owing to the stronger bond between Fe and the dianionic O-ligand

The ^19^F NMR spectrum of **2** exhibited a signal at δ = −79 ppm and δ = −182 ppm that were assigned to free ^–^OTf and H–F in CD_3_CN, respectively (Figure S26). The free H–F is either derived from **2** upon solvation or residual H–F in the crystal lattice. No signals that could be attributed to the Fe-bound F-ligands were identified, presumably because they are bound to paramagnetic metal ions. ^1^H NMR of **2** displayed two paramagnetically shifted signals at δ = 53 and 46 ppm, whereas at least 10 signals would be expected (Figure S27). Several resonances were observed in the diamagnetic region, which might be associated with the metal-bound ligands. The absence of more paramagnetically shifted peaks could be because of significant broadening of peaks caused by the paramagnetic ion effect of the individual Fe^III^ centers. ESI-MS analysis of **2** displayed four major peaks with *m*/*z* = 241.06, 481.11, 501.11, and 631.07 corresponding to the molecular ions [Fe(F)(NTB)]^2+^, [Fe(F)(NTB) – H]^+^, [Fe(F)_2_(NTB)]^+^ and [Fe(F)(OTf)(NTB)]^+^, respectively (Figures S28–S32). These ions likely derived from the parent species **2** under the mass spectrometry conditions. Similar fragmentation under ESI-MS conditions has been observed for an analogues μ-F–Fe^III^_2_ complex.^42^ The Fourier transform infrared (FT-IR) spectrum of **2** displayed bands at ν = 744, 640, 592, 517 cm^–1^ which could be assigned to terminal and bridging Fe–F stretches in line with recent reports (Figure S33).^42,52,56^ The N–H stretches were observed at ν = 3050 cm^–1^. **1** was thus converted to (F)Fe^III^–F–Fe^III^(F) complex **2** upon reaction with PhIF_2_ in the presence and absence of hydrocarbon substrates.

**Scheme 2:**
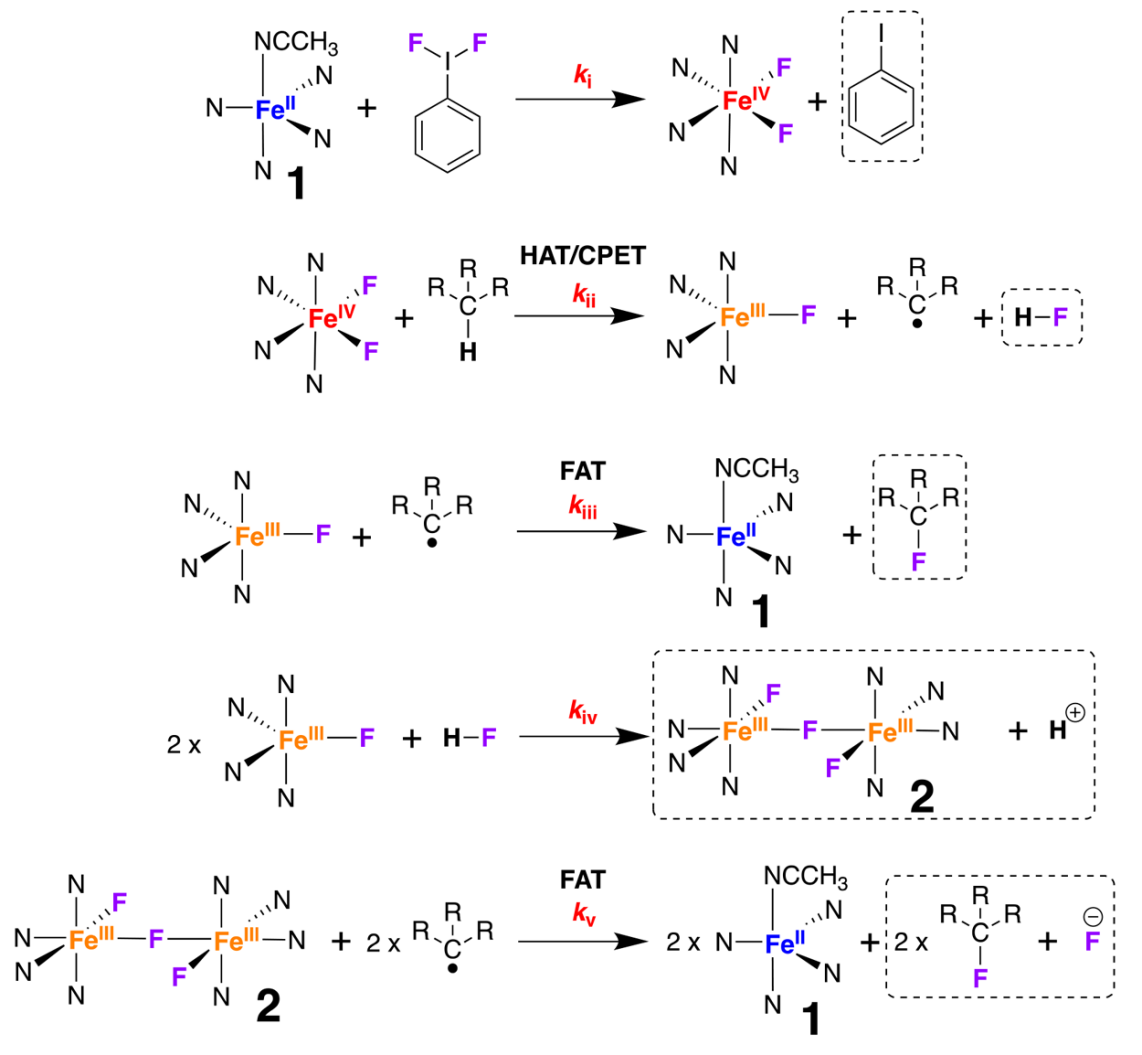
Postulated Mechanism for Formation of the Active Oxidant, the HAT/CPET Step, and the FAT Step in Hydrocarbon Fluorination Notes: (a) Items in dashed boxes have been identified in the post-reaction mixture; (b) **1** was not identified in the post-reaction mixture, the reformed **1** is surmised to be re-oxidised, because there is an excess of PhIF_2_.

We considered the mechanism of formation of **2**, fluorinated product, and H–F in the reaction between **1** and PhIF_2_ in CH_3_CN (Scheme 2). Our goal was to obtain mechanistic insight in order to understand the active oxidant in C–H activation and the F atom transfer reagent. We identified five possible kinetic steps: the formation of a high-valent adduct; PCET oxidation; radical rebound by an Fe^III^–F adduct to yield fluorinated product and H–F; and dimerization of Fe^III^–F adducts. The depicted mechanism is corroborated by our mechanistic investigations (detailed below), which also allowed us to exclude other potential oxidants and fluorinating agents. Given the potential complexity of any kinetic analysis of this system, we decided a simple approach was warranted. The only reliable kinetic handle we could probe was the rate of formation of **2**, as detailed below.

**Figure 2:**
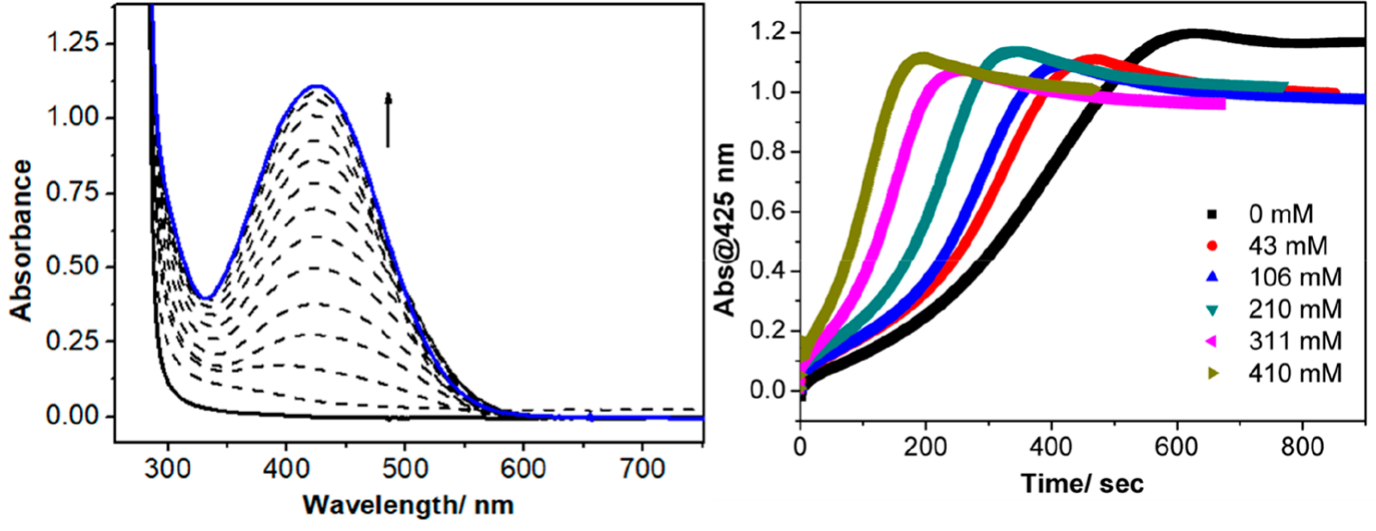
Left: Electronic absorption spectral changes for the reaction of **1** (0.5 mM, black solid trace) and PhIF_2_ (2 equiv) in CH_3_CN at 0 °C in the absence of substrate, yielding **2** (blue trace). Right: Time trace for absorption changes at λ = 425 nm vs time for the same reaction in the presence of toluene under varying concentrations of toluene (black: 0 mM; red: 43 mM; blue: 106 mM; green: 210 mM; pink: 311 mM; brown: 410 mM).

The progress of the reactions between **1**, hydrocarbon, and PhIF_2_ were monitored using electronic absorption spectroscopy (Figure 2). A typical reaction involved cooling a solution of **1** (0.5 mM, CH_3_CN) to 0 °C, followed by addition of PhIF_2_ (2 equiv), resulting in the formation of a new chromophore at λ = 425 nm. The electronic absorption spectrum of **2** displayed the same absorbance maximum at λ = 425 nm (ε = 4100 M^–1^ cm^–1^, Figure S34). The novel chromophore was thus assigned to **2** through comparison with the spectrum measured for isolated **2** (Figure S35). When the same procedure was followed in the presence of a hydrocarbon substrate, the electronic absorption spectral changes were the same (formation of λ = 425 nm was observed, Figures S36–S43).^57^ However, and critically, the rate of formation of **2** was accelerated when substrates were present and was furthermore dependent on the substrate concentration. Using toluene as an example, in the absence of substrate, the reaction between **1** and PhIF_2_ yielded **2** within 600 s. In contrast, when 800 equiv of toluene were present, the reaction was complete within 200 s (Figure 2). The yield of **2** was consistently quantitative (±10%), that is, all **1** was converted into **2**, independent of the substrate concentration and/or substrate BDE_C–H_.

The profile of the kinetic trace for the formation of **2** appeared to be multiphasic (indicating multiple processes, Figure 2).^58^ In order to obtain kinetic insight into the fluorination of hydrocarbons and the formation of **2**, we decided to relate the overall rate of formation of **2** to the properties of the added substrate. When the reciprocal of the time required for the maximum formation of **2** (thus *k*_obs_) was plotted against substrate concentration, we observed a linear dependency for all substrates (Figures S37–S43). This showed that the rate of formation of **2** was first order in substrate. The substrate was present in large excess (for all substrates tested). This led us to treat *k*_obs_ as a pseudo-first-order rate constant. In summary, the rate of formation of **2** was linearly dependent on [substrate], indicating that the rate of the overall reaction was first order in substrate.

**Figure 3:**
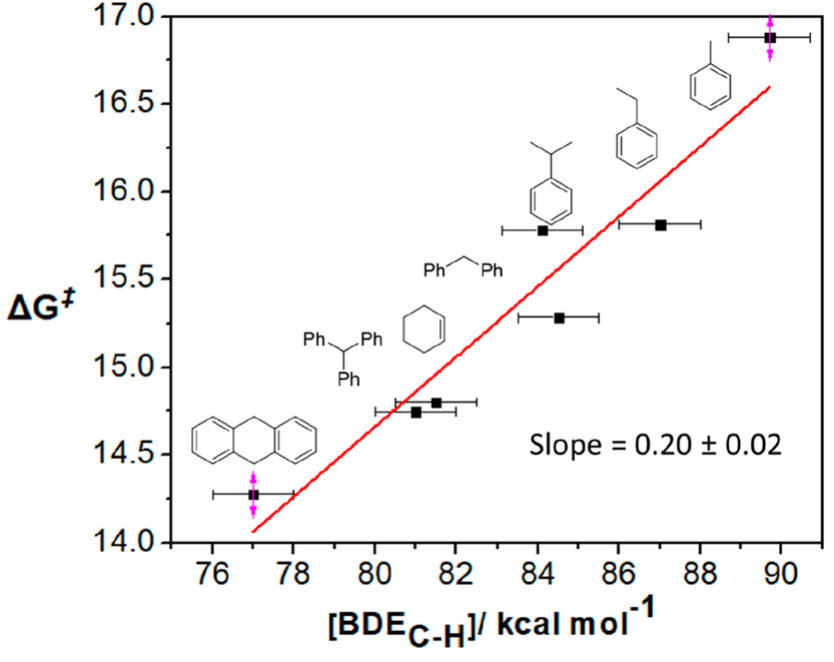
Plot of Δ*G*^⧧^ vs BDE_C–H_ for the preparation of **2** from the reaction of **1** with PhIF_2_ in the presence of hydrocarbon substrates. Δ*G*^⧧^ values were calculated from the corresponding *k*_2_ using the Eyring equation. As bond dissociation free energy (BDFE_C–H_) values for all the substrates in CH_3_CN are not available, we have plotted the BDE_C–H_ (an enthalpy) as a surrogate.

The slopes of the *k*_obs_ versus [substrate] plots were calculated to give second-order reaction rate constants (*k*_2_) for hydrocarbon activation. In calculating *k*_2_ values, we made the assumption that the reaction was first order in the high-valent oxidant. Fascinatingly, the *k*_2_ values were relatable to the magnitude of the BDE_C–H_ of the substrate. We observed a linear correlation between BDE_C–H_ and log(*k*_2_) (Figure S44). When the free energy of activation (Δ*G*^⧧^), as calculated from the respective *k*_2_ values using the Eyring equation,^59,60^ was plotted against the BDE_C–H_ of the substrate, a linear dependency was observed with a measured slope of 0.20 (a Bell–Evans–Polyani plot, Figure 3). The slope of this plot can provide insight into the mechanism of PCET reactions. A slope of approximately 0.5 is indicative of HAT or CPET mechanism (defined as HAT for brevity from now on). The measured slope is slightly deviated from 0.5, such deviations have also been observed for high-valent metal-halides and M=O,^40−42,61−63^ indicating that a HAT mechanism is operative in the present case. The observation of a relationship between both the concentration of hydrocarbon substrate and the magnitude of BDE_C–H_ of the substrate and the rate of formation of **2** would suggest an involvement of HAT oxidation of the substrate during the formation of **2**.

We monitored the reaction between **1** and PhIF_2_ in the presence of a deuterated substrate (toluene-*D*_8,_Figure S45). Using the same kinetic analysis for the formation of **2**, we measured a kinetic isotope effect (KIE) of 7. This further supported the involvement of a proton transfer or hydrogen atom transfer from the hydrocarbon substrate in the formation of **2**. This KIE value is very close to the classical value (2–7) that is expected for a typical PCET reaction.^64^ Similar KIE values were also recently observed for high-valent metal oxidants thereby stressing the proton/H-atom involvement.^40,61,65−72^

In the absence of substrate, we assume that the solvent gets oxidized in analogous fashion to the substrates. To probe this, we performed the reaction between **1** and PhIF_2_ in CH_3_CN and CD_3_CN and explored any differences in the rate of formation of **2** (Figure S46). There was an insignificant difference between the rates in CH_3_CN and CD_3_CN at 0 °C. However, when the reactions were performed at lower temperature (−20 and −40 °C), significantly different rates of formation of **2** were observed. This suggests that the activation of solvent C–H/D bonds were involved in the formation of **2**, providing further support for a transient highly reactive oxidant.

We also analyzed the initial 60 seconds of the reaction between **1** and PhIF_2_ for each substrate (*k*_obs_′, Figure S47), obtaining initial rate constants (*k*_1_′) for the formation of **2** in the presence of the various substrates. Interestingly, a linear relationship of *k*_obs_′ versus [substrate] was observed, and log(*k*_1_′) versus BDE_C–H_ also provided a linear dependency with a slope of −0.20. This observations is analogous to that obtained in the Bell–Evans–Polyani plot, suggesting an HAT mechanism was also operating in the initial phase of the reaction (Figure S48).

In summary, the rate of formation of **2** was linearly dependent on [substrate]. Second, the rate of formation of **2** was also linearly dependent on the magnitude of BDE_C–H_. Finally, when deuterated substrate or solvent was employed, the rate of formation of **2** decreased, with a KIE of ∼7. Based on these results we conclude that a HAT mechanism was operative in the fluorination reaction and in the formation of **2**, confirming the formation of an oxidizing entity upon reaction of **1** with PhIF_2_. When considering the various kinetic steps that could influence the rate of formation of **2**, only one of them (HAT by a high-valent oxidant) would be impacted by the changes in [substrate], changes in the magnitude of substrate BDE_C–H_, and substrate H/D exchange. In terms of understanding how the C–H bond was activated, we were therefore interested *only* in this kinetic step.

We postulate (Scheme 2) that PhIF_2_ reacted with Fe^II^**1** to yield a formally Fe^IV^(F)_2_ complex and iodobenzene (which was identified in the post-reaction ^1^H NMR, Figure S25). The formally Fe^IV^(F)_2_ species is surmised to oxidize the substrate via HAT to yield a carbon-based radical, Fe^III^–F, and H–F. We postulate that mononuclear Fe^III^–F, **2**, H–F, or excess PhIF_2_ then rebounds with the carbon-based radical to yield the fluorinated product. We also postulate that **2** forms from the combination of two mononuclear Fe^III^–F entities and F^–^ (from H–F). Below we describe experiments to probe this postulated mechanism.

### Hydrogen Atom Transfer Reagent

The observation of a signal that was assigned to H–F in the ^19^F NMR (Figure S1) of the post-reaction mixture provides support for a PCET event by an X–F entity in the hydrocarbon oxidation reactions. H–F could be derived from PCET oxidation where X–F is Fe–F, PhIF_2_, or an F-atom radical (F^•^). As detailed above, PhIF_2_ was not capable of fluorination of any of the listed substrates. Below we probe the role of Fe–F species and of F^•^.

We recently reported that [Fe^III^(F)(TPA)]^2+^ was capable of HAT oxidation of substrates with low BDE_C–H_.^42^ [Fe^III^(F)(TPA)]^2+^ was formed from the reaction of [μ-F–(Fe^III^(F)(TPA))_2_]^3+^ with Lewis or Bro̷nsted acids. We therefore assessed the hydrocarbon oxidation reactivity of **2** and its Lewis/Bronsted acid activated derivates. There was no reaction between **2** and DHA according to electronic absorption spectroscopy (Figure S49). DHA has a very low BDE_C–H_ value, lower than any of the substrates (that were fluorinated) tested above (Table 1), showing that **2** is not the active oxidant in the oxidative fluorination of hydrocarbons by **1** and PhIF_2_.

Addition of Sc(OTf)_3_ (4 equiv) to a solution of **2** (0.12 mM, CH_3_CN) at 0 °C showed the feature at λ = 425 nm immediately shifted to λ = 505 nm along with a broadening of the peak (Figure S50). This new species was labeled as **3**. The half-life of **3** was 6.5 h. The ESI-MS of **3** exhibited a major peak at *m*/*z* = 241.06 corresponding to the dicationic species [Fe(F)(NTB)]^2+^ alongside mass peaks for [Fe(F)(NTB)(NCCH_3_)]^2+^ (*m*/*z* = 282.21), not observed in the mass spectrum of **2** (Figure S51). This indicates the formation of a mononuclear Fe^III^–F species. The ^1^H NMR spectrum of **3** displayed paramagnetically shifted signals at δ = −16 and −12 ppm and the ^19^F NMR spectrum exhibited a signal from the triflate anions (Figures S52–S53). The resonances for **3** were shifted from low field to high field, when compared to **2**. The same observation was made when [μ-F–(Fe^III^(F)(TPA))_2_]^3+^ was activated to yield [Fe^III^(F)(TPA)]^2+^.^42^ The EPR spectrum of **3** was dominated (>80%) with a rhombic signal with *g* = 8.67, 4.29 characteristic of a high-spin (*S* = 5/2) mononuclear Fe^III^ species (Figure S54).^6,7,73,74^ The EPR of the dimer **2** was silent, indicating it does not interconvert with mononuclear Fe^III^ species (Figure S55). Interestingly, a post-reaction mixture containing **1** and PhIF_2_ was also EPR silent (Figure S56). Thus the combined spectroscopic analyses lead us to conclude that, in the presence of a Lewis acid, the dimer **2** broke into a mononuclear [Fe^III^(F)(NTB)]^2+^ (**3**).

**3** reacted with DHA according to electronic absorption spectroscopy (Figure S57). The product of the reaction was anthracene with the characteristic electronic features at λ = 375, 365, 345 nm. **3** was not able to activate stronger C–H bonds, for example in toluene, where no reaction was observed (Figure S58). We thus conclude that **3** is not the active oxidant in the oxidative fluorination of hydrocarbons by **1** and PhIF_2_. We conclude that the active oxidant in oxidative fluorination by **1** and PhIF_2_ is likely a formally Fe^IV^(F)_2_ species and is not an Fe^III^–F entity.

The use of selectfluor (SF, 1-(chloromethyl)-4-fluoro-1,4-diazabicyclo[2.2.2]octane-1,4-diium ditetrafluoroborate) as an alternative oxidant/fluorinating reagent was also examined. We observed (again) the formation of fluorinated products, as well as **2** (λ_max_ = 425 nm), from the reaction of **1** and SF (2 equiv) albeit at considerably slower reaction rates (Figure S59). As with the reaction between **1** and PhIF_2_, multiphasic kinetic behavior was observed in the reaction between **1** and SF. This led us to conclude that an adduct of Fe and PhIF_2_ was more-than-likely *not* the active oxidant, given that the same outcomes could be obtained with PhIF_2_ and an alternative F-atom donor (SF).

An F^•^ radical is an alternative oxidant. Indeed, photo-eliminated Cl^•^ radicals from parent metal-chloride catalysts are proposed to carryout HAT reactivity, although with significantly less selectivity than we have observed.^75−79^ Typically HAT by simple radicals (X^•^) would contribute negligibly to the net reorganization energy and hence Δ*S*^⧧^ would be close to zero. However, HAT reactions involving transition-metal complexes display large reorganizational energy resulting in significant entropic (Δ*S*^⧧^ ≫ 0) contributions as demonstrated by Mayer and co-workers.^80,81^ We therefore measured the activation parameters for the reaction between **1**, toluene, and PhIF_2_ using the same methods to calculate the *k*_obs_ values (Figure S60). By performing an Arrhenius plot (−10 to +10 °C), we determined Δ*H*^⧧^ = 15 ± 1 kcal mol^–1^ and Δ*S*^⧧^ = −16 ± 3 cal mol^–1^K^–1^ for the HAT involved in the formation of **2**. Large and negative Δ*S*^⧧^ values suggested that the HAT entity is more likely an Fe complex rather than F^•^. This is also consistent with our *selective* monofluorination of substrates (cyclohexene, ethylbenzene, cumene, adamantane, etc.) in contrast to nonselective halogenation for photo-eliminated Cl^•^.^78^ In summary, the evidence reasonably supports a formally Fe^IV^(F)_2_ species as the active oxidant, with the combined evidence excluding Fe^III^–F, PhIF_2_, or F^•^.

### Fluorine Radical Rebound Reagent

Having explored the oxidant involved in oxidative fluorination by **1**, we attempted to decipher how a C–F bond was formed, by probing the fluorine atom transfer (FAT) reactivity of **2**, **3**, and PhIF_2_. Gomberg’s dimer (Scheme S1, a disguised trityl radical) was identified as a useful probe, mirroring the putative trityl radical formed in the reaction between **1**, PhIF_2_, and TPM. Upon addition of Gomberg’s dimer (10 equiv) to **2** (0.5 mM, CH_3_CN, −40 °C), the characteristic features at λ = 425 nm for **2** decayed within 600 s (Figure S61). The product of the reaction was characterized by ^19^F NMR, showing that trityl fluoride had formed (δ = −127 ppm, 48% yield; Figure S62). In the post-reaction mixture [Fe^II^(NCCH_3_)(NTB)]^2+^ (**1**) was identified by ^1^H NMR spectroscopy (Figure S63).

First-order rate fitting was applied to obtain the pseudo-first-order rate constant (*k*_obs_ = 5 × 10^–3^ s^–1^). The concentration of Gomberg’s dimer was further varied, and the plot of the *k*_obs_ versus the respective concentrations of the substrate allowed us to obtain *k*_2_ = 1.24 M^–1^ s^–1^. Fe^III^–X (X= F, Cl, Br, OCH_3_, and OH) species are known to react with carbon radicals to form the corresponding C–X bond.^82−84^ To the best of our knowledge, **2** represents the first Fe^III^_2_ complex for radical rebound. A comparison of the *k*_2_ values for X-atom transfer reactivity by terminal Fe^III^–X suggests that **2** was several orders of magnitude less effective toward FAT than terminal Fe^III^–X species (Table S1).^85^

**2** was also reacted with triphenylphosphine (PPh_3_). Upon addition of PPh_3_ to **2**, the absorbance feature at λ = 425 nm decreased with a concomitant increase at λ = 370 nm indicating a reaction had occurred (Figure S64). The ^19^F NMR spectrum of the post-reaction mixture displayed a doublet centered at δ = −38 ppm and the ^31^P NMR spectrum showed a triplet at δ = −54 ppm. These signals are characteristic of the difluorinated oxidized product F_2_PPh_3_ (Figures S65–S68). F_2_PPh_3_ was obtained in 60% yield. **2** was thus a highly capable FAT reagent and represents a plausible candidate for F-atom rebound in the reaction between **1**, PhIF_2_, and hydrocarbons.

We then examined **3** for FAT to Gomberg’s dimer. To our surprise, we did not observe any trityl fluoride (Figure S69). We are unsure of the impact of the added Sc^III^ ions on FAT reactivity and, therefore, cannot rule out mononuclear Fe^III^–F adducts as the FAT reagent in the fluorination of hydrocarbons by **1** and PhIF_2_. Finally, we found that PhIF_2_*was* capable of FAT to Gomberg’s dimer (Figure S70). However, we did not just identify trityl fluoride from this reaction, but also a number of arene-fluorination signals by ^19^F NMR. PhIF_2_ can attack multiple carbon-centered radicals that are in resonance with the trityl radical/Gomberg’s dimer, thereby leading to a distribution of fluorinated products (Figure S71). This contrasts markedly with **2**, which delivered F-atoms selectively at the tertiary radical site. In summary, **2** is a plausible F-atom donor alongside currently undefined mononuclear Fe^III^–F adducts.

A final consideration is the yield of fluorinated product obtained in the reaction between **1**, PhIF_2_, and hydrocarbons. There are two variables that would impact the yield of fluorination: (1) how much carbon radical was formed by HAT; and (2) the lifetime of the formed radical. The results to hand do not provide sufficient insight to draw any conclusions. The trityl radical, for example, is long-lived, in contrast to purely aliphatic substrates that likely have short-lived radicals (e.g., adamantane), which may explain why TPM is fluorinated in high yields and adamantane is not. However, adamantane also displays a markedly higher BDE_C–H_ than TPM, which may impact the yield of carbon radical if competitive solvent C–H activation occurs (for example). We tentatively conclude that the lifetime of the alkyl radical is the determining factor in whether rebound will occur. In the case of short-lived radicals, other RR events may occur (solvent or ligand H-atom abstraction), whereas in the case of long-lived radicals fluorination rebound may occur to yield a strong C–F bond. PhIF_2_ concentration also appeared to play an important role in determining the yield of fluorinated product (Figures S2–S4). The yield of fluorinated products varied widely, from low at low [PhIF_2_] to ∼50% at 2 equiv of PhIF_2_ to low again at high [PhIF_2_]. We attribute the low fluorination yields at low [PhIF_2_] to a lower concentration of rebounding FAT entities in the reaction medium–lower concentrations of Fe–F will result in lower yields of FAT. It should also be noted that the rate of the reaction is very slow at low [PhIF_2_] (Figure S36). We attribute the low fluorination yields at high [PhIF_2_] to slower rates of HAT oxidation. We have previously observed that excess halides inhibit PCET oxidation by metal-halides.^40−42^ At higher [PhIF_2_], we surmise the effect is similar to having higher [halide] and HAT by Fe^IV^F_2_ is thus inhibited resulting in lower fluorination yields.

## Conclusion

We present an Fe-mediated one-pot aliphatic hydrocarbon fluorination methodology through the oxidative fluorination of C–H bonds by the combination of aliphatic hydrocarbons with a nonheme Fe^II^ complex (**1**) and PhIF_2_. Detailed kinetic and product analyses point toward a HAT rate-determining step that occurs prior to fluorine rebound to a carbon based radical to form the fluorinated product. The combined evidence supports the formation of a transient formally Fe^IV^(F)_2_ oxidant, from the reaction of Fe^II^ and PhIF_2_, that performs HAT followed by the formation of a dimeric μ-F–(Fe^III^)_2_ product (**2**). **2** was extensively characterized and found to readily deliver F-atoms to carbon radicals and triphenylphosphine, indicating that it is a plausible candidate for F-atom rebound in the fluorination of hydrocarbons. Our approach takes inspiration from the heme paradigm for hydrocarbon oxidation, delivering oxidatively fluorinated products without competition from oxygenated side-products.

## Methods

All synthetic and kinetic experimental procedures were performed under inert conditions using standard Schlenk techniques or inside an mBraun inert atmosphere glovebox containing an atmosphere of purified nitrogen. Dry solvents were dispensed from an mBraun SPS-800 solvent purification system and degassed for 20 min before use by purging with dry N_2_.

**Caution**! *Hydrofluoric acid (HF) was identified as a product of the reactions listed above. HF is a highly corrosive inorganic acid. Therefore, reactions with the potential for formation of HF must be handled with extreme caution. HF can penetrate the skin extremely easily and decalcifies bones, leading to tissue necrosis, which may result in amputation and death. Solutions as weak as 1% can still rapidly permeate the skin and severely damage underlying tissues. The maximum concentration of HF that can be formed throughout this work was capped at 0.017%. Furthermore, all the locally defined protective measures for working with metal-fluorides and HF were followed for all experiments.*

## Materials

Reagents necessary for the synthesis of the tris(2-benzimidazolylmethyl)amine (NTB) ligand and its Fe^II^ complex were obtained from Sigma-Aldrich and/or FluoroChem. The substrates (9,10-dihydroanthracene, 1,4-cyclohexadiene, triphenylmethane, diphenylmethane, cyclohexene, *cis*-decalin, cumene, ethylbenzene, toluene, toluene-*d*_8_ and adamantane) were also obtained from commercial sources and purified via recrystallization for solids and distillation for liquids. Liquid substrates such as cumene, ethylbenzene, cyclohexene, and toluene were passed through activated basic alumina before distillation was done. The NTB ligand and its Fe^II^ complex were prepared as per literature procedures.^44,45^ Difluoro(phenyl)-λ^3^-iodane (PhIF_2_) was prepared according a literature methodology and purity was confirmed by ^19^F NMR spectroscopy.^86^ Gomberg’s dimer was synthesized as per literature procedures.^87^

### Synthesis of [μ-(F)-(Fe^III^-(F)(NTB))_2_] (**2**)

A typical synthesis involved dissolution of solid [Fe^II^(NCCH_3_)(NTB)](OTf)_2_ (**1**, 80 mg, 1 equiv, 0.1 mmol) and solid PhIF_2_ (48 mg, 2 equiv, 0.2 mmol) in separate CH_3_CN solutions (1 mL each) in the Glovebox. After complete dissolution, the solutions were combined and stirred for 10 min at room temperature. The colorless solution of **1** changed to orange. Then toluene (0.5 mL) was added to the reaction mixture, and the resulting solution was stored for crystallization at −35 °C in a glovebox freezer. Over a period of 1 week orange crystals formed. The mother liquor was decanted off and the crystals were washed three times with diethyl ether (Et_2_O) (3 × 2 mL) to yield **2** (96 mg, 67% yield; 0.067 mmol). UV–vis (CH_3_CN); λ_max_: 425 nm (ε = 4100 M^–1^cm^–1^). δ_H_ (ppm) (400 MHz, ^1^H NMR in CD_3_CN): 53.4, 46.5 δ_F_ (ppm) (400 MHz, ^19^F NMR in CD_3_CN): −78.7. ESI-MS (*m*/*z*): [**2**] found: 241.06, 481.11, 501.11, and 631.07 corresponding to the expected masses of [Fe(F)(NTB)]^2+^, [Fe(F)(NTB-H)]^+^, [Fe(F)_2_(NTB)]^+^, and [Fe(F)(OTf)(NTB)]^+^, respectively. ν_max_ (FTIR)/cm^–1^: 3047, 1454, 1245, 1222, 1026, 920, 744, 640, 592, 516, 287, 226.

### UV–vis Kinetic Study

A solution of **1** (0.5 mM) and the desired amount of C–H substrate were taken in CH_3_CN in a septum sealed quartz cuvette at 0 °C under N_2_. To this cryostat stirred mixture was added PhIF_2_ (2 equiv, 100 μL of 20 mM stock solution in CH_3_CN) with continuous stirring. A new chromophore with absorbance maximum at λ = 425 nm (formation of compound **2**) was observed as the reaction proceeded. The kinetic traces could not be fitted with a typical first-order rate law. Hence the overall time taken for the formation was noted, and its reciprocal was considered as a first-order rate constant (*k*_obs_). At least five different concentrations of the substrate were reacted, and the second-order rate constants (*k*_2_) were calculated by plotting *k*_obs_ vs the concentration of the substrates. Each experiment was performed at least three times, and the average values of *k*_obs_ and *k*_2_ were reported.

The kinetic traces were also evaluated by assessing the rate of formation of **2** in the initial 60 s of the reaction. In both cases, we observed a linear dependency of the rate of formation of **2** against the substrate concentration.

### Generation of **3** and Its HAT Reactivity

A solution of **2** (0.1 mM) in CH_3_CN was taken in a septum sealed quartz cuvette at 0 °C under inert conditions. To this was added Sc(OTf)_3_ (4 equiv) while stirring. As a result of a reaction, a new chromophore with absorbance maximum at λ = 505 nm (formation of compound **3**) appeared. Species **3** displayed a dark violet color. After maximum formation of **3**, varied equivalents of the substrates (DHA or toluene) were added. The decay at λ = 505 nm was monitored over time. The first-order rate law was applied to obtain the pseudo-first order rate constants (*k*_obs_). At least five different concentrations of the substrate were reacted, and the second-order rate constants (*k*_2_) were calculated by plotting *k*_obs_ vs the concentration of the substrates. Each experiment was performed at least three times, and the average values of *k*_obs_ and *k*_2_ were presented.

### FAT Reactivity

A solution of **2** (0.1 mM) in CH_3_CN in a septum sealed quartz cuvette at −40 °C was prepared under inert conditions. To this stirred mixture the desired volumes of Gomberg’s dimer were added. As a result of reaction the characteristic feature at λ = 425 nm for complex **2** decayed over time. The first-order rate law was applied to obtain the pseudo-first-order rate constants (*k*_obs_) for the decay of **2**. At least five different concentrations of the substrate were reacted, and the second-order rate constants (*k*_2_) were calculated by plotting *k*_obs_ vs the concentration of the substrates. Each experiment was performed at least three times and the average values of *k*_obs_ and *k*_2_ were presented. The reaction with triphenylphosphine was carried out at room temperature (25 °C).

### Product Identification for HAT and FAT Reactivity

The post-reaction mixtures were injected into a GC calibrated with the authentic compounds (expected products of the reaction). The retention time and signal intensity were used to identify and quantify the product. In addition to GC, ^1^H, ^19^F, and ^31^P NMR experiments were performed to assess the products formed.

## Supporting Information Available

Experimental methods, kinetic data, and spectral data (EPR, FT-IR, GC, MS, NMR, UV–vis) for all new compounds, crystallographic data for **2** (PDF)
