# Supplementary material for: Evidence for a High-Valent Iron-Fluoride That Mediates Oxidative C(sp3)-H Fluorination
Source: JACS Au. 2023 Mar 3;3(3):919–28. doi: 10.1021/jacsau.3c00021 (PMC10052241; doi:10.1021/jacsau.3c00021)
Supplement: Supplementary file 1 — au3c00021_si_001.pdf [file au3c00021_si_001.pdf]

Evidence for a High-Valent Iron-Fluoride that Mediates Oxidative C(sp^3)-H Fluorination

Chakadola Panda,[1,2] Onyinyechukwuka Anny-Nzekwue,[1] Lorna M. Doyle,[1] Robert Gericke[1,3] and Aidan R. McDonald*[1]

[1]*School of Chemistry and CRANN/AMBER Nanoscience Institute, Trinity College Dublin, The University of Dublin, College Green, Dublin 2, Ireland*

[2]*Current address: Department of Chemistry, Science Faculty, University of Allahabad, Prayagraj, Uttar Pradesh 211002, India*

[3]*Current address: Helmholtz-Zentrum Dresden-Rossendorf e.V., Institute of Resource Ecology, Bautzner Landstraße 400, 01328 Dresden, Germany*

Email: aidan.mcdonald@tcd.ie

Physical methods:

Ultraviolet and visible (UV-vis) spectroscopy: UV-vis measurements were recorded on a Hewlett Packard Agilent 8453 UV-visible spectrophotometer (190-1100 nm range) equipped with an Unisoku cryostat system (USP-203; Unisoku, Japan) at programmed temperatures (0 and -40 °C).

Nuclear magnetic resonance spectroscopy (NMR): ^{1}H, ^{19}F, and ^{31}P NMR analyses were performed on an Agilent MR400 instrument. Deuterated solvents were used for ^{1}H NMR and chemical shifts (ppm) referenced against residual protic solvent signals. For quantification of fluorinated product, ^{19}F NMR integrations for two triflate anions (6F atoms) present in **1** were used as reference.

Electrospray ionization mass spectrometry (ESI-MS): ESI mass spectra were obtained using a Bruker microTOF-Q III spectrometer interfaced to a Dionex UltiMate 3000 LC in positive mode. The instrument calibration was done using a tune mix solution (Agilent Technologies ESI-l Low concentration tuning mix) which was also used as an internal lock mass. Samples dissolved in desired solvents were injected via direct infusion. Mmass software was used for data analyses.

Gas chromatography (GC): GC measurements were performed using a ThermoFisher TRACE^{M} 1300 gas chromatograph equipped with a flame ionization detector. Hydrogen was provided by a Parker Zero Air Generator UHP-10ZA-S.

Infrared spectroscopy: Attenuated total reflectance Fourier transform infrared (ATR-FTIR) spectra were recorded on a Bruker Tensor II Fourier transform infrared spectrometer.

Single-Crystal X-ray Diffraction Analysis: X-ray structural analysis for **2** was performed on a Bruker APEX-II CCD at 100(2) K with an Oxford Cryostream, with the sample mounted on a MiTeGen microloop using Mo K_{α} radiation (λ = 0.71073 Å). Bruker APEX[1] software was used to collect and reduce

data and determine the space group. Absorption corrections were applied using SADABS.[2] Structures were solved with the XT structure solution program[3] using Intrinsic Phasing and all non-hydrogen atoms were anisotropically refined in full-matrix least-squares cycles against |F2| (ShelXL).[4] Hydrogen atoms were placed in idealized positions and refined isotropically (riding model). Selected parameters of data collection and refinement of **2** are listed in Table S3. Crystallographic data for the structure in this paper have been deposited with the Cambridge Crystallographic Data Centre as supplementary publication CCDC: **2161335**. Copies of the data can be obtained, free of charge, on application to CCDC, 12 Union Road, Cambridge CB2 1EZ, UK, (fax: +44-(0)1223-336033 or e-mail: deposit@ccdc.cam.ac.uk).

X-band Electron paramagnetic Resonance (EPR) measurements: EPR spectra of **2** and **3** (5 mM each in CH_3CN) were performed on a Bruker EMX X-band EPR spectrometer equipped with liquid N_2 cooling. Freshly prepared samples were taken in a quartz EPR tube. The solution in the tube was frozen in liquid nitrogen and kept frozen until measured. The EPR spectra were recorded at 77 K, 9.29 GHz, 2.01 mW microwave power, with a 100 mT field sweep in 83.6 s, and 0.3 mT field modulation amplitude. Integration, simulation, and fitting was executed with Matlab and the easySpin computational package.[5] Spin quantification of the sample was performed by comparison of the double integral of the signal assigned to **2** and **3** to that of a frozen 10 mM methanol solution of iron-ethylenediaminetetraacetic acid (Fe^{III}-EDTA), measured under the same conditions. The system was modelled as a S = 5/2 species with g tensors; g = 8.53 and 4.24 and inhomogeneous line broadening; with 80% spin quantification. A minor species (<5%) at 2.24 was also observed that can correlated to a low spin (S = 1/2) species.

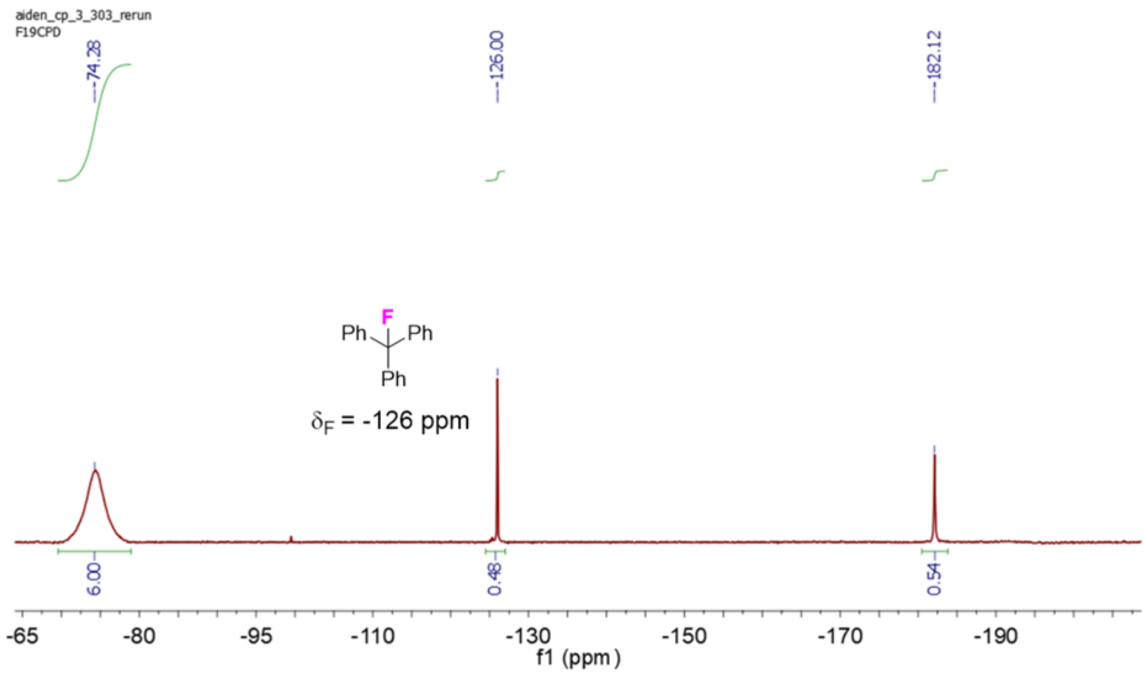

Figure S1. ^{19}F NMR of a reaction mixture containing **1** (5 mM), triphenylmethane (50 mM), and $PhIF_2$ (10 mM; 2 equiv.) in CD_3CN. The signal at δ = -126 ppm corresponds to the fluorinated product trityl fluoride (48% yield).

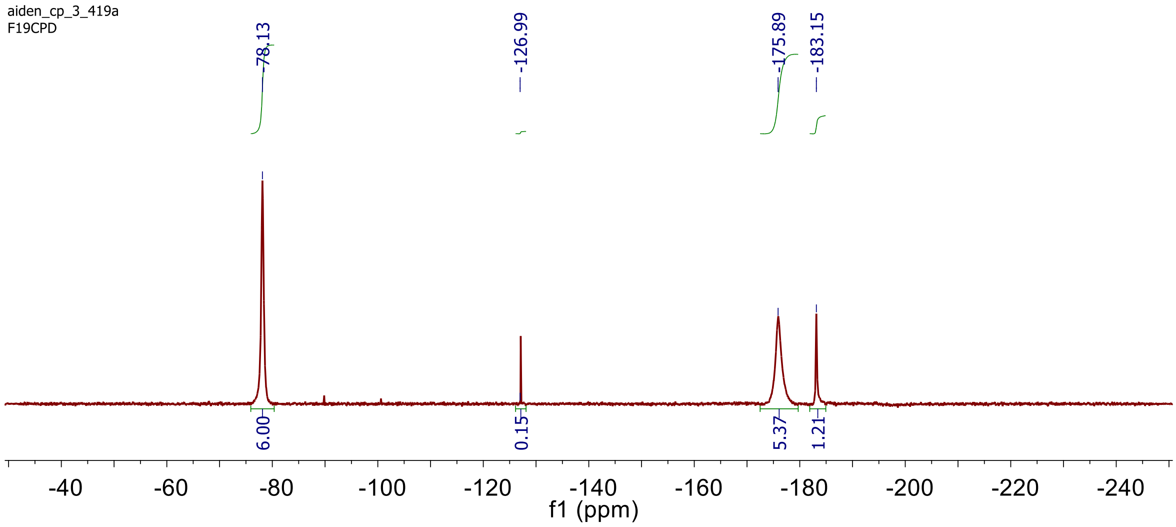

Figure S2. ^{19}F NMR of a reaction mixture containing **1** (5 mM), triphenylmethane (50 mM), and $PhIF_2$ (50 mM; 10 equiv.) in CD_3CN. The signal at δ = -127 ppm corresponds to the fluorinated product trityl fluoride (15% yield).

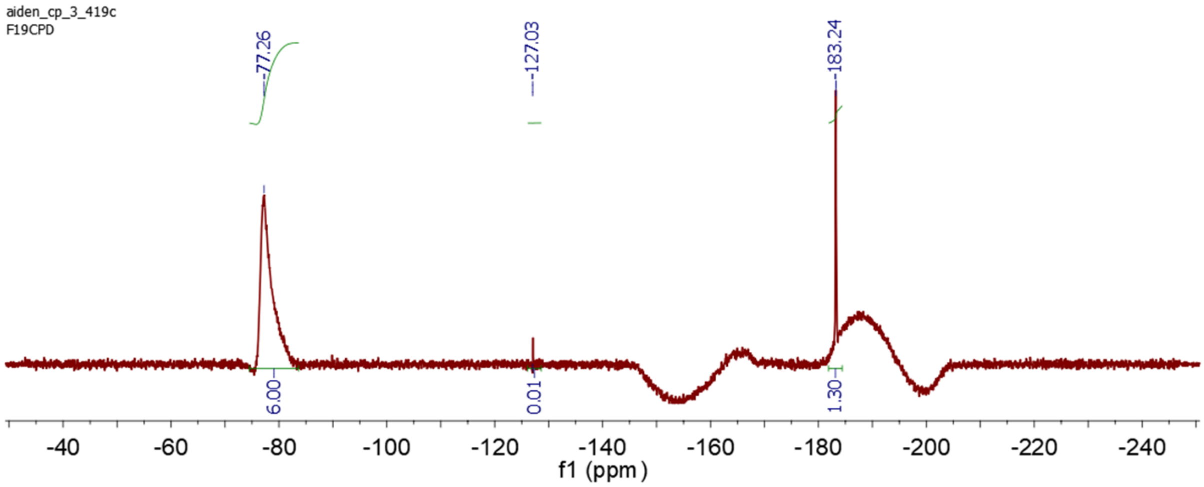

Figure S3. ^{19}F NMR of a reaction mixture containing **1** (5 mM), triphenylmethane (50 mM), and $PhIF_2$ (5 mM; 1 equiv.) in CD_3CN. The signal at δ = -127 ppm corresponds to the fluorinated product trityl fluoride (1% yield).

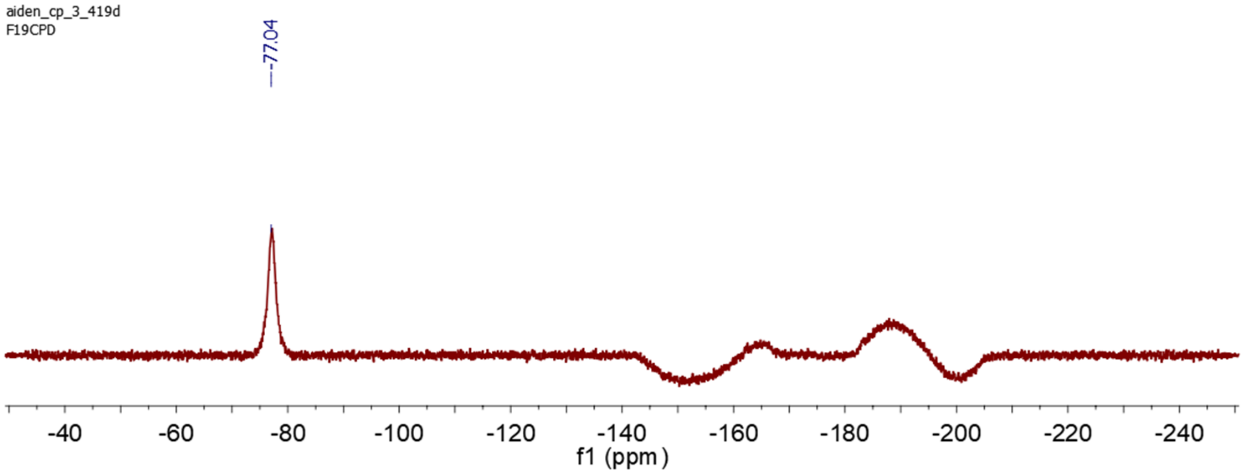

Figure S4. ^{19}F NMR of a reaction mixture containing **1** (5 mM), triphenylmethane (50 mM), and $PhIF_2$ (3.5 mM; 0.75 equiv.) in CD_3CN. The signal for trityl fluoride was not observed indicating no fluorination has occurred.

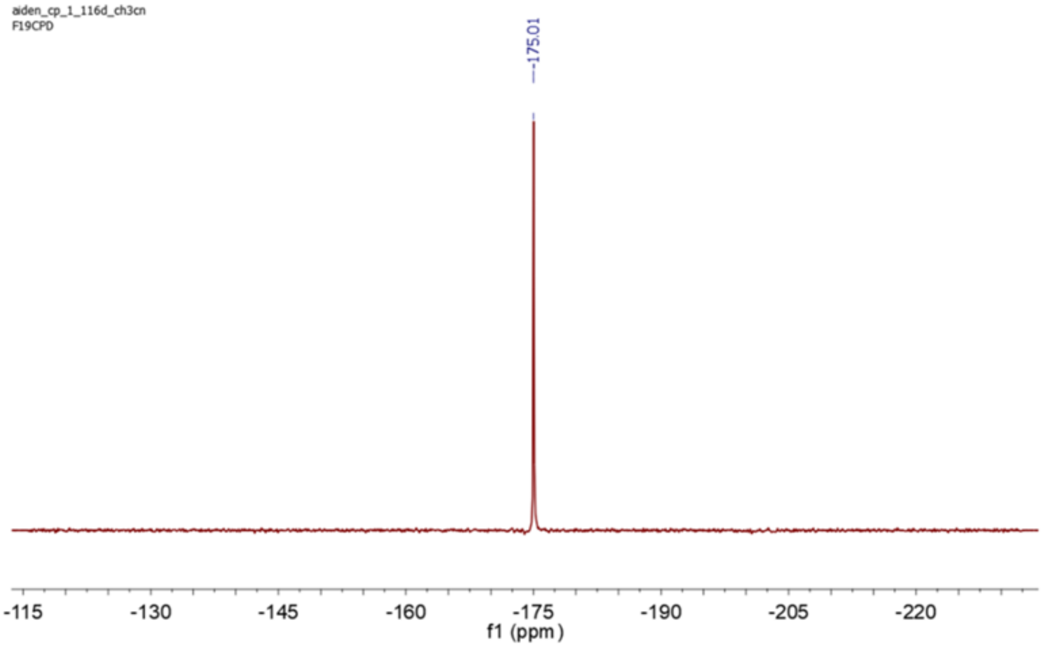

Figures S5. ^{19}F NMR of a reaction mixture containing triphenylmethane (50 mM) and $PhIF_2$ (10 mM) in CD_3CN. The signal at δ = -175 ppm corresponds to unreacted $PhIF_2$.

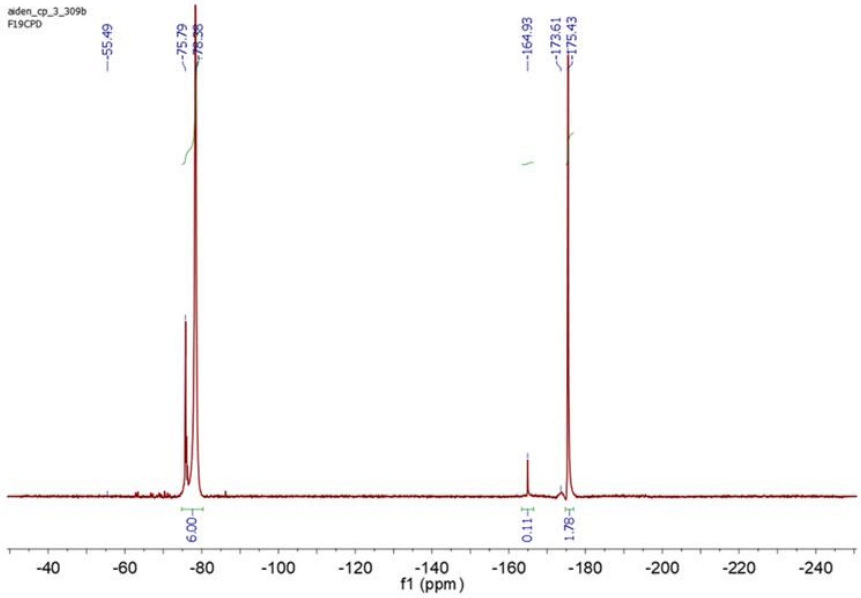

Figure S6. ^{19}F NMR of a reaction mixture containing **1** (5 mM), cyclohexene (1 M), and $PhIF_2$ (10 mM) in CD_3CN. The signal at δ = -165 ppm corresponds to the fluorinated product 3-fluorocyclohex-1-ene (11% yield).

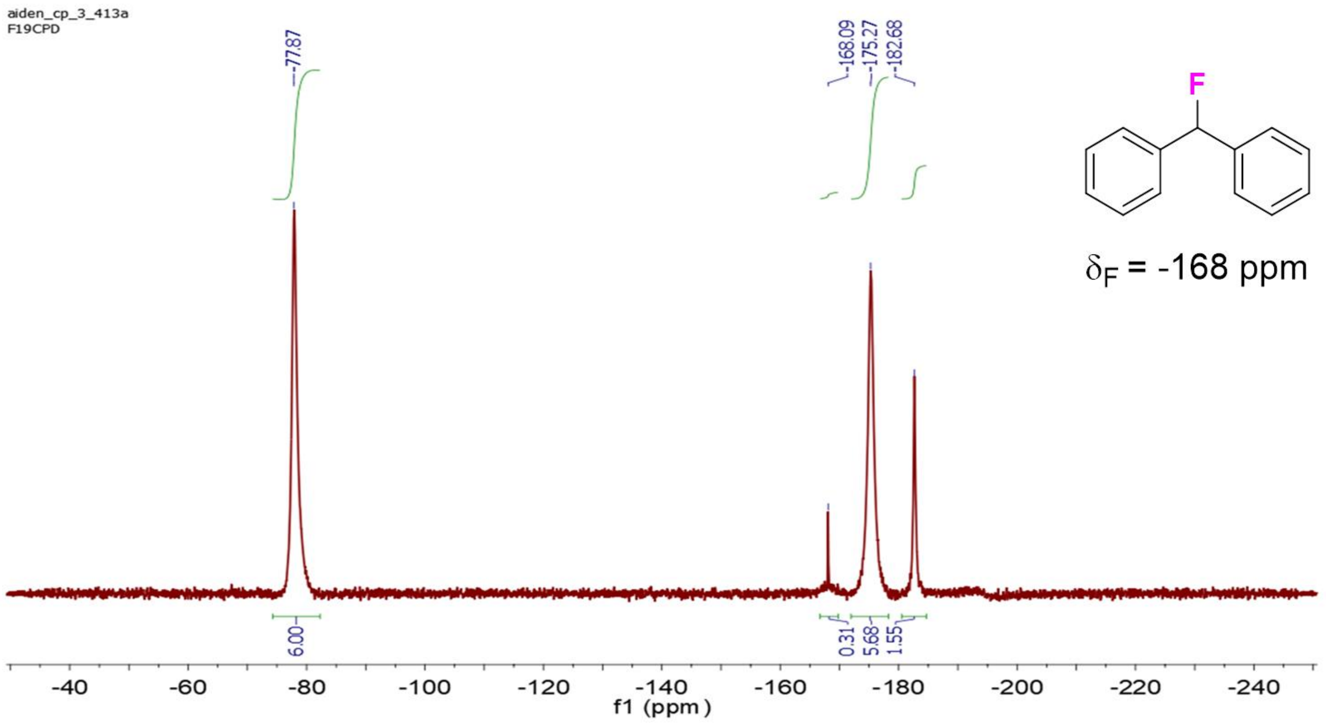

Figures S7. ^{19}F NMR of a reaction mixture containing **1** (5 mM), $PhIF_2$ (10 mM), and diphenylmethane (500 mM) in CD_3CN. The signal at δ = -168.09 ppm corresponds to the fluorinated product diphenyl-fluoro-methane (31% yield).

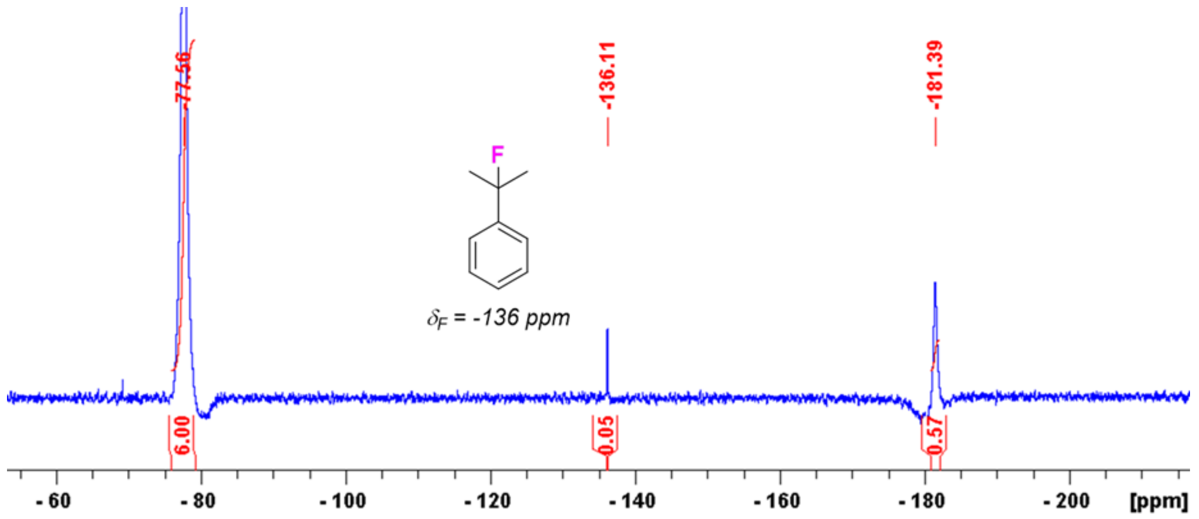

Figure S8. ^{19}F NMR of a reaction mixture containing **1** (5 mM), $PhIF_2$ (10 mM), and cumene (800 mM) in CD_3CN. The signal at δ = -136 ppm corresponds to the fluorinated product (2-fluoropropan-2-yl)benzene (5% yield).

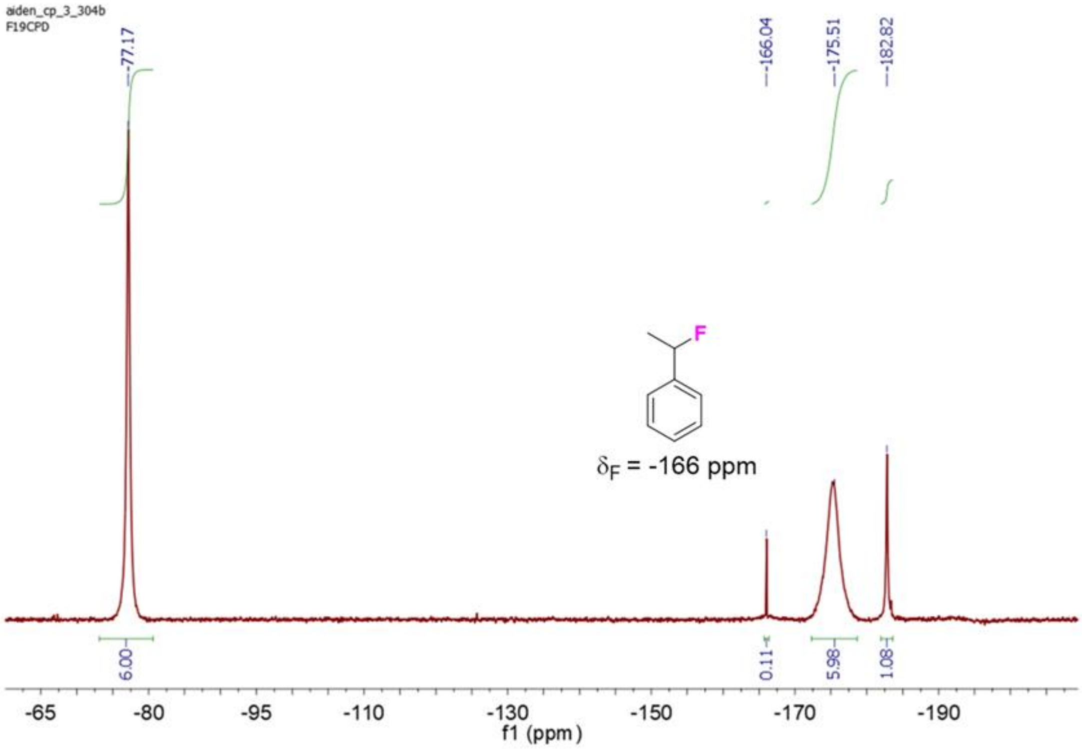

Figure S9. ^{19}F NMR of a reaction mixture containing **1** (5 mM), $PhIF_2$ (10 mM), and ethylbenzene (800 mM) in CD_3CN. The signal at δ = -166.04 ppm corresponds to the fluorinated product (1-fluoroethyl)benzene (11% yield).

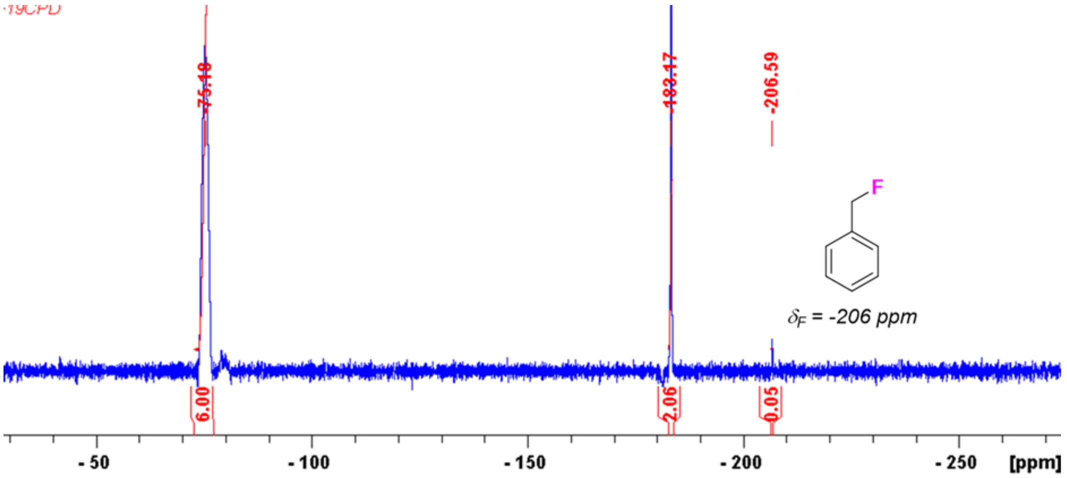

Figure S10. ^{19}F NMR of a reaction mixture containing **1** (5 mM), $PhIF_2$ (10 mM) and toluene (800 mM) in CD_3CN. The signal at δ = -206 ppm corresponds to the fluorinated product (fluoromethyl)benzene (5% yield).

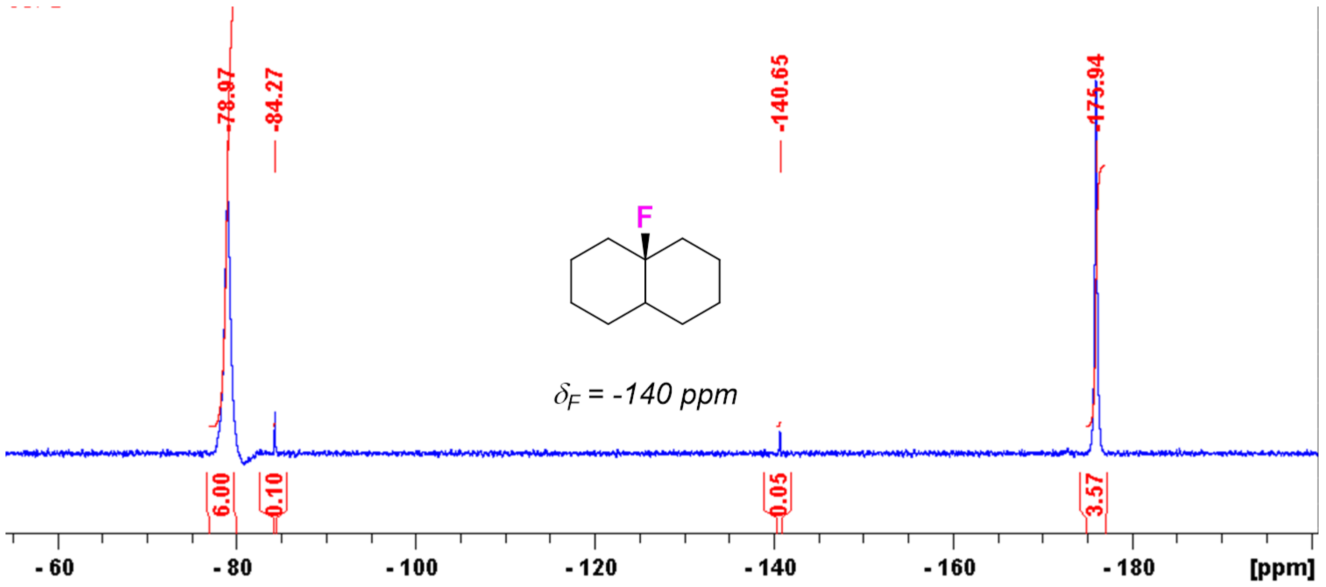

Figure S11. ^{19}F NMR of a reaction mixture containing **1** (5 mM), $PhIF_2$ (10 mM), and *cis*-decalin (800 mM) in CD_3CN. The signal at δ = -140 ppm corresponds to the fluorinated product (4a-fluorodecahydronaphthalene (5% yield).

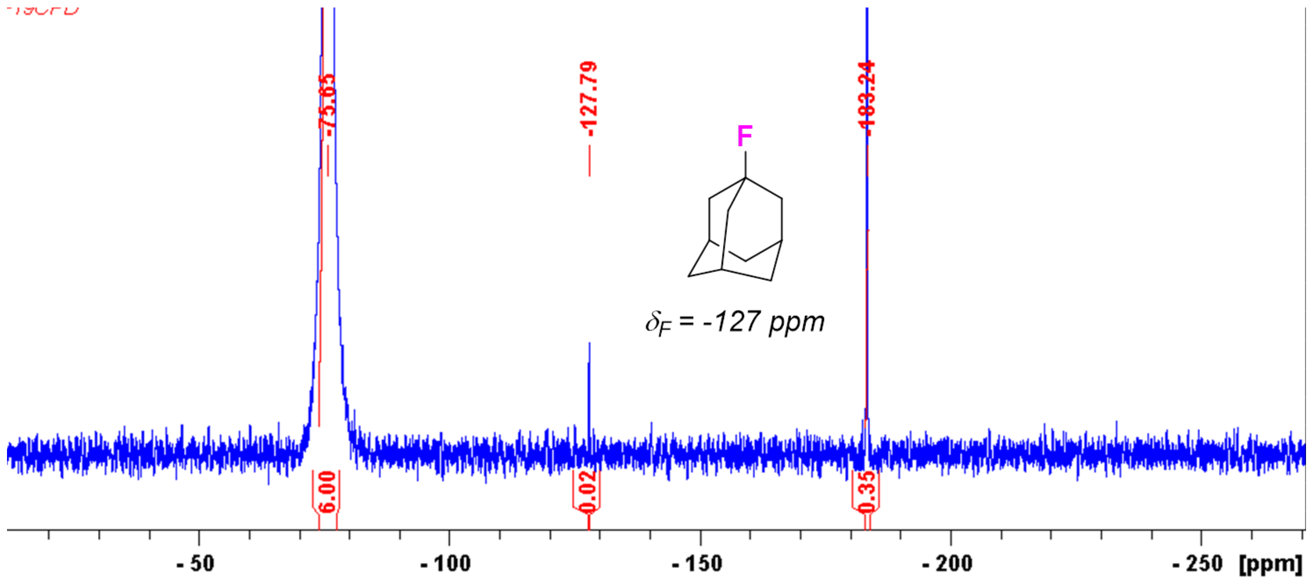

Figure S12. ^{19}F NMR of a reaction mixture containing **1** (5 mM), $PhIF_2$ (10 mM), and adamantane (50 mM) in CD_3CN. The signal at δ = -127 ppm corresponds to the fluorinated product 1-fluoroadamantane (2% yield).

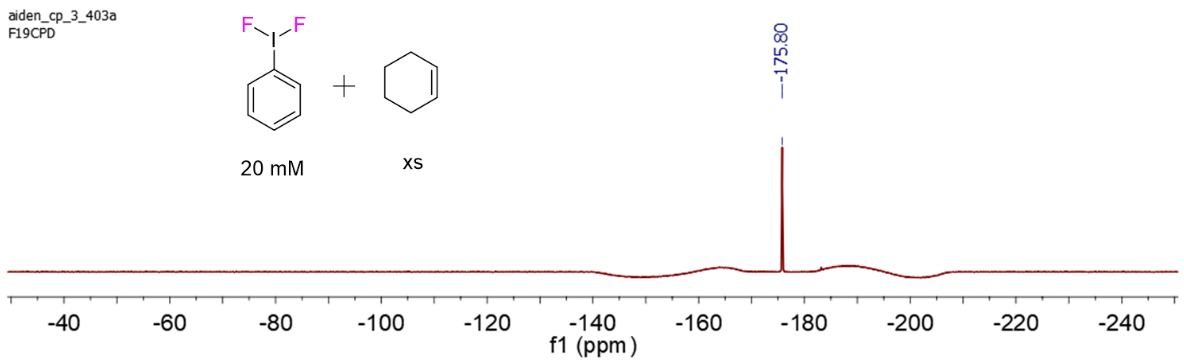

Figure S13. ^{19}F NMR of a reaction mixture containing cyclohexene (1 M) and $PhIF_2$ (10 mM) in CD_3CN. The signal at δ = -175 ppm corresponds to unreacted $PhIF_2$.

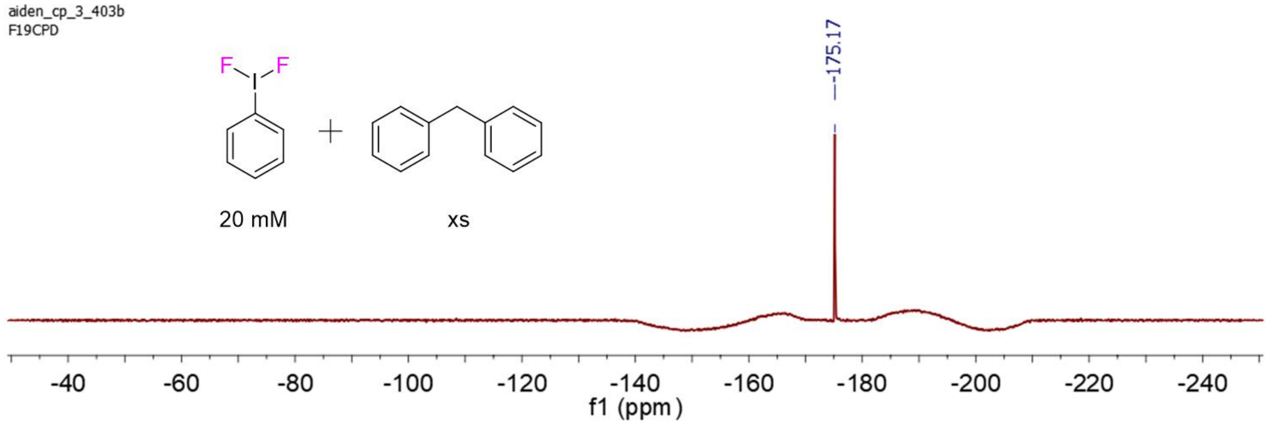

Figure S14. ^{19}F NMR of a reaction mixture containing diphenylmethane (500 mM) and $PhIF_2$ (10 mM) in CD_3CN. The signal at δ = -175 ppm corresponds to unreacted $PhIF_2$.

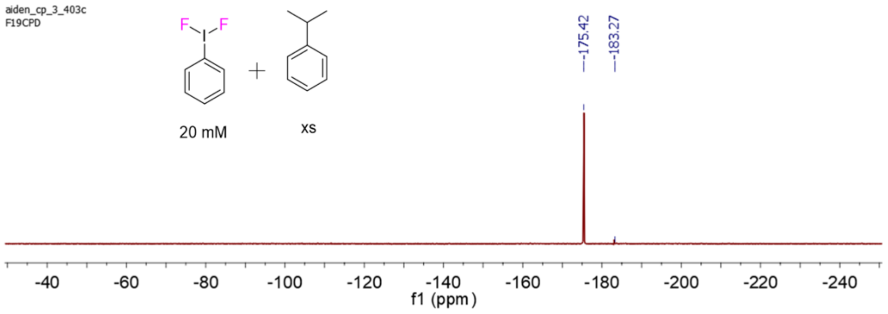

Figure S15. ^{19}F NMR of a reaction mixture containing cumene (500 mM) and $PhIF_2$ (10 mM) in CD_3CN. The signal at δ = -175 ppm corresponds to unreacted $PhIF_2$.

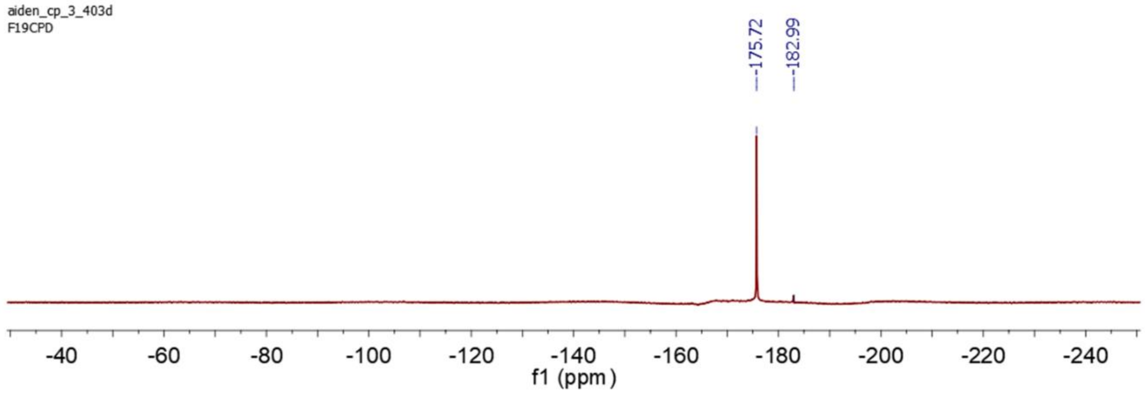

Figure S16. ^{19}F NMR of a reaction mixture containing *cis*-decalin (500 mM) and $PhIF_2$ (10 mM) in CD_3CN. The signal at δ = -175 ppm corresponds to unreacted $PhIF_2$.

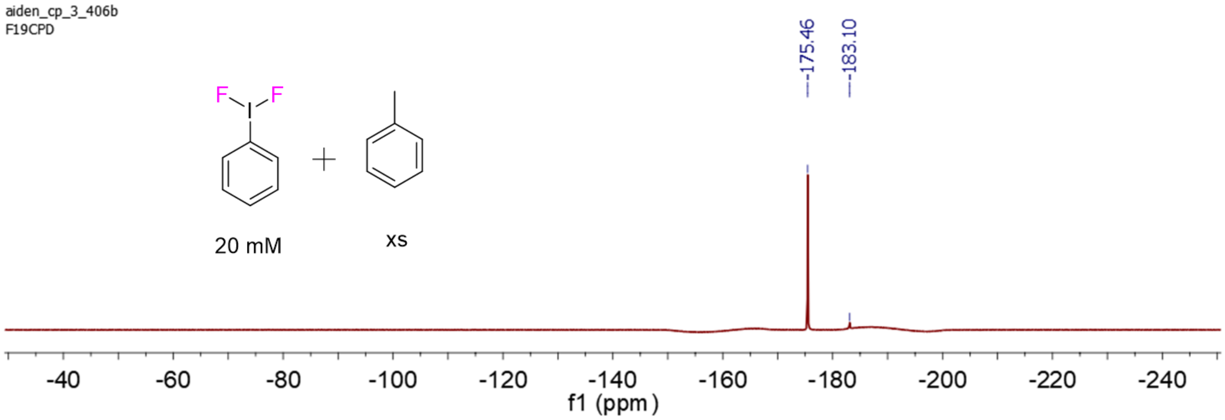

Figure S17. ^{19}F NMR of a reaction mixture containing toluene (500 mM) and $PhIF_2$ (10 mM) in CD_3CN. The signal at δ = -175 ppm corresponds to unreacted $PhIF_2$.

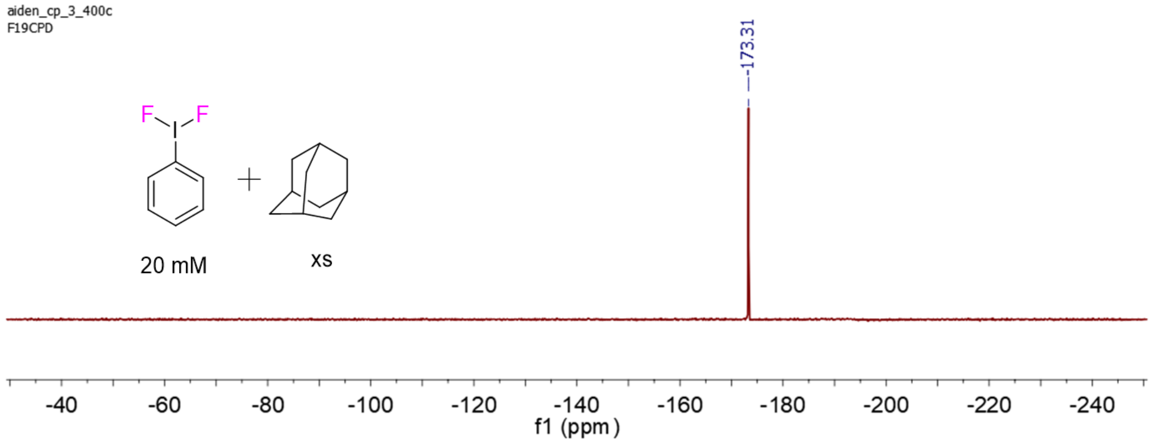

Figure S18. ^{19}F NMR of a reaction mixture containing adamantane (50 mM) and $PhIF_2$ (10 mM) in CD_3CN. The signal at δ = -175 ppm corresponds to unreacted $PhIF_2$.

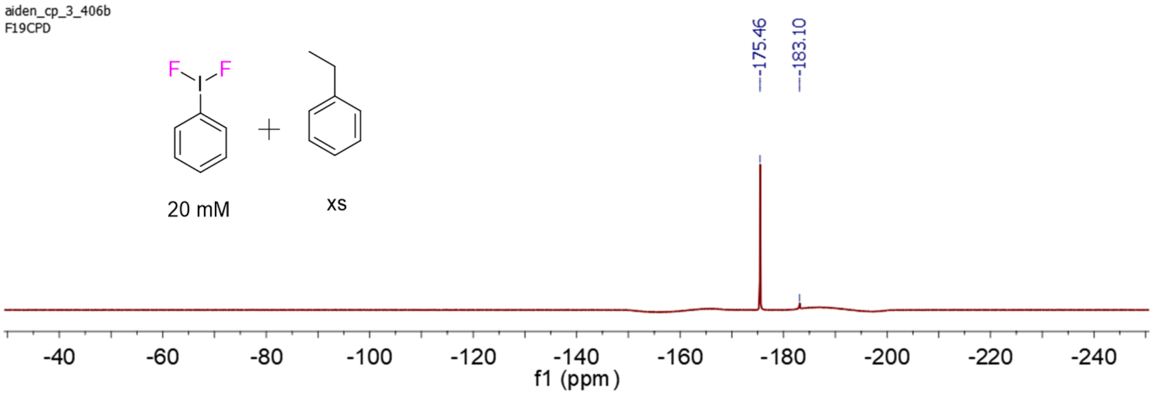

Figure S19. ^{19}F NMR of a reaction mixture containing ethylbenzene (500 mM) and $PhIF_2$ (10 mM) in CD_3CN. The signal at δ = -175 ppm corresponds to unreacted $PhIF_2$.

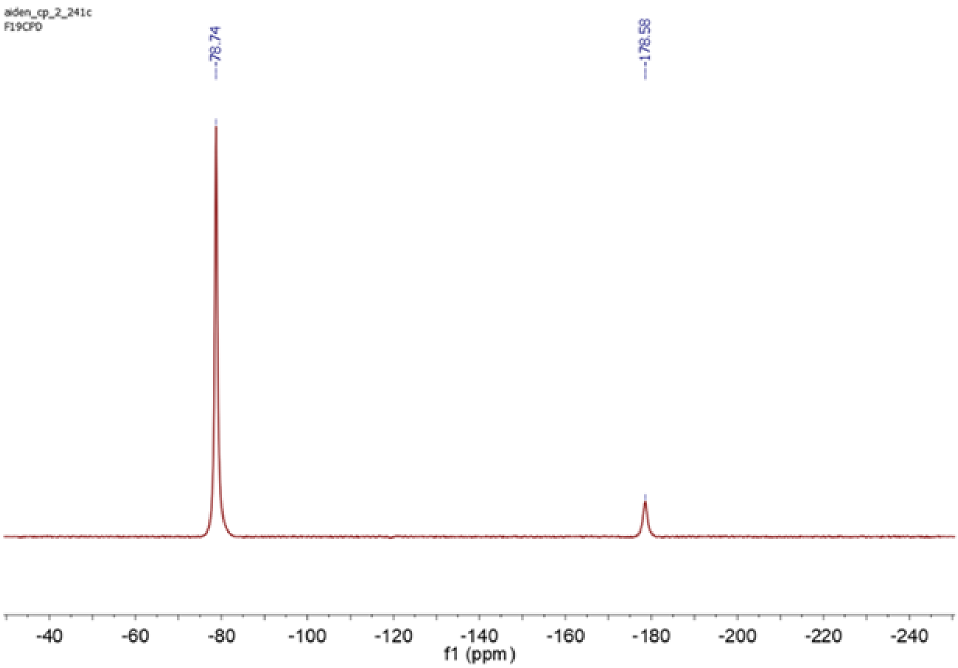

Figure S20. ^{19}F NMR of a reaction mixture containing **1** (5 mM), DHA (250 mM), and $PhIF_2$ (10 mM) in CD_3CN. The signals at δ = -78 and -175 ppm correspond to triflate and unreacted $PhIF_2$.

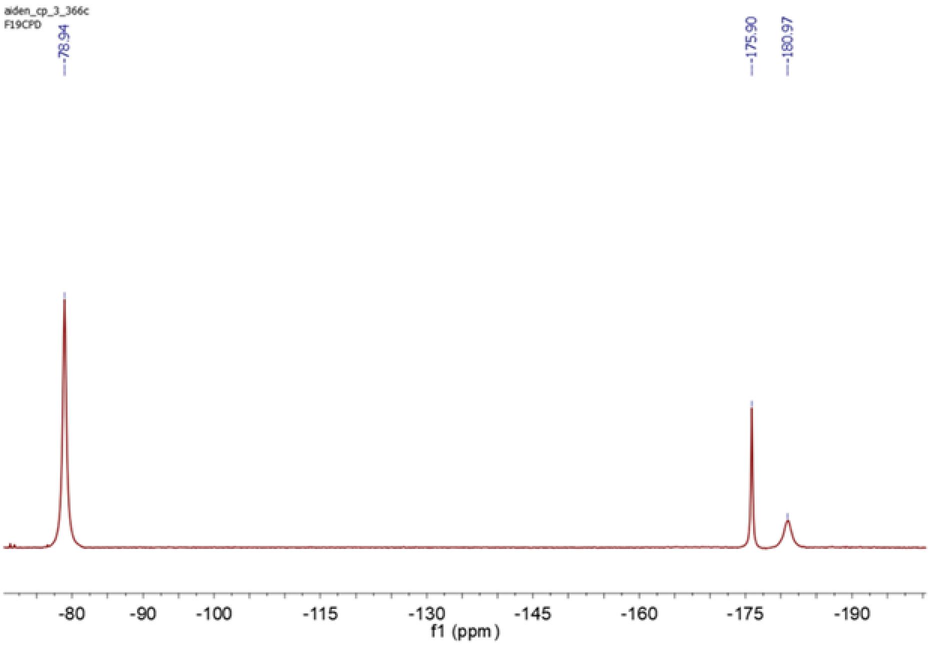

Figure S21. ^{19}F NMR of a reaction mixture containing **1** (5 mM), CHD (60 mM) and $PhIF_2$ (10 mM) in CD_3CN. The signals at δ = -78, -178, and -181 ppm corresponds to the triflate counter anion, unreacted difluoroiodobenzene and HF in acetonitrile respectively.

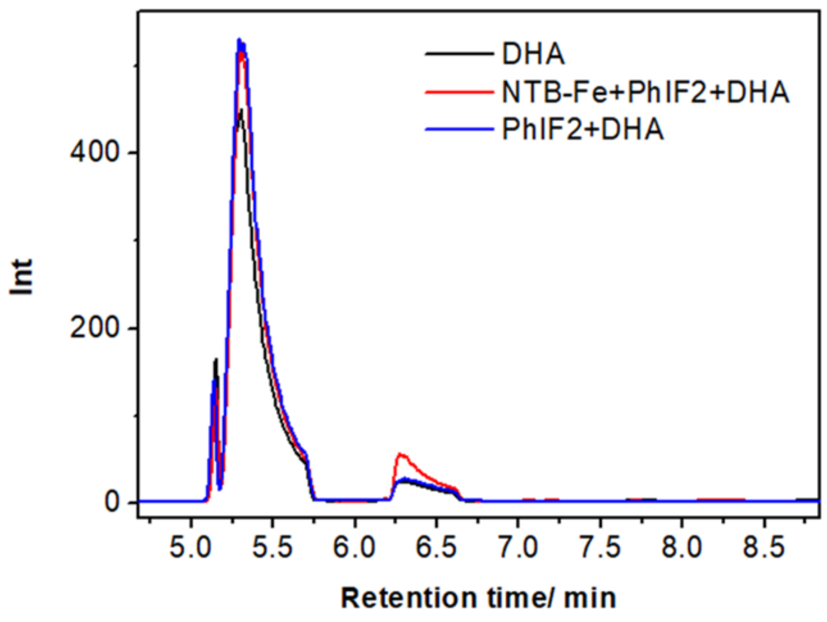

Figure S22. Gas chromatogram of a reaction mixture containing **1** (0.125 mM), DHA (50 mM) and $PhIF_2$ (0.2 mM) in CH_3CN. Quantification of anthracene was calculated to be 80% with respect to [**1**]. The control reaction of $PhIF_2$ and DHA did not contribute to formation of anymore anthracene than that was already present in the starting DHA sample.

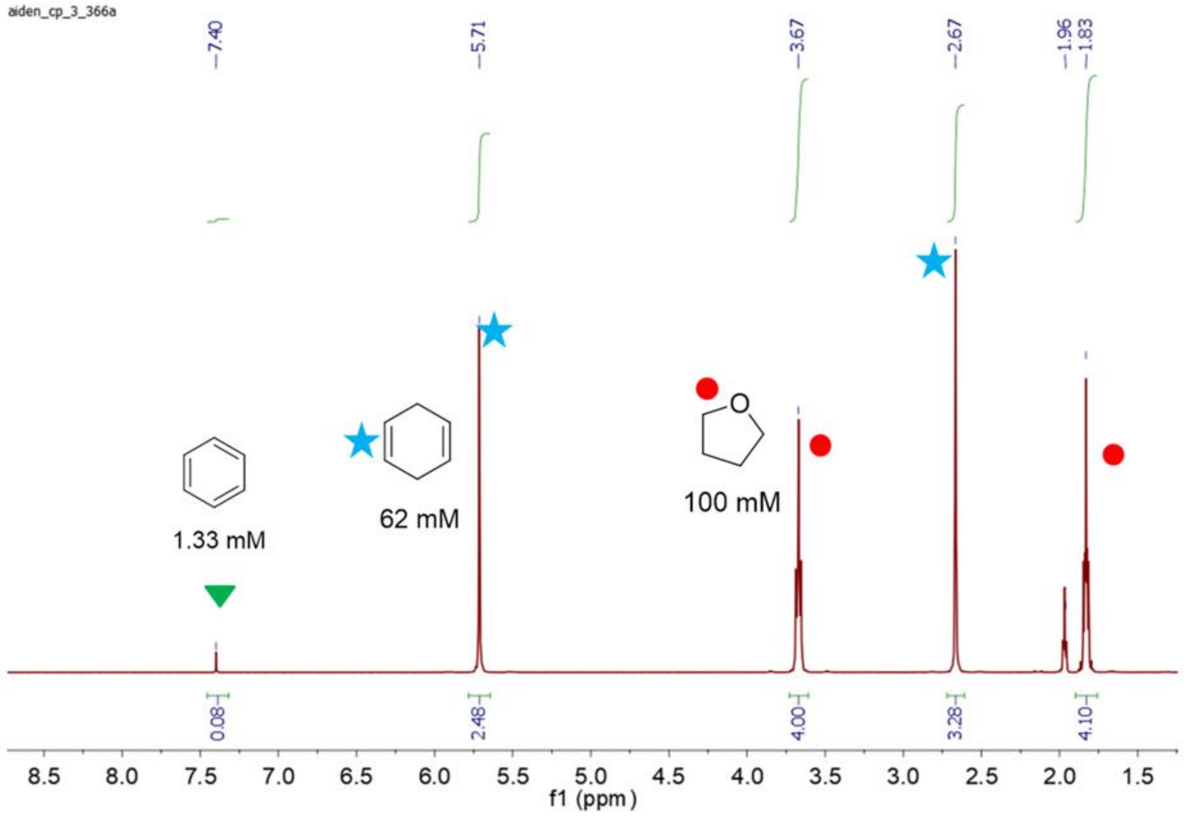

Figure S23. ^{1}H NMR of CHD (62 mM) prior to reaction in CD_3CN. THF (red dots) was used as an internal standard to quantify benzene (green triangle) already present in CHD (blue stars).

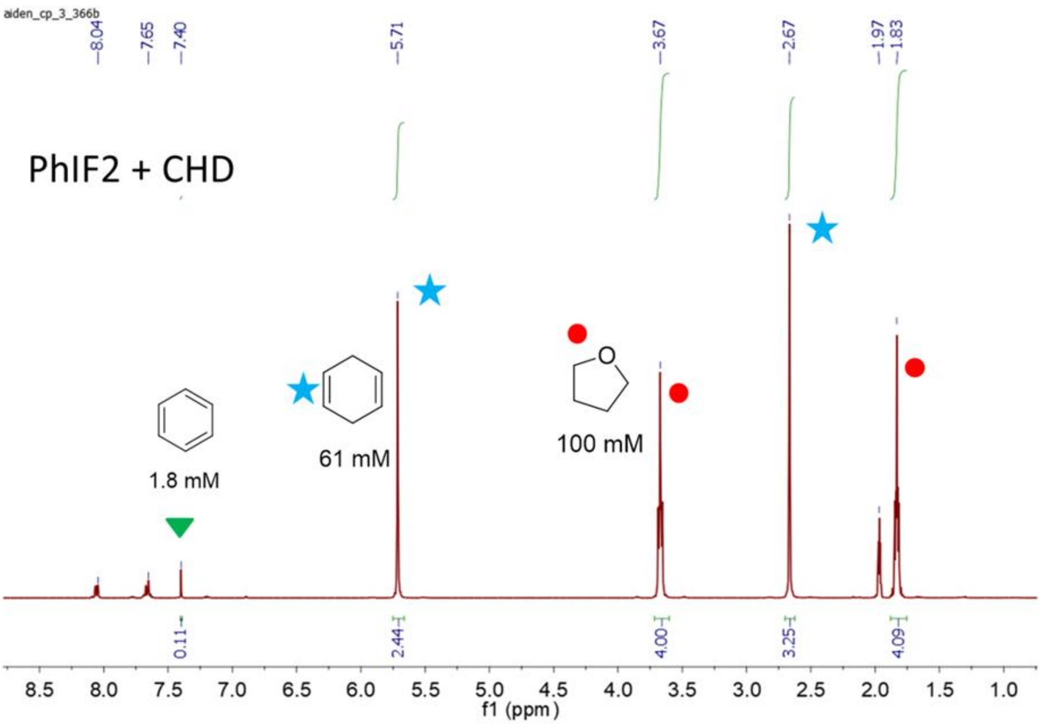

Figure S24. ^{1}H NMR of a reaction mixture containing **1** (5 mM), $PhIF_2$ (10 mM), and CHD (62 mM) in CD_3CN. THF (red dots) was used as an internal standard to quantify the formation of benzene (green triangle) from CHD (blue stars). Comparison with Figure S23, suggests that negligible benzene formation in the presence of $PhIF_2$.

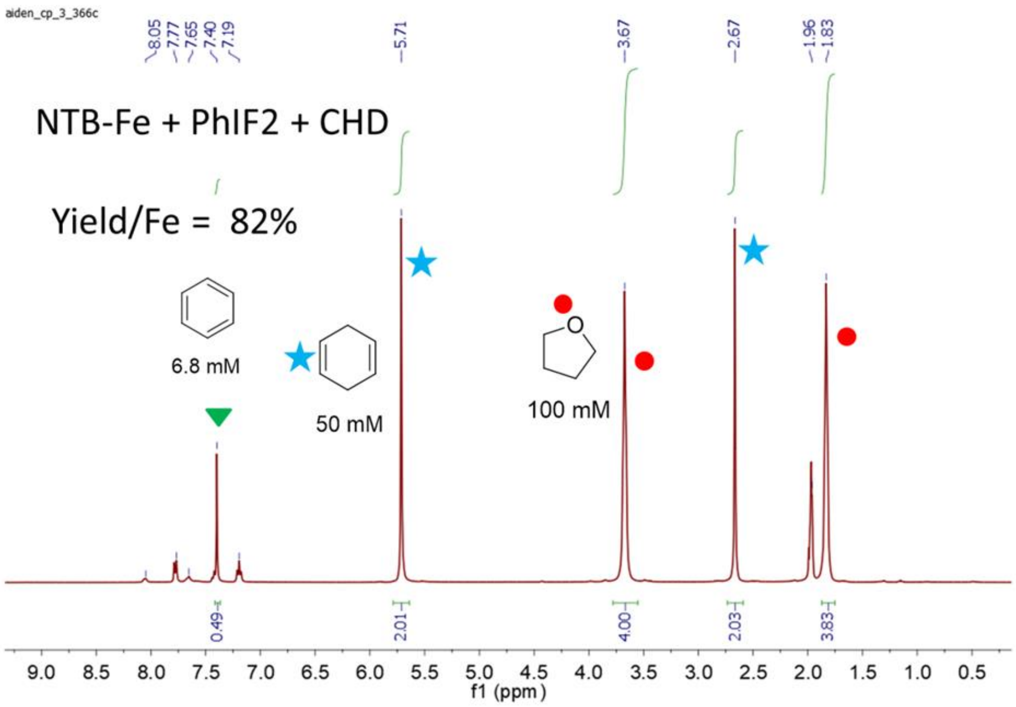

Figure S25. ^{1}H NMR of a reaction mixture containing t **1** (5 mM), $PhIF_2$ (10 mM), and CHD (62 mM) in CD_3CN. THF (red dots) was used as an internal standard to quantify the formation of benzene (green triangle) from CHD (blue stars). After subtraction of benzene concentration (1.33 mM) from the blank CHD, the yield of benzene per Fe atom is 82%. The new signals at δ = 7.19 and 7.77 ppm belong to the iodobenzene formed from $PhIF_2$.

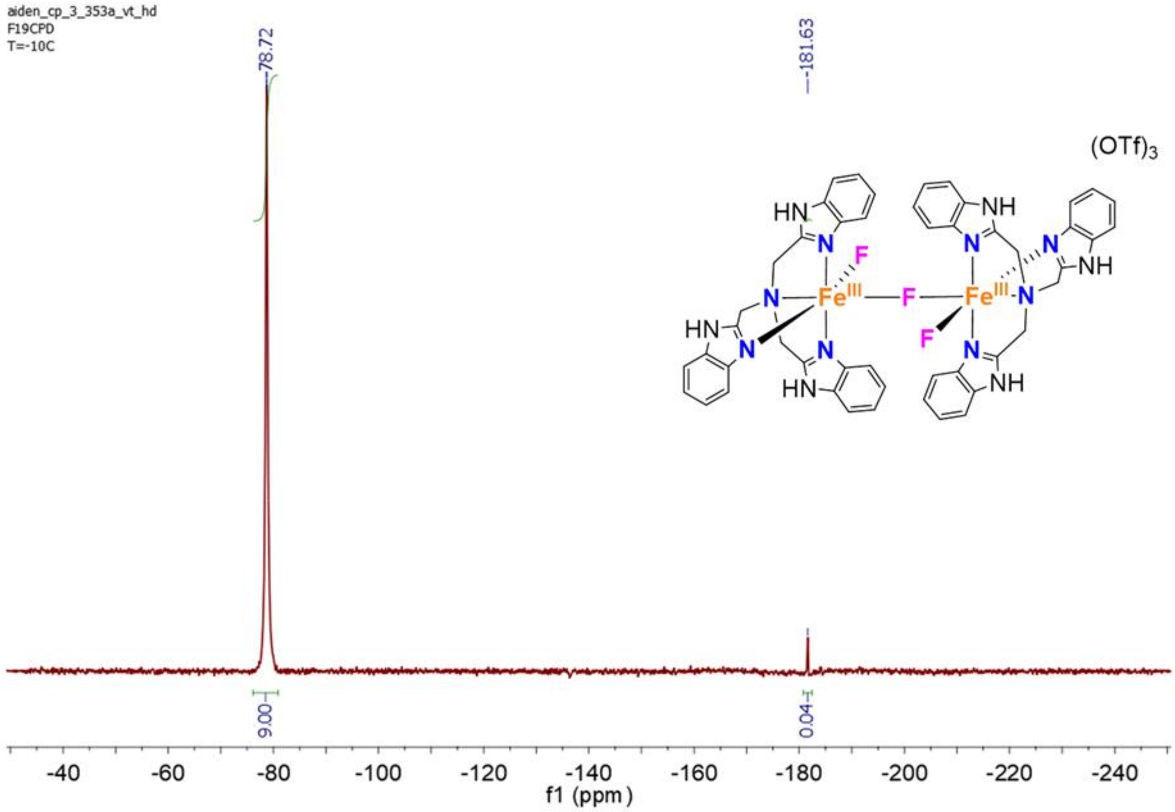

Figure S26. ^{19}F NMR of **2** (10 mM in CD_3CN).

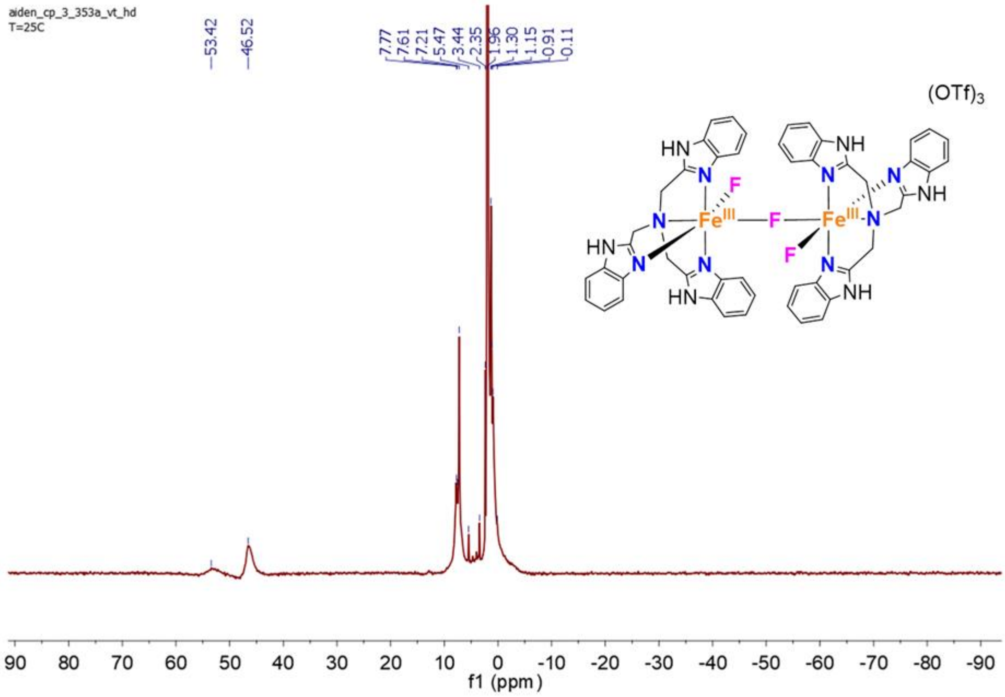

Figure S27. ^{1}H NMR of **2** (10 mM in CD_3CN).

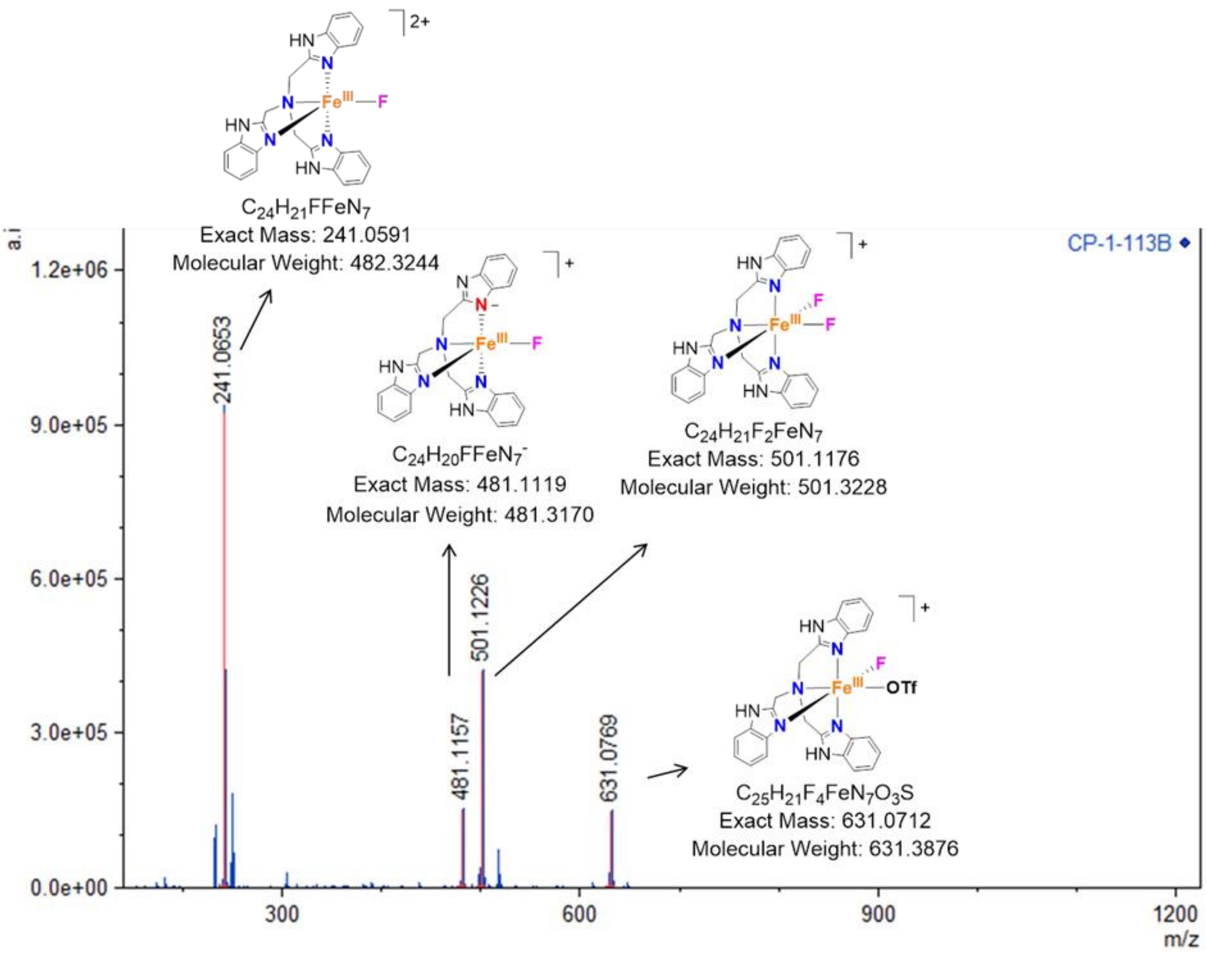

Figure S28. Full ESI-MS of **2.** The blue traces are the experimental signals and the red ones are the calculated ones.

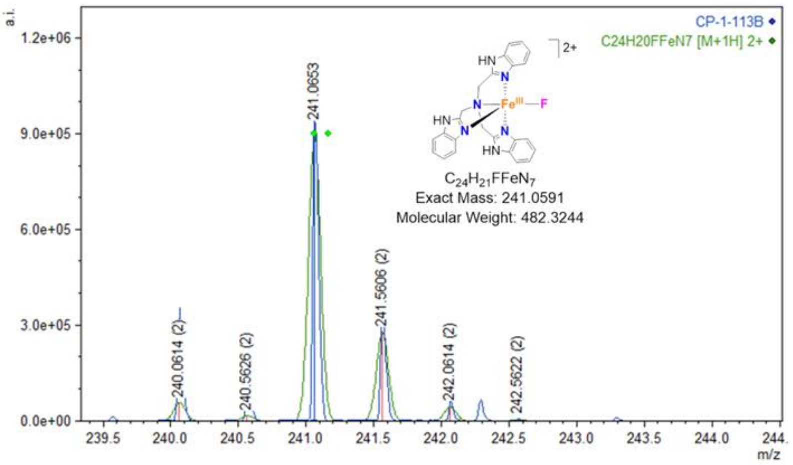

Figure S29. Isotopic mass distribution pattern for the signal m/z = 241.06. The blue and green traces correspond to the experimental and simulated m/z values respectively.

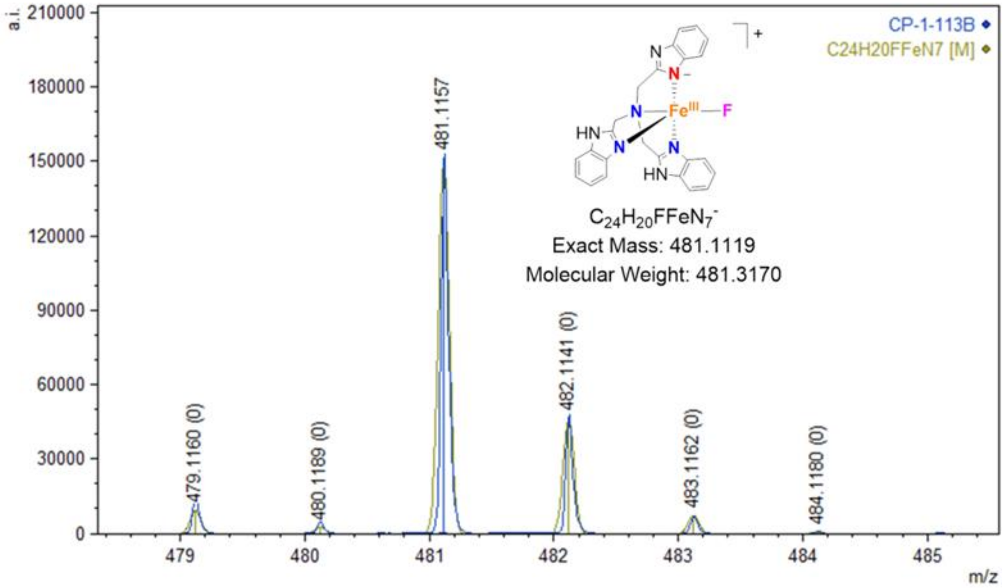

Figure S30. Isotopic mass distribution pattern for the signal m/z = 481.11. The blue and brown traces correspond to the experimental and simulated m/z values respectively.

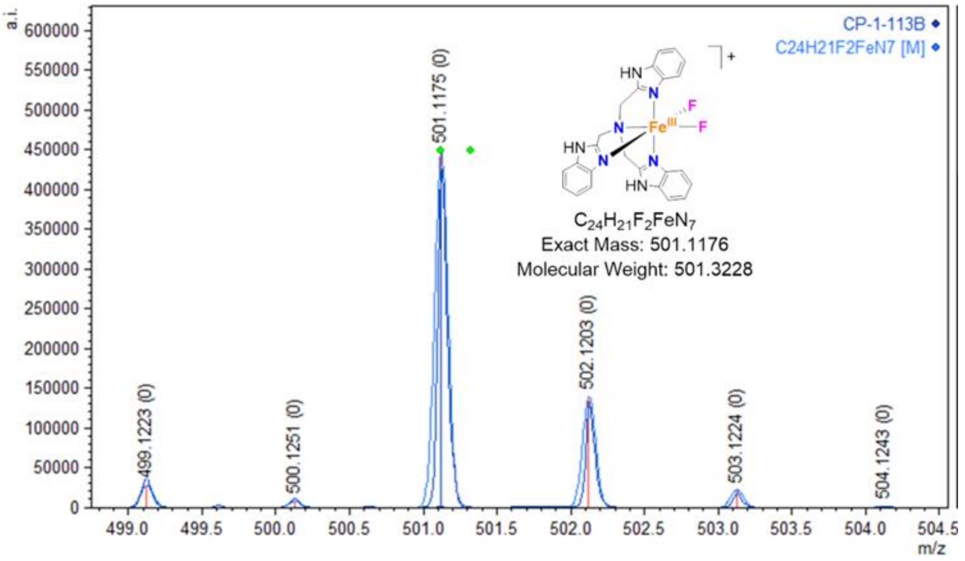

Figure S31. Isotopic mass distribution pattern for the signal m/z = 501.11. The blue and sky blue traces correspond to the experimental and simulated m/z values respectively.

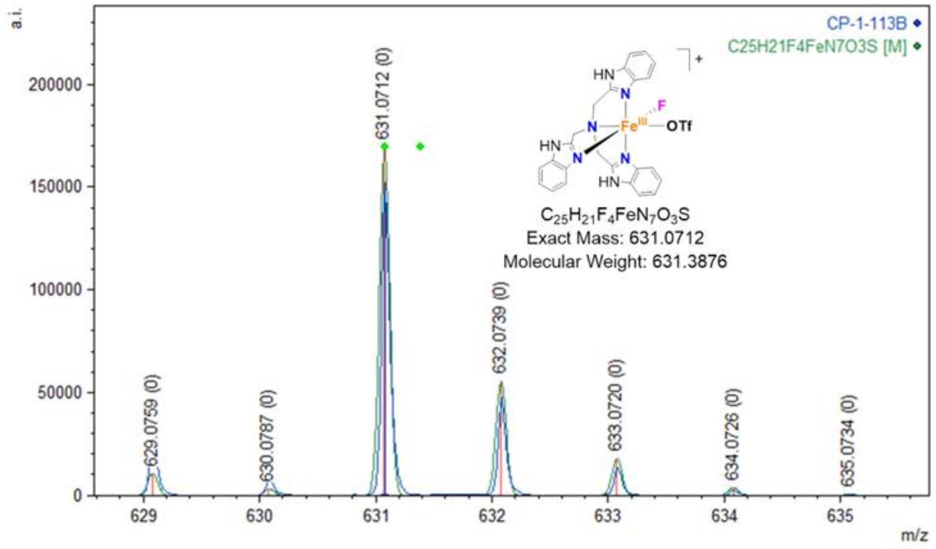

Figure S32. Isotopic mass distribution pattern for the signal m/z = 631.07. The blue and green traces correspond to the experimental and simulated m/z values respectively.

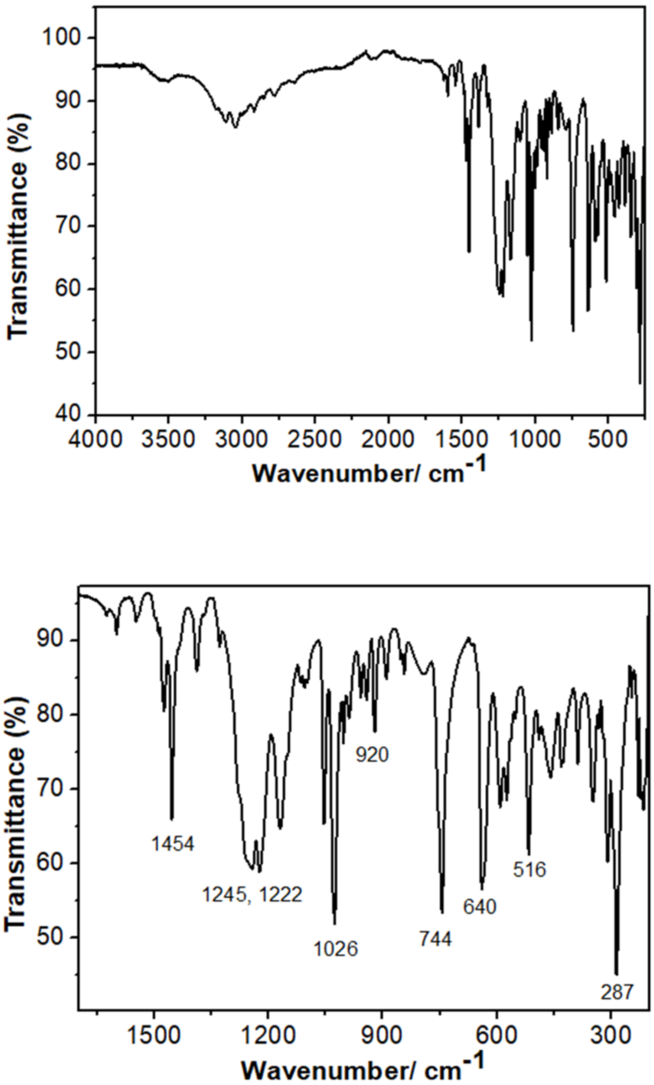

Figure S33. Top: Full FT-IR spectrum of powdered crystalline compound **2.** Bottom: FT-IR of **2** showing the region between 1600 to 250 cm^{-1}.

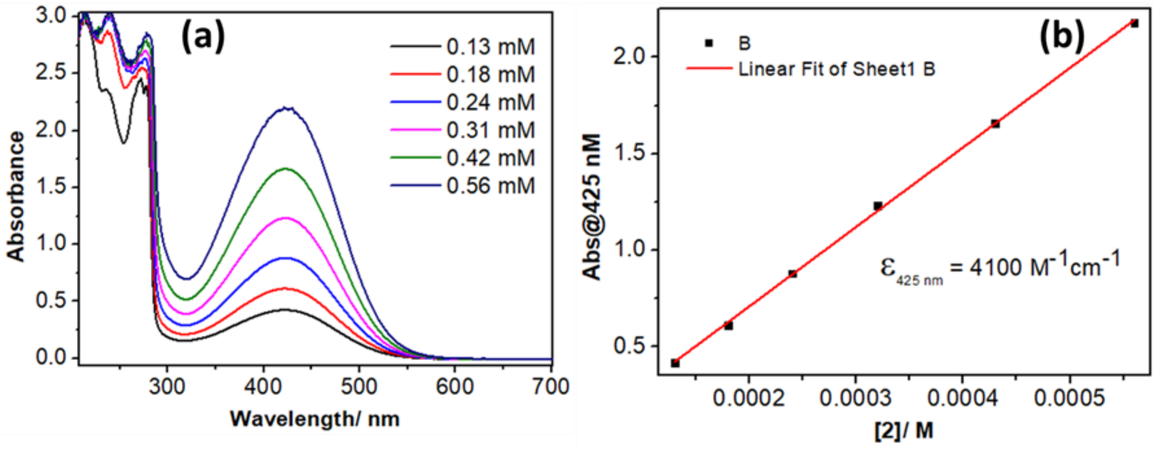

Figure S34. (a) UV-vis spectrum of **2** (different concentrations in CH_3CN). (b) Plot of absorbance at λ = 425 nm and concentration of **2** to calculate the molar extinction coefficient.

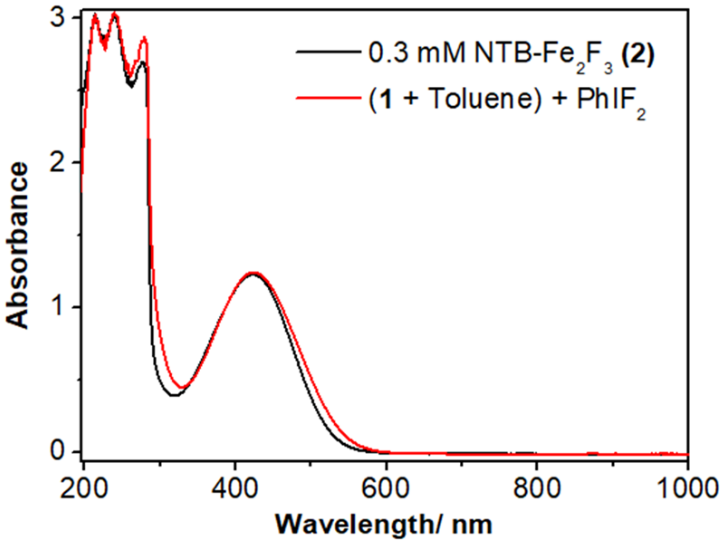

Figure S35. UV-vis spectrum of crystals of **2** dissolved in CH_3CN (black). Final spectrum of a reaction mixture containing **1**, toluene, and $PhIF_2$ in CH_3CN (red).

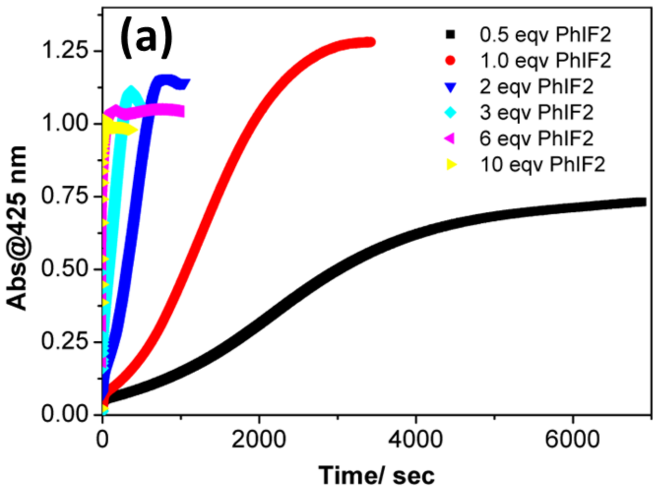

Figure S36. Time traces for the formation of **2** from the reaction of **1** and varying equivalents of $PhIF_2$.

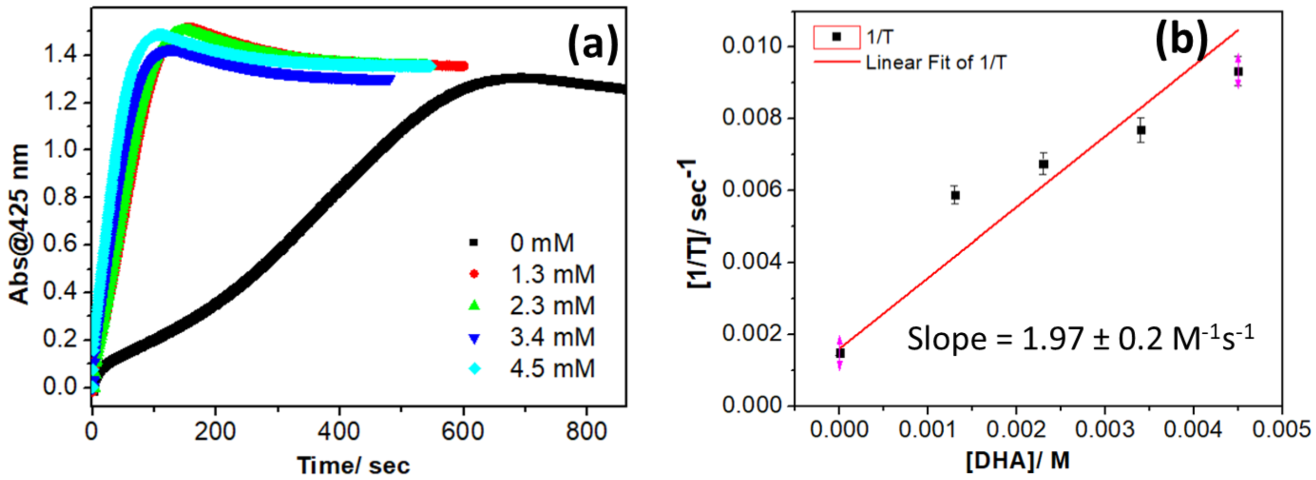

Figure S37. (a) Time traces for the formation of **2** from the reaction of **1** and $PhIF_2$ in the presence of different concentrations of DHA. (b) Plot of reciprocal of time taken for the maximum formation of **2** vs concentrations of DHA and the linear fitting to obtain the second order reaction rate constant.

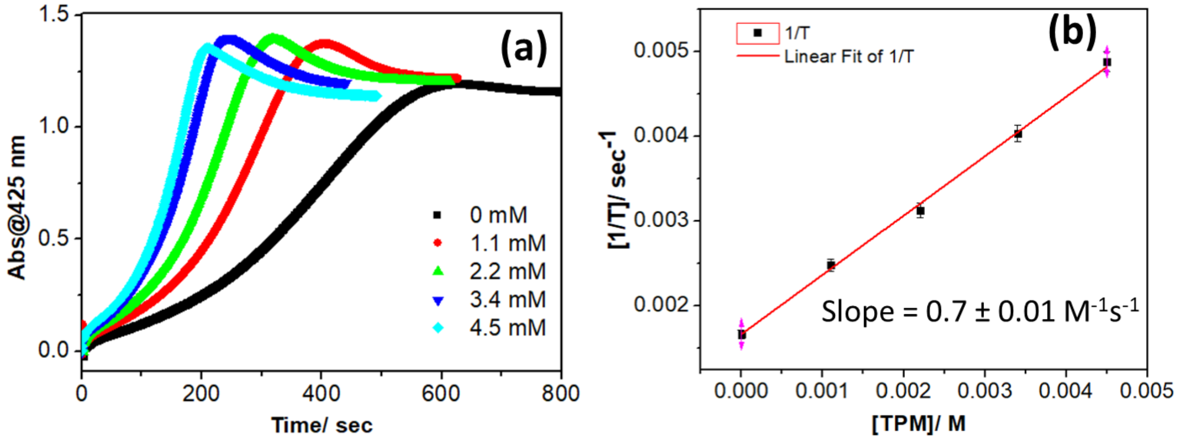

Figure S38. (a) Time traces for the formation of **2** from the reaction of **1** and $PhIF_2$ in the presence of different concentrations of triphenylmethane. (b) Plot of reciprocal of time taken for the maximum formation of **2** vs concentrations of triphenylmethane and the linear fitting to obtain the second order reaction rate constant.

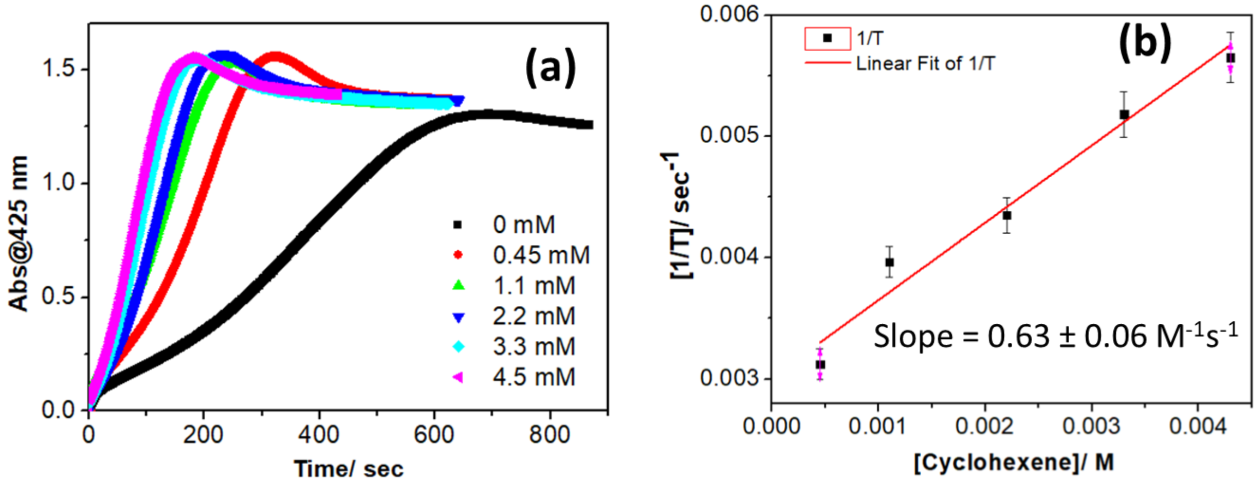

Figure S39. (a) Time traces for the formation of **2** from the reaction of **1** and $PhIF_2$ in the presence of different concentrations of cyclohexene. (b) Plot of reciprocal of time taken for the maximum formation of **2** vs concentrations of cyclohexene and the linear fitting to obtain the second order reaction rate constant.

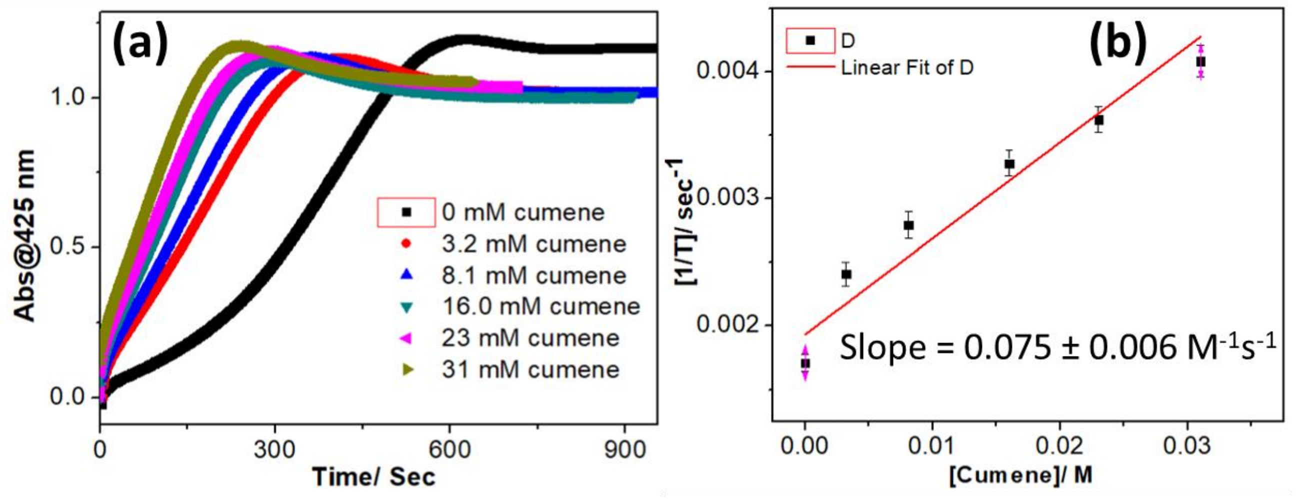

Figure S40. (a) Time traces for the formation of **2** from the reaction of **1** and $PhIF_2$ in the presence of different concentrations of cumene. (b) Plot of reciprocal of time taken for the maximum formation of **2** vs concentrations of cumene and the linear fitting to obtain the second order reaction rate constant.

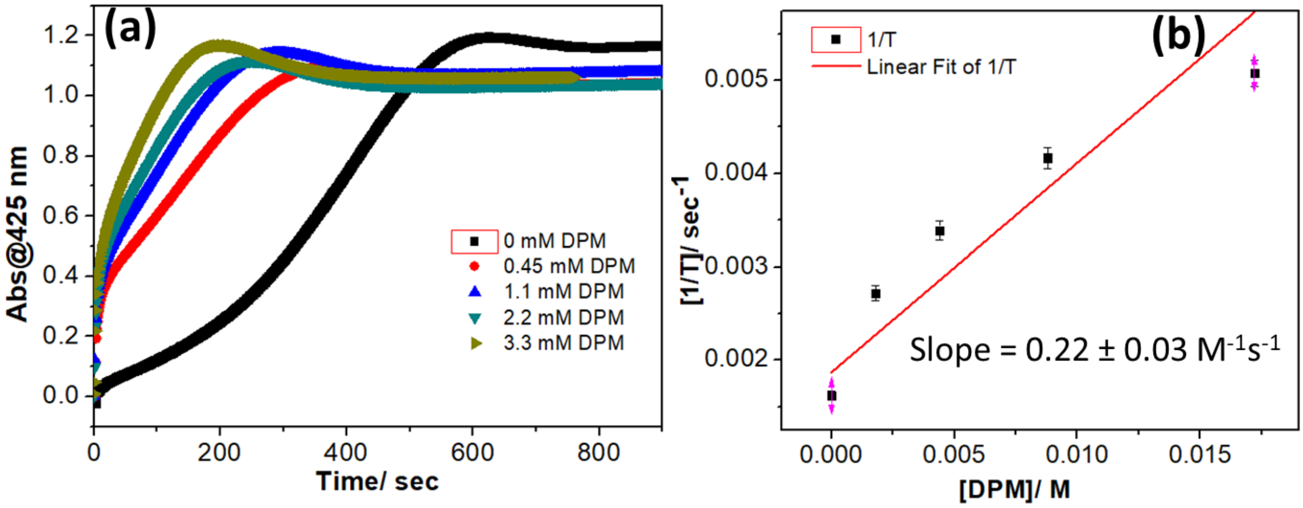

Figure S41. (a) Time traces for the formation of **2** from the reaction of **1** and $PhIF_2$ in the presence of different concentrations of diphenylmethane. (b) Plot of reciprocal of time taken for the maximum formation of **2** vs concentrations of diphenylmethane and the linear fitting to obtain the second order reaction rate constant.

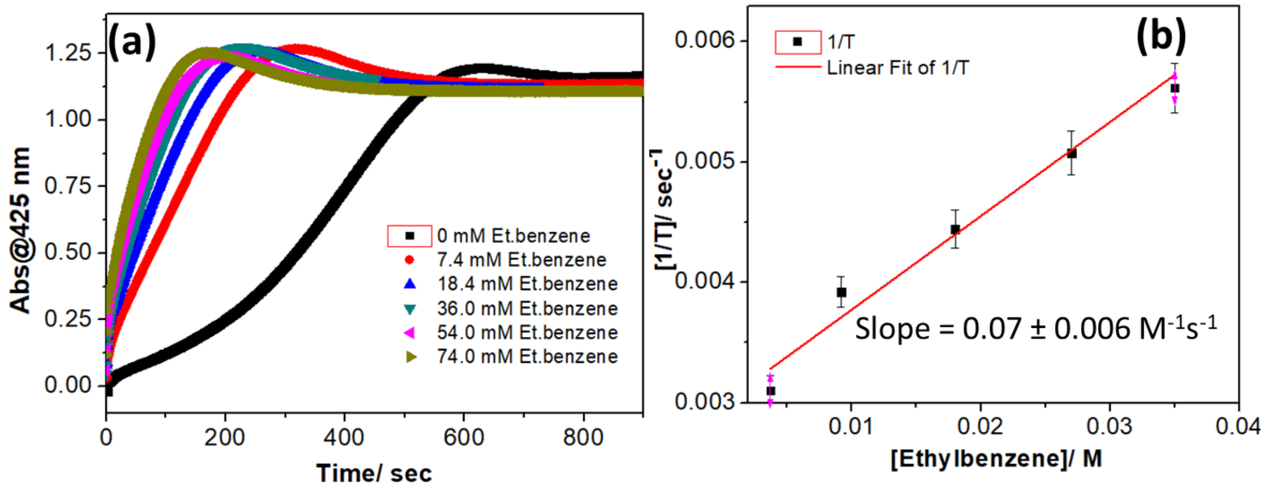

Figure S42. (a) Time traces for the formation of **2** from the reaction of **1** and $PhIF_2$ in the presence of different concentrations of ethylbenzene. (b) Plot of reciprocal of time taken for the maximum formation of **2** vs concentrations of ethylbenzene and the linear fitting to obtain the second order reaction rate constant.

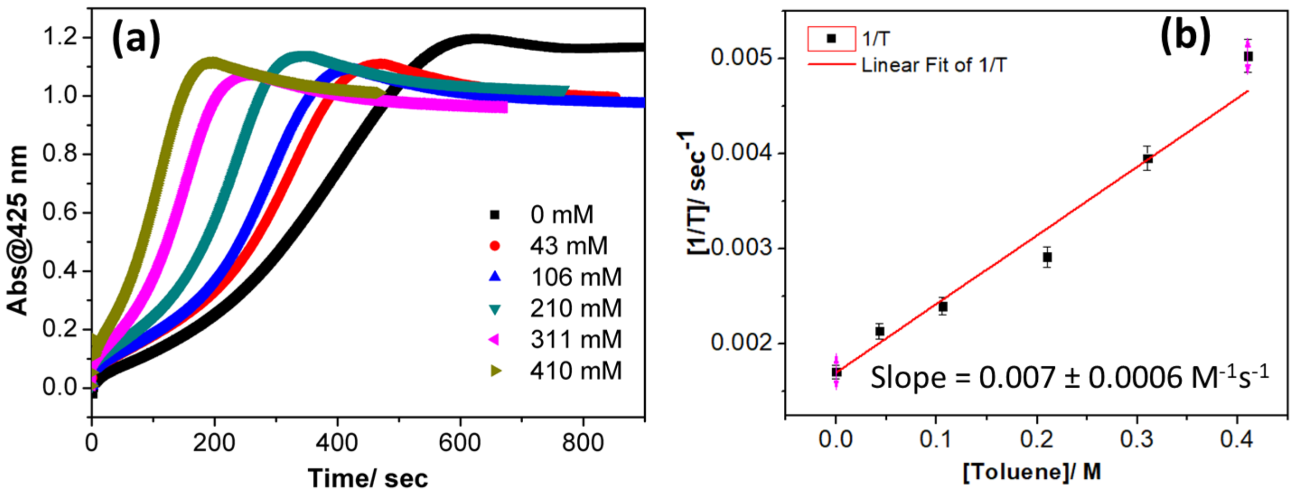

Figure S43. (a) Time traces for the formation of **2** from the reaction of **1** and $PhIF_2$ in the presence of different concentrations of toluene. (b) Plot of reciprocal of time taken for the maximum formation of **2** vs concentrations of toluene and the linear fitting to obtain the second order reaction rate constant.

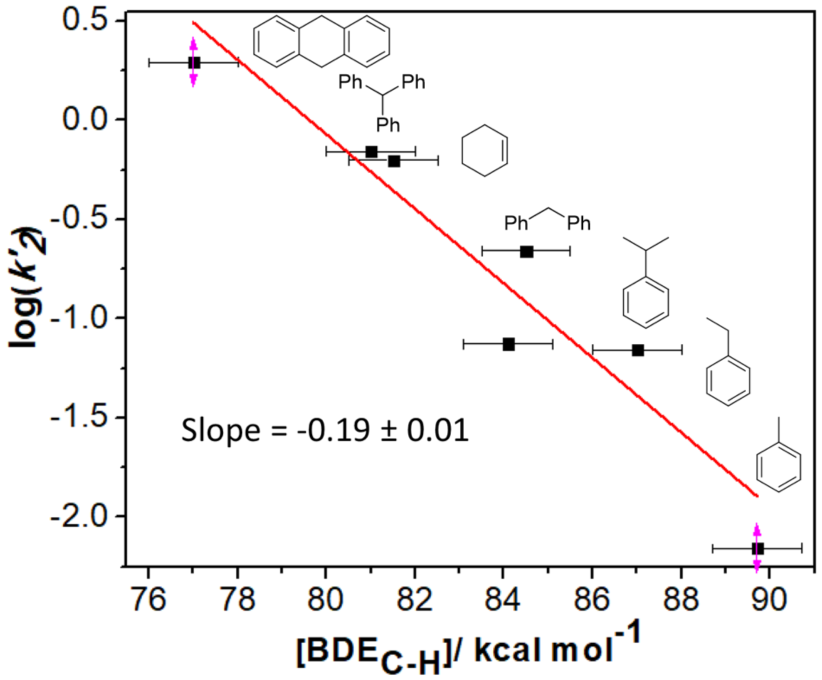

Figure S44. (a) Plot of log(k_2) vs. BDE$_{C–H}$.[6, 7] As bond dissociation free energy (BDFE$_{C–H}$) for all the substrates in CH_3CN are not available, we have plotted the BDE$_{C–H}$ instead, which acts as a reliable surrogate.[6, 7]

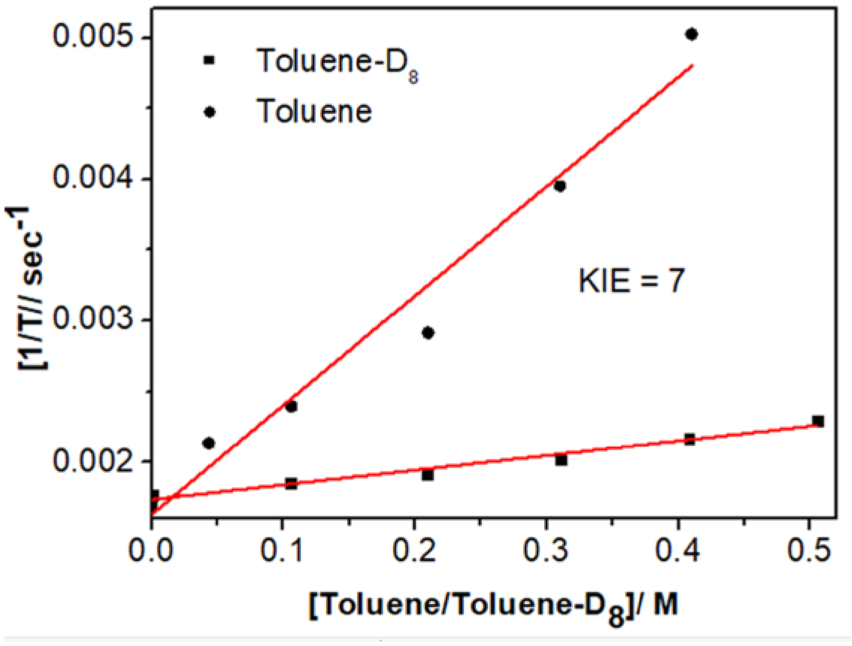

Figure 45. Plots of k_{obs} versus [H/D-toluene] determined for the reactions between **1**, $PhIF_2$, and toluene (black dots), toluene-D_8 (black squares) resulting in the formation of **2**.

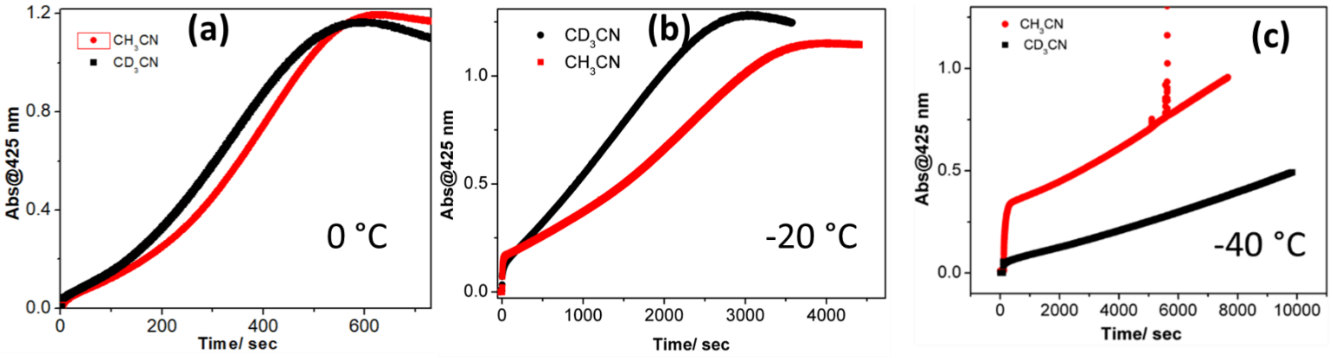

Figure S46. Time traces for the formation of **2** from the reaction of **1** and $PhIF_2$ in CH_3CN (red traces) and CD_3CN (black traces) at different temperatures (a) 0 °C, b) -20 °C and c) -40 °C)).

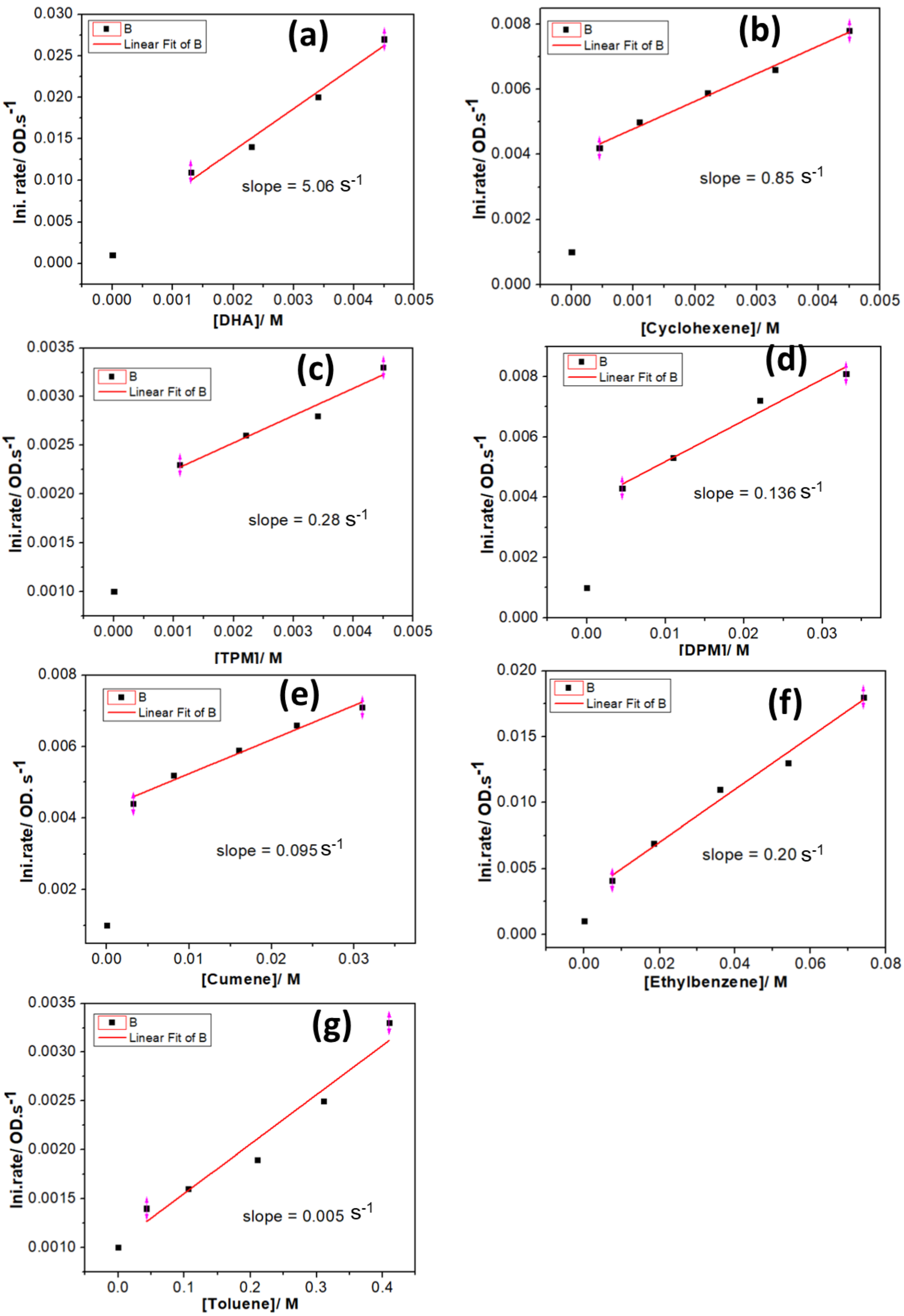

Figure S47. Plot of initial rate (for initial sixty seconds) versus substrate concentration to obtain rate constants (k'_1). a) DHA, b) cyclohexene, c) TPM, d) DPM, e) cumene, f) ethylbenzene and g) toluene.

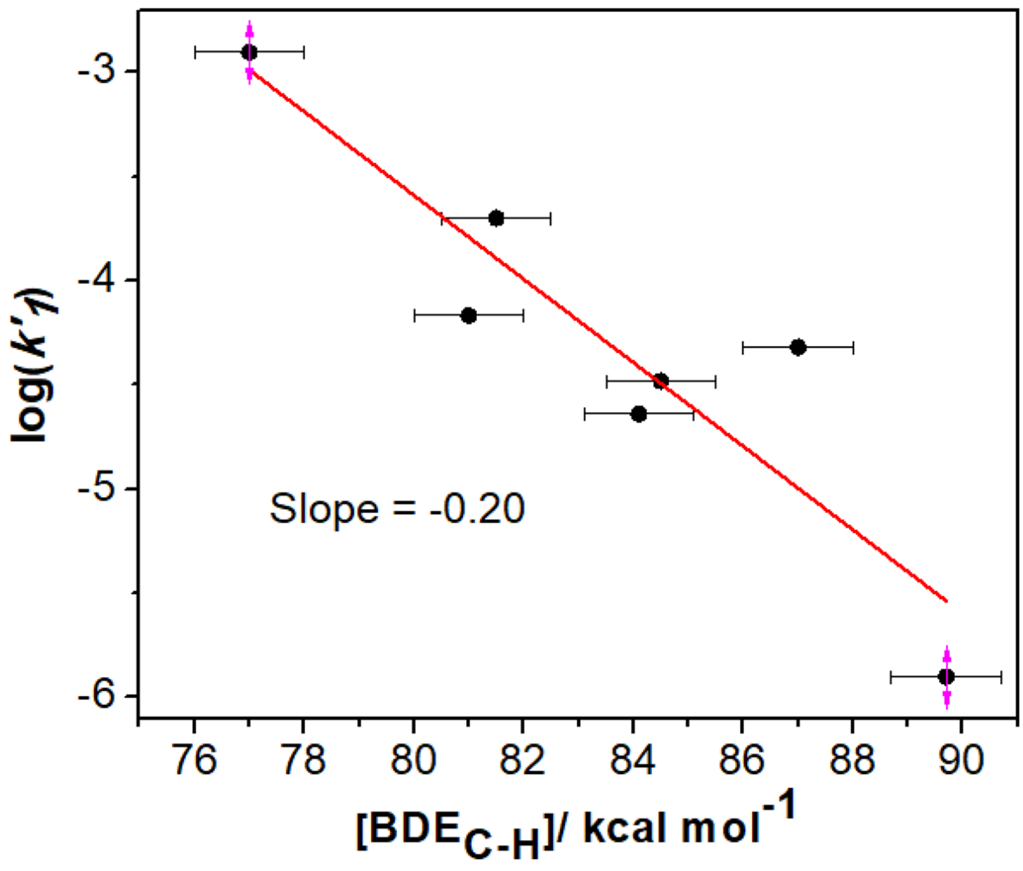

Figure S48. Plot of log(*k'*$_1$) vs. BDE$_{C–H}$.[6, 7] (*k'*$_1$ are taken from figure S47) As bond dissociation free energy (BDFE$_{C–H}$) for all the substrates in CH_3CN are not available, we have plotted the BDE$_{C–H}$ instead, which acts as a reliable surrogate.[6, 7]

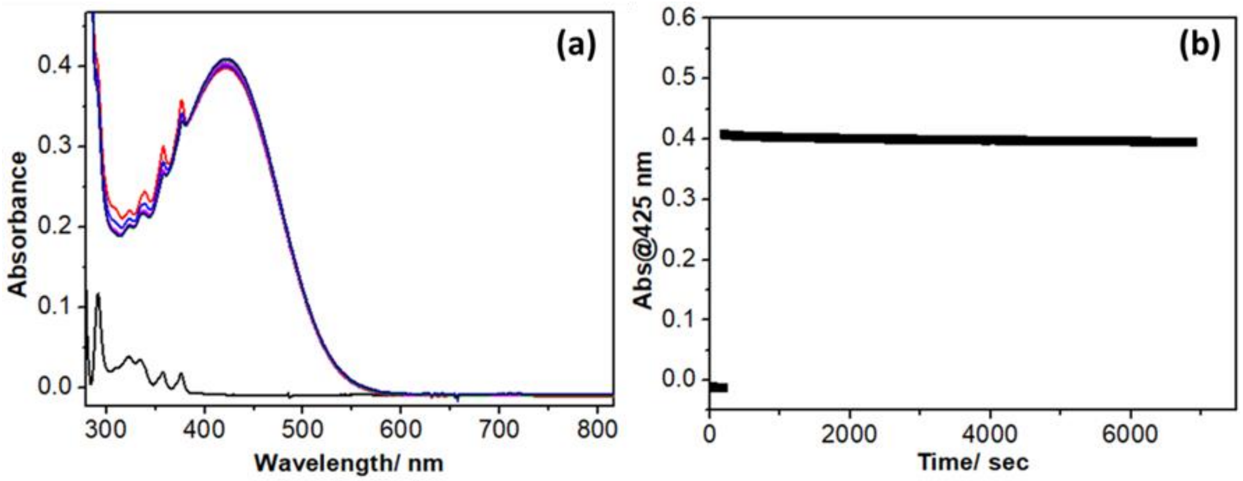

Figure S49. (a) UV-vis spectral change upon addition of **2** (0.1 mM) to a 250 mM solution of DHA in CH_3CN. The black trace corresponds to the initial DHA and the colored traces for the mixture after addition of **2**. (b) Time trace for said reaction monitored at λ = 425 nm. There was negligible change in absorbance intensity indicating no reaction with DHA.

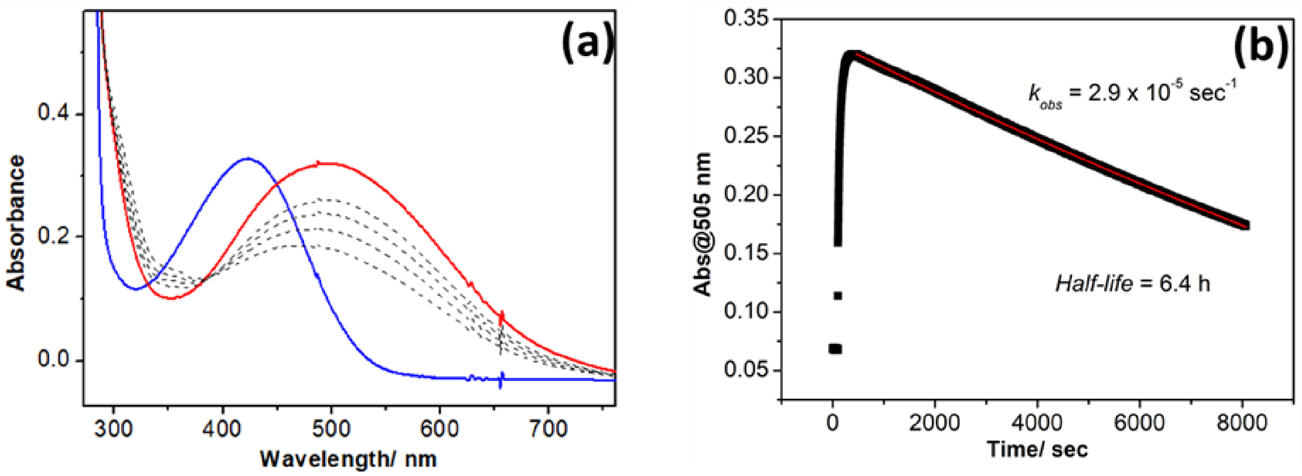

Figure S50. (a) UV-vis spectral change upon addition of $Sc(OTf)_3$ (4 equiv.) to a solution of **2** (0.1 mM, blue trace) in CH_3CN at 0 °C. (b) Time trace at λ = 505 nm for the formation and self-decay of the species **3** at 505 nm.

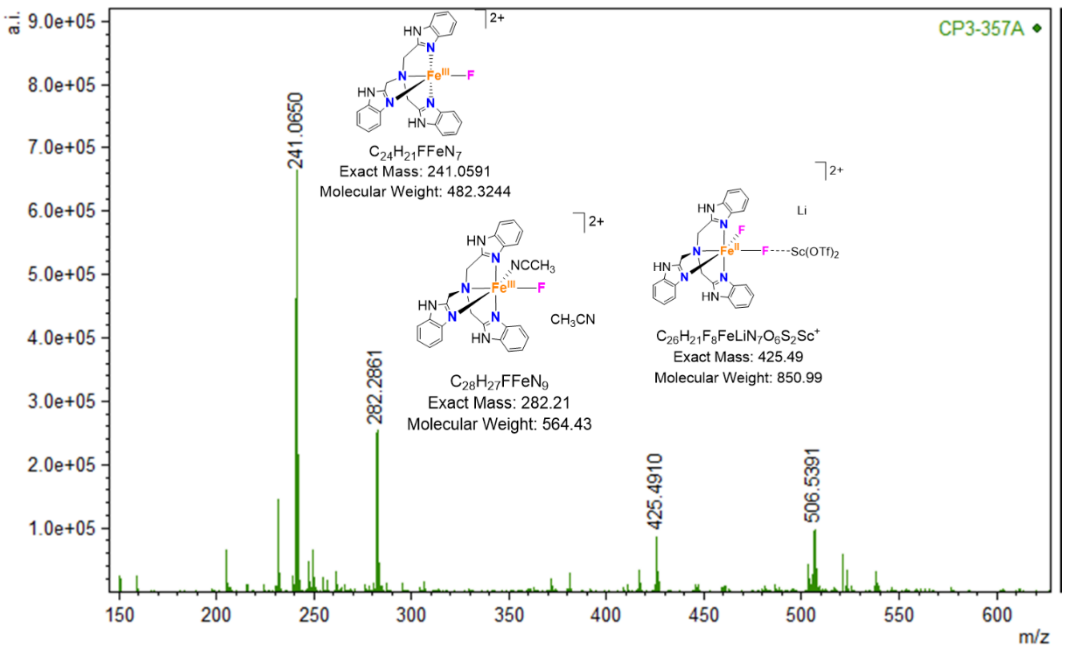

Figure S51. ESI-MS of **3** in CH_3CN.

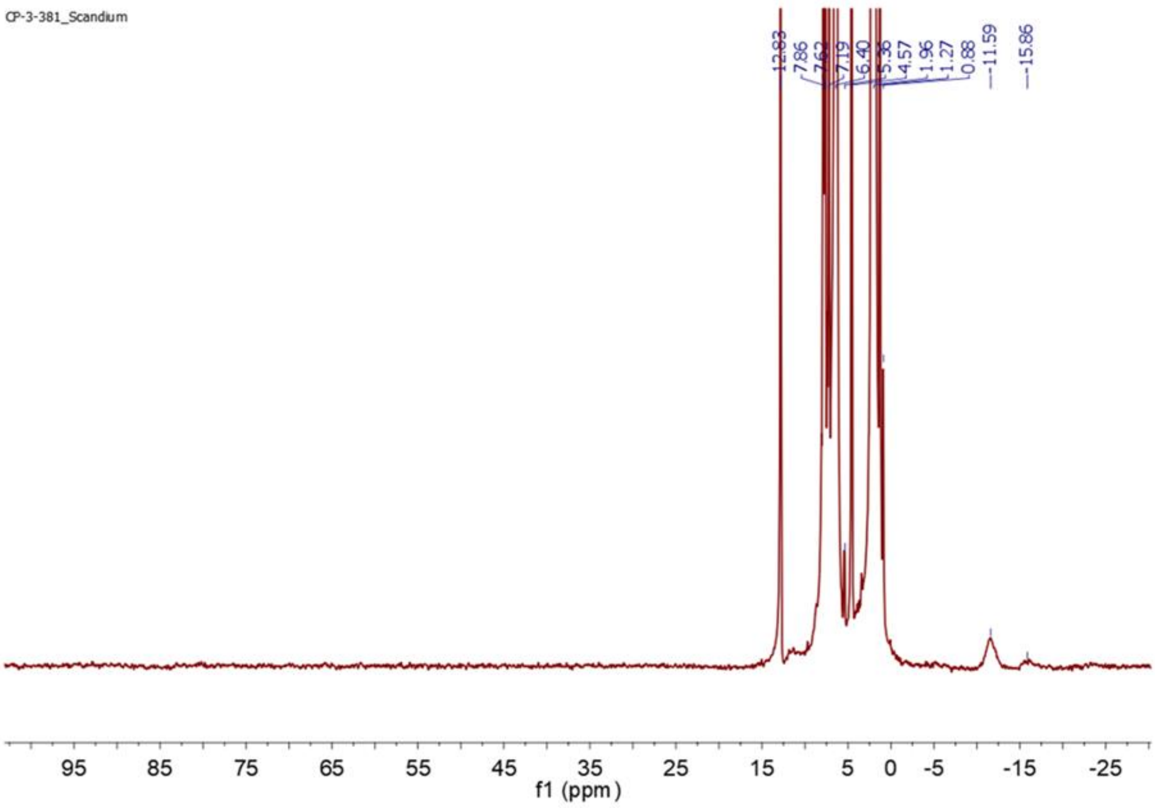

Figure S52. ^{1}H NMR of **3** in CD_3CN.

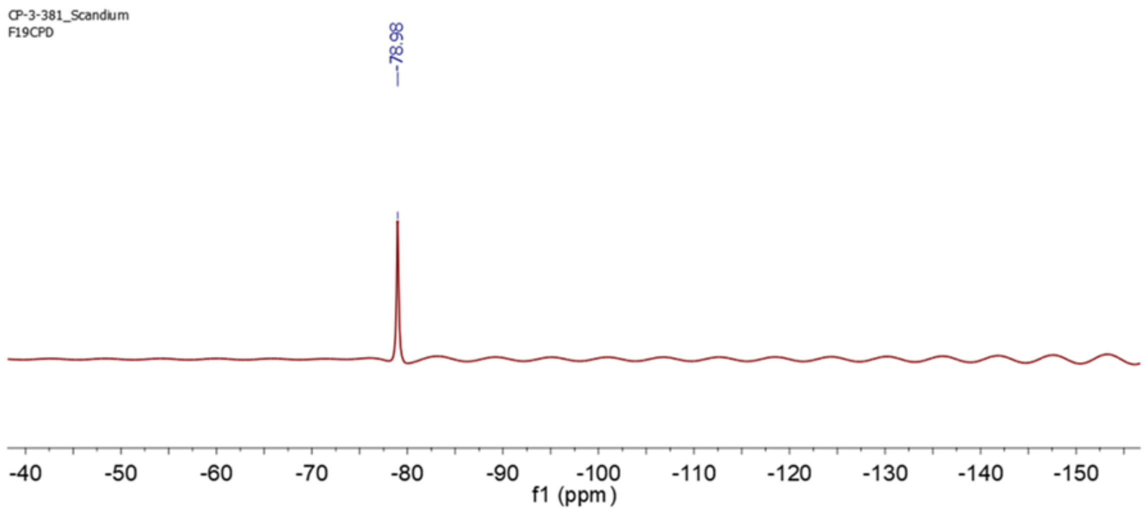

Figure S53. ^{19}F NMR of **3** in CD_3CN.

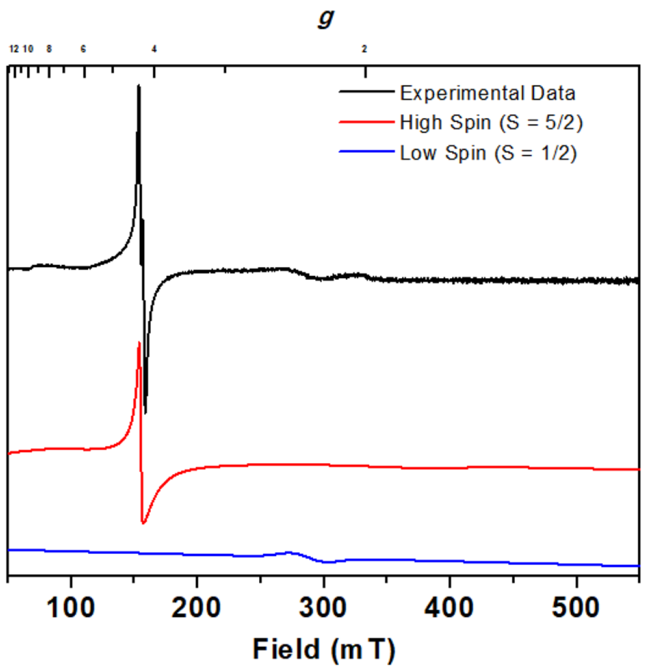

Figure S54: X-band EPR spectrum of **3** (5 mM, black trace) in 50% DMF:CH_3CN at -78 K measured at 77 K, 9.29 GHz, 2.01 mW power with 0.3 mT field modulation amplitude. Red trace corresponds to the simulated spectrum.

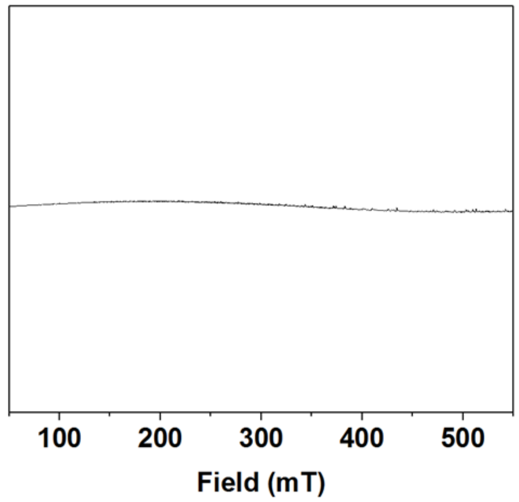

Figure S55. Perpendicular X-band EPR of **2** (5 mM frozen in CH_3CN) recorded at 77 K, 9.302 GHz, 2.01 mW and 0.3 mT field modulation..

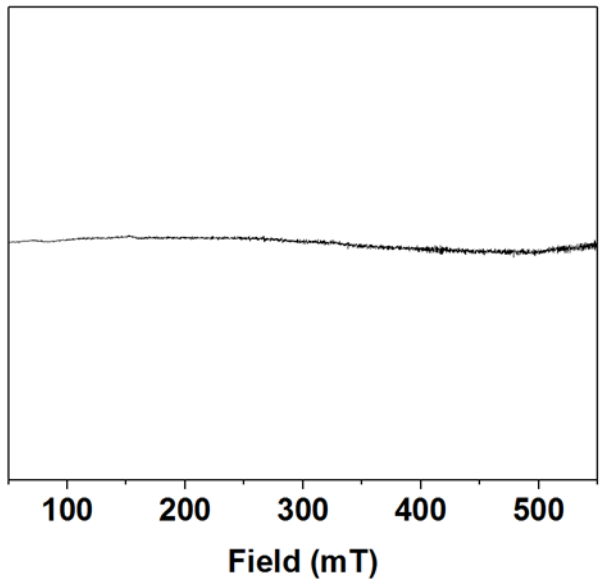

Figure S56. Perpendicular X-band EPR of **1** + $PhIF_2$ (5 mM frozen in CH_3CN) recorded at 77 K, 9.297 GHz, 2.01 mM, and 0.3 mT field modulation.

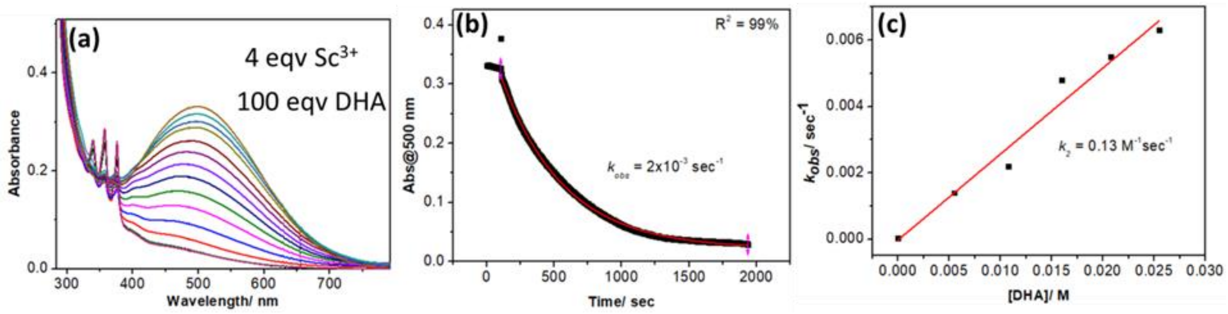

Figure S57. (a) Spectral changes observed upon reaction of **3** (0.1 mM) with DHA (100 equiv. 10 mM). (b) *Pseudo*-first order fitting to obtain the rate constants (k_{obs}). (c) Plot of k_{obs} vs [DHA] to calculate the second order reaction rate constant k_2.

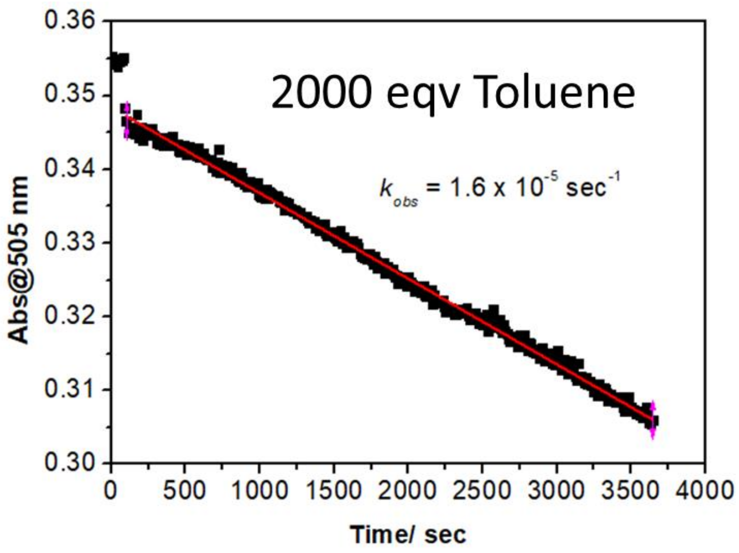

Figure S58. Time traces at λ = 505 nm for the reaction of **3** (0.1 mM) and toluene (2000 equiv.). The k_{obs} was calculated to be in the same order of magnitude to that of the self-decay of **3**. Therefore, we conclude that **3** is not capable of activating C–H bonds in toluene.

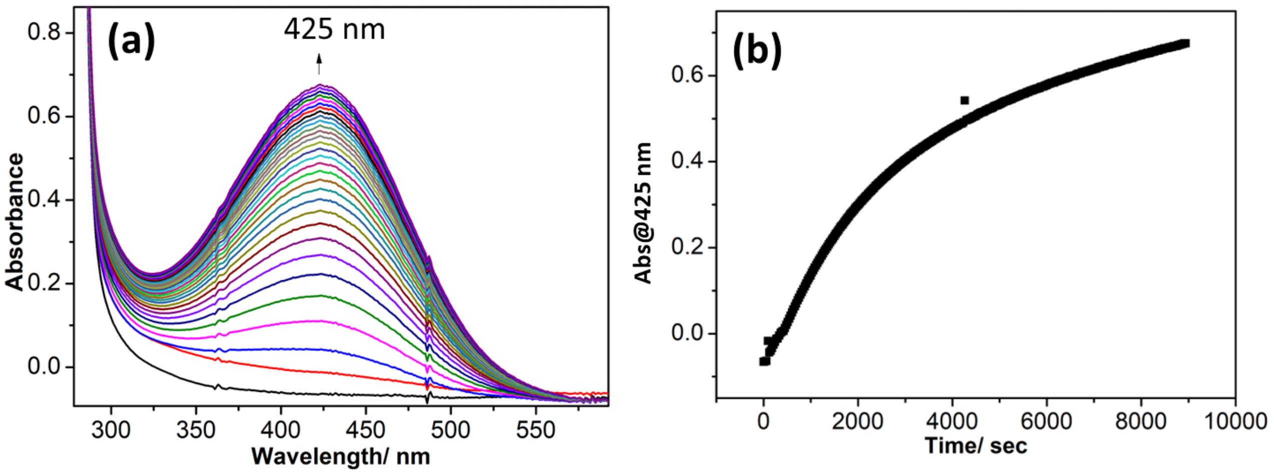

Figure S59. (a) UV-vis spectral change for a reaction of **1** (0.25 mM) and selectfluor (2 equiv.) in CH_3CN at 20 °C. (b) The time trace at λ_{max} = 425 nm showing the formation of **2**.

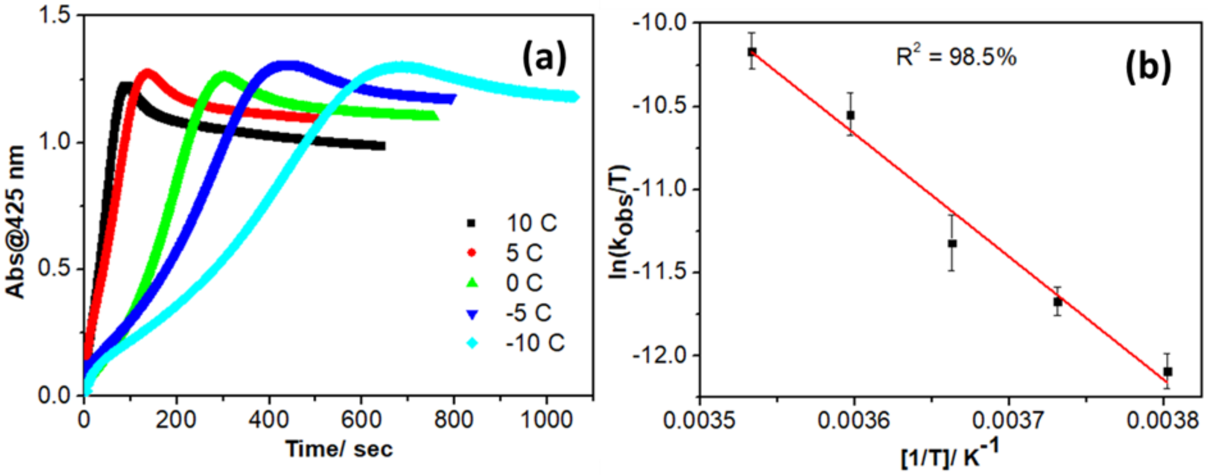

Figure S60. (a) Time traces for the reaction of **1**, toluene (400 equiv.), and $PhIF_2$ at different temperatures in CH_3CN. (b) Plot of ln(k_{obs}/T) versus 1/T for the above reactions for the calculation of activation parameters ($\Delta H^{\ddagger}$ and $\Delta S^{\ddagger}$).

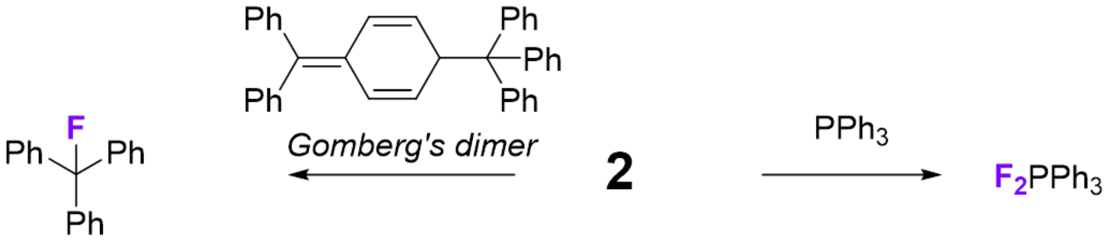

Scheme S1. Fluorine atom transfer reactivity of **2** towards Gomberg's dimer and triphenylphosphine.

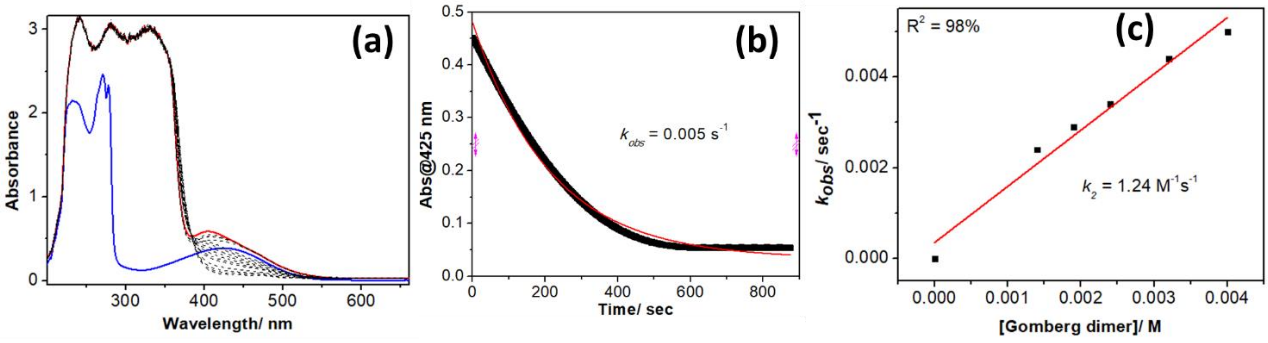

Figure S61. (a) UV-vis spectral change for a reaction of **2** (0.1 mM; blue trace) and Gomberg's (10 mM) dimer in CH_3CN at room temperature. Following addition of Gomberg's dimer to **2**, the blue trace immediately converts into the red trace and then decays (black dotted traces). (b) First order fitting for the reaction between **2** and Gomberg's dimer to obtain the k_{obs}. (c) Plot of k_{obs} vs. [Gomberg's dimer] to calculate the second order reaction rate constant.

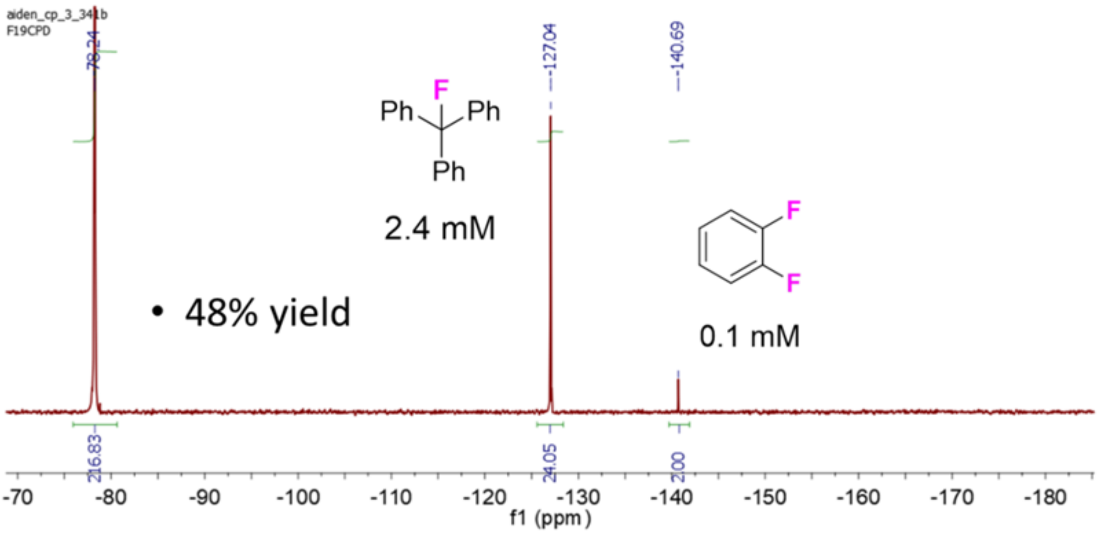

Figure S62. ^{19}F NMR for a reaction of **2** (2.5 mM) and Gomberg's dimer (25 mM) in CD_3CN at room temperature. A singlet at δ = -127 ppm corresponds to the trityl fluoride. 1,2-difluoro benzene (δ = -140 ppm) was used as an internal standard for quantification of trityl fluoride (48% yield with respect to [**2**]).

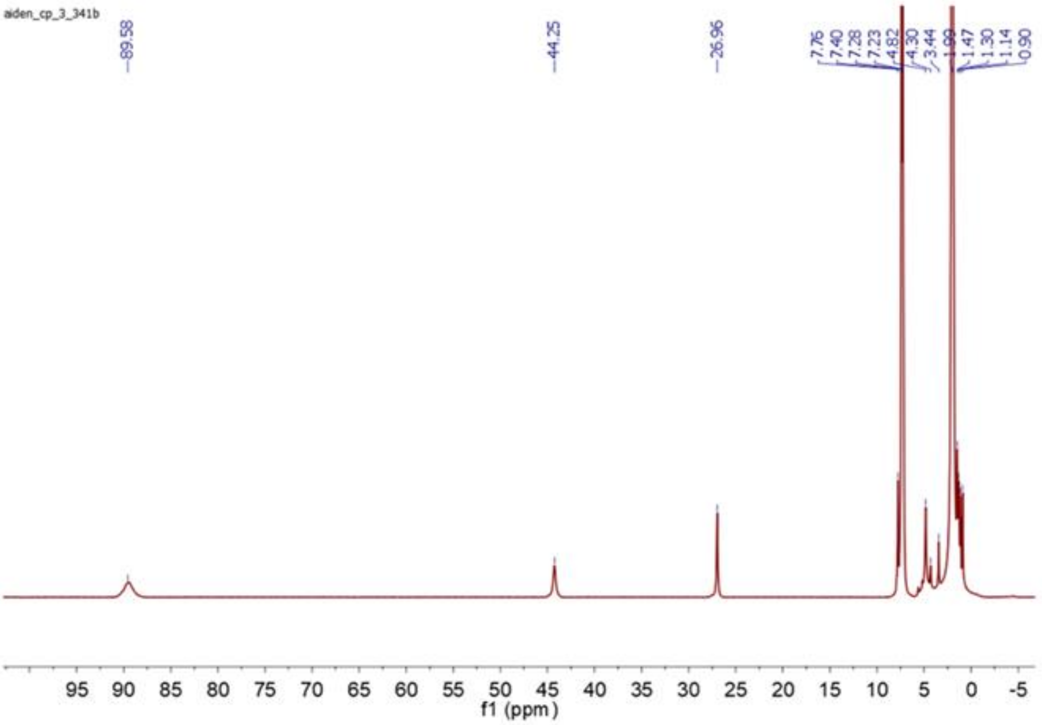

Figure S63. ^{1}H NMR for a reaction of **2** (2.5 mM) and Gomberg's dimer (10 mM) in CD_3CN at room temperature. The paramagnetically shifted signals at δ = -90, -45 and -26 ppm are characteristics of the precursor NTB-Fe-ACN complex (**1**). This indicates fluorine rebound by NTB-Fe_2F_3 leads to the formation of the NTB-Fe-ACN complex.

Table S1. Rate of halogen rebound comparison with known Fe-X systems.

Complex	X-atom transfer	k_2 ($M^{-1}s^{-1}$)	T (°C)	Reference
**2**	F•	1.24	-40	This work
Fe(F)(ttppc)[a]	F•	1.16×10^5	23	11
Fe(Cl)(ttppc) [a]	Cl•	1.34×10^3	23	11

[a]ttppc: 5,10,15-tris(2,4,6-triphenylphenyl)- corrolato^{3-}

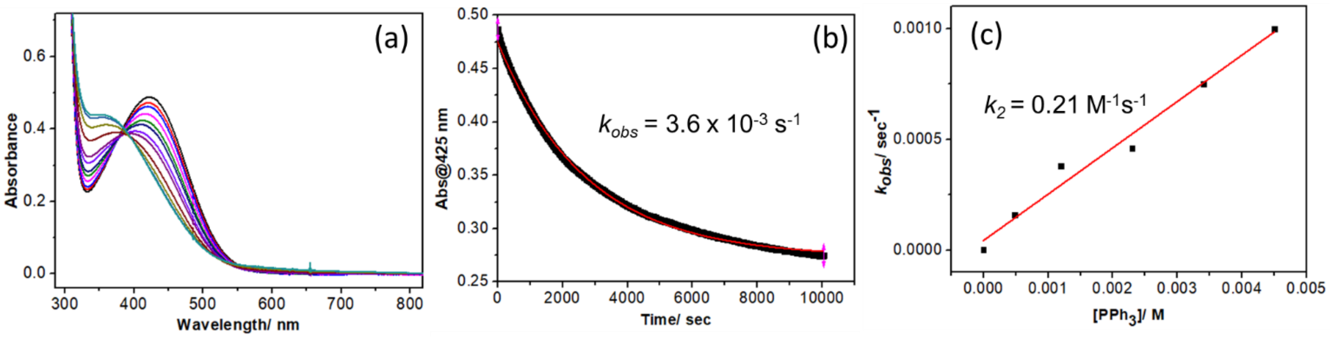

Figure S64. (a) UV-vis spectral change for a reaction of **2** (0.1 mM) and PPh_3 (5 mM) in CH_3CN at room temperature. (b) First order fitting for the reaction between **2** and PPh_3 to obtain the k_{obs}. (c) Plot of k_{obs} vs. PPh_3 concentration to calculate the second order reaction rate constant.

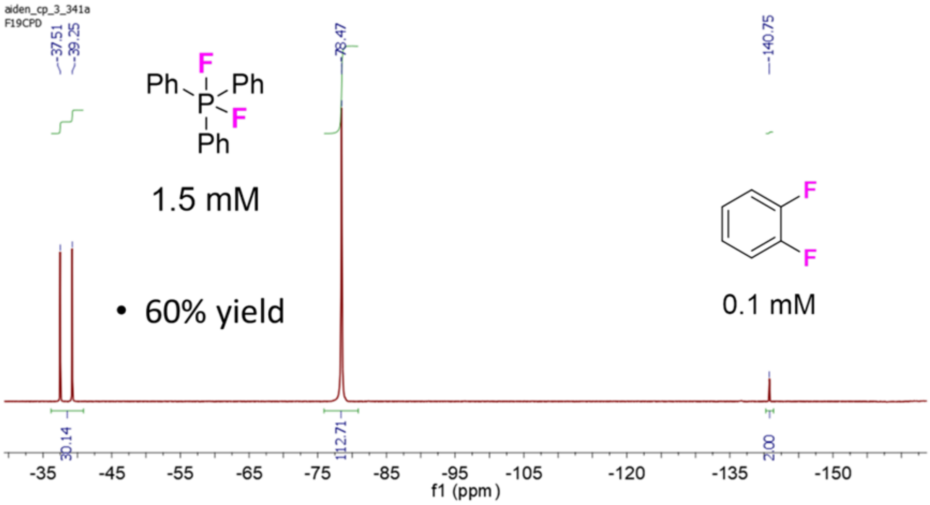

Figure S65. ^{19}F NMR of a mixture containing **2** (2.5 mM) and PPh_3 (25 mM) in CD_3CN. A doublet centred at δ = -38 ppm corresponds to the formation of the difluorotriphenyl-λ^5-phosphane. 1,2-difluoro benzene (δ = -140 ppm) was used as an internal standard for quantification of trityl fluoride (60% per [**2**]).

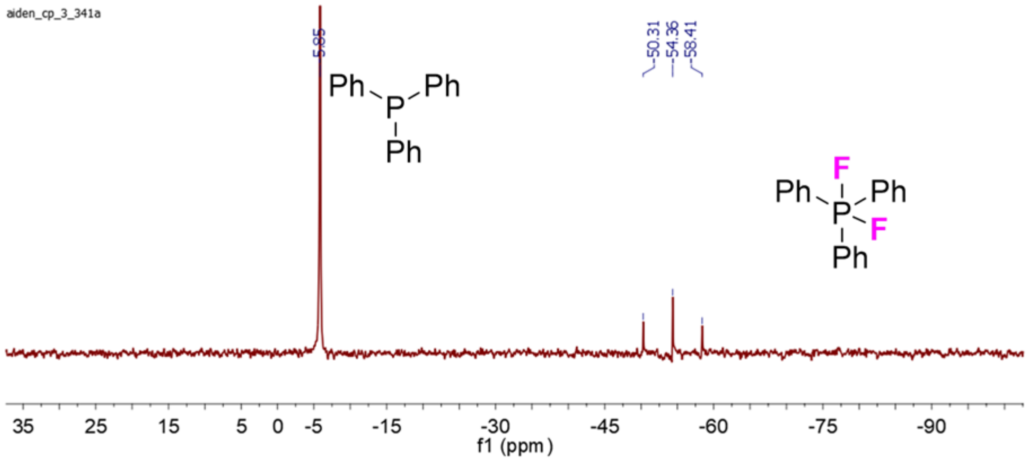

Figure S66. ^{31}P NMR of a mixture containing **2** (2.5 mM) and PPh_3 (25 mM) in CD_3CN. A triplet centred at δ =-54 ppm corresponds to the formation of the difluorotriphenyl-λ^5-phosphane. The singlet at δ = -6 ppm corresponds to the unreacted triphenylphosphine.

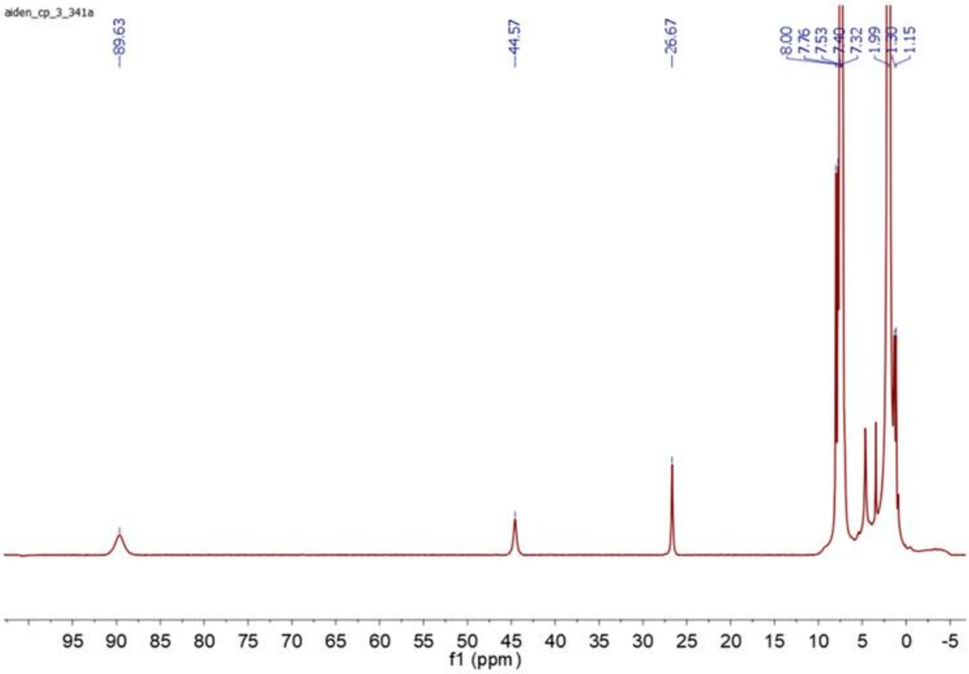

Figure S67. ^{1}H NMR of a mixture containing **2** (2.5 mM) and PPh_3 (25 mM) in CD_3CN. The paramagnetically shifted signals at δ = -90, -45 and -26 ppm are characteristics of the precursor **1**. This indicates that fluorine rebound by **2** leads to the formation of **1**.

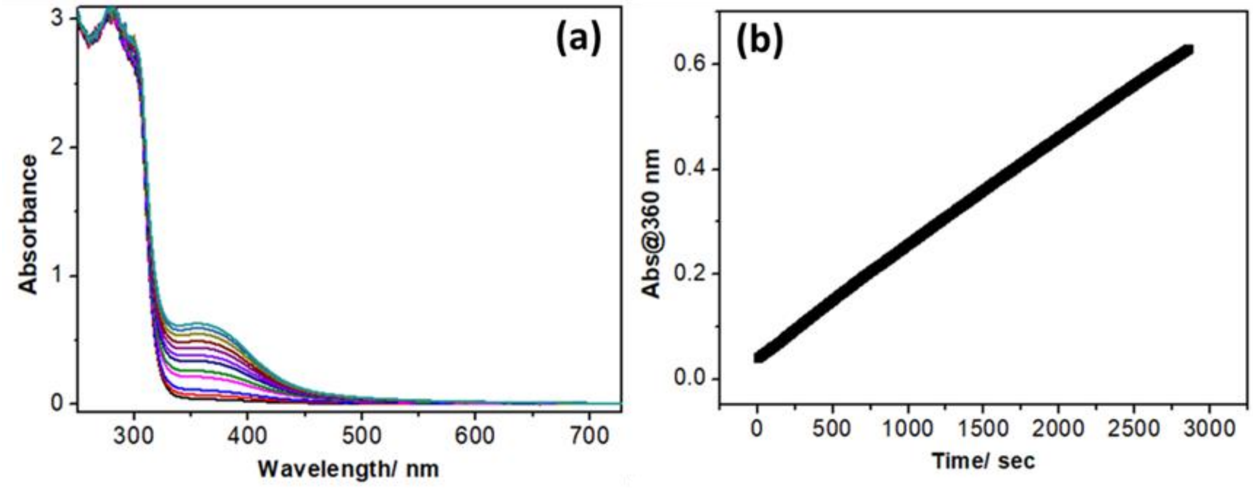

Figure S68. (a) UV-vis spectral change for a reaction of **1** (0.5 mM) and PPh_3 (5 mM) in CH_3CN at room temperature. The new species formed with electronic absorption feature at λ = 370 nm is very similar to that found in the post reaction mixture of **2** and PPh_3 (Figure S58). (b) Time trace for the formation of the new species with absorbance maximum at λ = 370 nm.

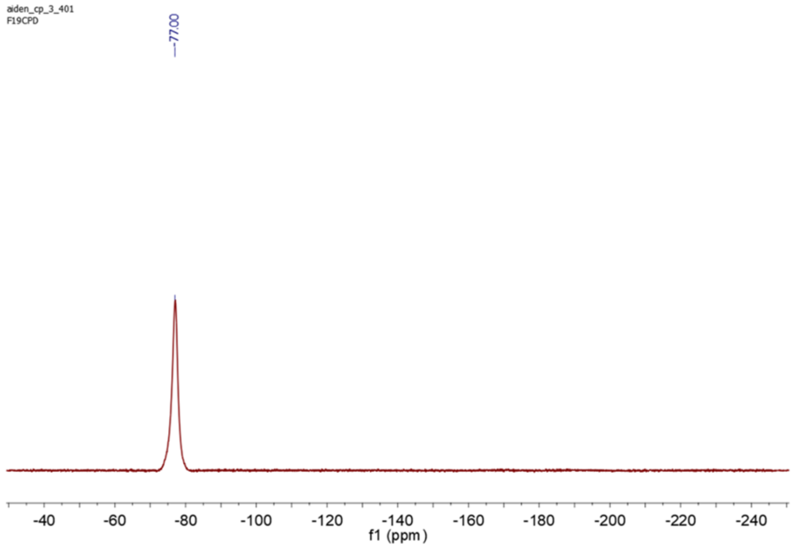

Figure S69. ^{19}F NMR of a mixture containing **3** (2.5 mM) and Gomberg's dimer (10 mM) in CD_3CN. A singlet at δ = -77 ppm corresponds to the triflate counter anion. There are no signals corresponding to the trityl fluoride.

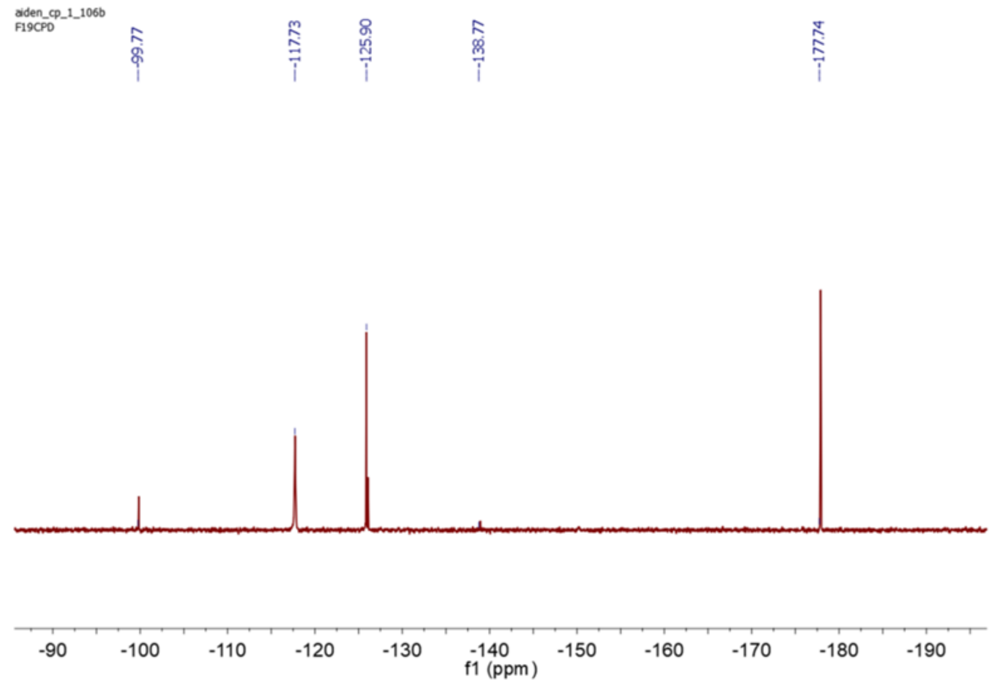

Figure S70. ^{19}F NMR of a mixture containing Gomberg's dimer (10 mM) and $PhIF_2$ (20 mM). The singlet at δ = -127 ppm corresponds to the trityl fluoride. However, additional signals at δ = -100, -117, -138 ppm may correspond to by-products originated form the FAT to resonance stabilized radicals of Gomberg' dimer. The signal at δ = -177 ppm is assigned to unreacted $PhIF_2$.

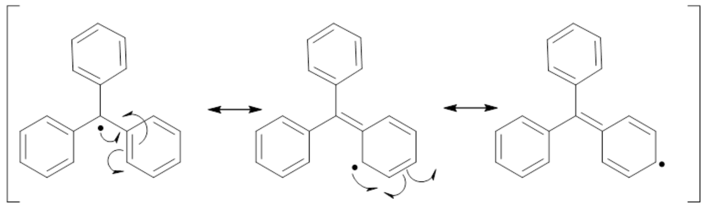

Figure S71. Resonance structures of trityl radical.

Table S2. Crystal data and structure refinement for **2**.

Empirical formula	$C_{27.38}H_{23.82}F_6FeN_{7.94}O_{4.50}S_{1.50}$
Formula weight	754.02
Temperature	100(2) K
Wavelength	0.71073 Å
Crystal system	Monoclinic
Space group	P 21/m
Unit cell dimensions	a = 11.9342(12) Å α = 90°
	b = 21.219(2) Å β = 112.667(2)°
	c = 13.3249(13) Å γ = 90°
Volume	3113.7(5) $Å^3$
Z	4
Density (calculated)	1.608 Mg/m^3
Absorption coefficient	0.672 mm^{-1}
F(000)	1535
Crystal size	0.239 x 0.147 x 0.132 mm^3
Theta range for data collection	1.656 to 25.998°
Index ranges	-14 ≤ h ≤ 14, -26 ≤ k ≤ 26, -16 ≤ l ≤ 16
Reflections collected	119753
Independent reflections	6292 [R(int) = 0.0822]
Completeness to theta = 25.242°	100.0%
Absorption correction	Semi-empirical from equivalents
Max. and min. transmission	0.7455 and 0.6680
Refinement method	Full-matrix least-squares on F^2
Data / restraints / parameters	6292 / 66 / 538
Goodness-of-fit on F^2	1.104
Final R indices [I>2sigma(I)]	R1 = 0.0565, wR2 = 0.1342
R indices (all data)	R1 = 0.0818, wR2 = 0.1431
Largest diff. peak and hole	0.988 and -0.663 $eÅ^{-3}$

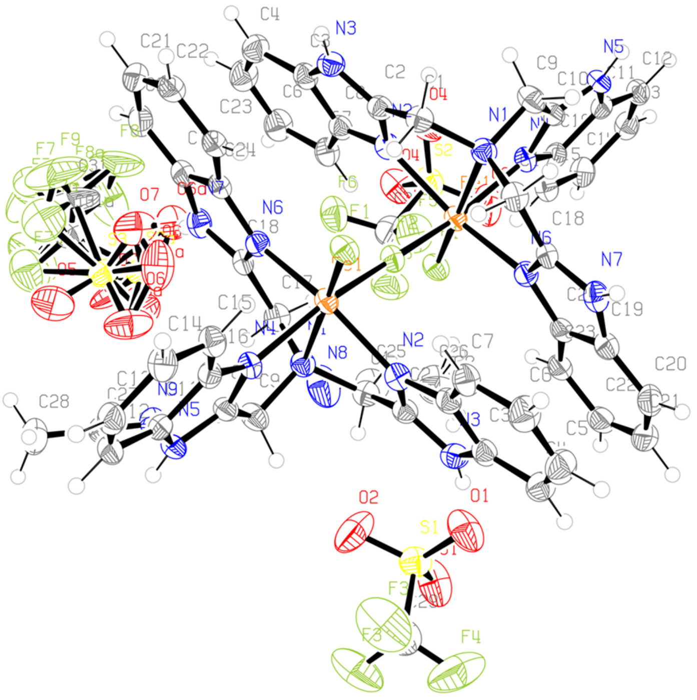

Figure S72: Single crystal X-ray structure for **2**

Table S3. Selected bond lengths (Å) and bond angles (°).

Fe(1)-F(1)	1.816(2)	C(21)-C(22)	1.398(6)
Fe(1)-F(2)	1.9830(5)	C(21)-H(21)	0.9500
Fe(1)-N(6)	2.044(3)	C(22)-C(23)	1.380(5)
Fe(1)-N(2)	2.050(3)	C(22)-H(22)	0.9500
Fe(1)-N(4)	2.071(3)	C(23)-C(24)	1.397(5)
Fe(1)-N(1)	2.320(3)	C(23)-H(23)	0.9500
F(1)-Fe(1)-F(2)	97.64(7)	F(2)-Fe(1)-N(4)	161.91(9)
F(1)-Fe(1)-N(6)	102.33(11)	N(6)-Fe(1)-N(4)	91.14(11)
F(2)-Fe(1)-N(6)	85.58(8)	N(2)-Fe(1)-N(4)	92.42(11)
F(1)-Fe(1)-N(2)	103.52(11)	F(1)-Fe(1)-N(1)	178.94(10)
F(2)-Fe(1)-N(2)	82.73(8)	F(2)-Fe(1)-N(1)	83.42(8)
N(6)-Fe(1)-N(2)	152.79(12)	N(6)-Fe(1)-N(1)	77.69(11)
F(1)-Fe(1)-N(4)	100.44(11)	F(2)-Fe(1)-N(4)	161.91(9)
N(2)-Fe(1)-N(1)	76.62(11)	Fe(1)#2-F(2)-Fe(1)	180.0
N(4)-Fe(1)-N(1)	78.50(11)		

References

(1) Bruker. *APEX3*, **2015**, *v2015.9-0*, Bruker AXS Inc., Madison, WI, USA.
(2) Bruker (2014/5). SADABS, B. A. I., Madison, Wisconsin, USA. (2014/5). SADABS, Bruker AXS Inc., Madison, Wisconsin, USA.
(3) Sheldrick, G. M. A short history of SHELX. *Acta Cryst.* **2008**, (A64), 112-122.
(4) Sheldrick, G. M. Crystal structure refinement with SHELXL. *Acta Cryst.* **2015**, (c71), 3-8.
(5) Stoll, S.; Schweiger, A. EasySpin, a comprehensive software package for spectral simulation and analysis in EPR. *J. Mag. Reson.* **2006**, *178* (1), 42-55. DOI: 10.1016/j.jmr.2005.08.013.
(6) Xue, X.-S.; Ji, P.; Zhou, B.; Cheng, J.-P. The Essential Role of Bond Energetics in C–H Activation/Functionalization. *Chem. Rev.* **2017**, *117* (13), 8622-8648. DOI: 10.1021/acs.chemrev.6b00664.
(7) Luo, Y.-R. *Comprehensive Handbook of Chemical Bond Energies (1st ed.)* **2007**, *Taylor & Francis Group*, DOI: 10.1201/9781420007282.
